# A Systems Biological Approach Reveals Multiple Crosstalk Mechanism between Gram-Positive and Negative Bacterial Infections: An Insight into Core Mechanism and Unique Molecular Signatures

**DOI:** 10.1371/journal.pone.0089993

**Published:** 2014-02-28

**Authors:** Muthukumar. R, Alexandar. V, Berla Thangam, Shiek S. S. J. Ahmed

**Affiliations:** 1 Department of Biotechnology, School of Bioengineering, SRM University, Tamil Nadu, India; 2 Faculty of Allied Health Sciences, Chettinad Academy of Research and Education, Tamil Nadu, India; 3 Department of Computational Biology, Faculty of Allied Health Sciences, Chettinad Academy of Research and Education, Tamil Nadu, India; Charité, Campus Benjamin Franklin, Germany

## Abstract

**Background:**

Bacterial infections remain a major threat and a leading cause of death worldwide. Most of the bacterial infections are caused by gram-positive and negative bacteria, which are recognized by Toll-like receptor (TLR) 2 and 4, respectively. Activation of these TLRs initiates multiple pathways that subsequently lead to effective immune response. Although, both the TLRs share common signaling mechanism yet they may exhibit specificity as well, resulting in the release of diverse range of inflammatory mediators which could be used as candidate biomolecules for bacterial infections.

**Results:**

We adopted systems biological approach to identify signaling pathways mediated by TLRs to determine candidate molecules associated with bacterial infections. We used bioinformatics concepts, including literature mining to construct protein-protein interaction network, prioritization of TLRs specific nodes using microarray data and pathway analysis. Our constructed PPI network for TLR 2 (nodes: 4091 and edges: 66068) and TLR 4 (node: 4076 and edges: 67898) showed 3207 common nodes, indicating that both the TLRs might share similar signaling events that are attributed to cell migration, MAPK pathway and several inflammatory cascades. Our results propose the potential collaboration between the shared signaling pathways of both the receptors may enhance the immune response against invading pathogens. Further, to identify candidate molecules, the TLRs specific nodes were prioritized using microarray differential expressed genes. Of the top prioritized TLR 2 molecules, 70% were co-expressed. A similar trend was also observed within TLR 4 nodes. Further, most of these molecules were preferentially found in blood plasma for feasible diagnosis.

**Conclusions:**

The analysis reveals the common and unique mechanism regulated by both the TLRs that provide a broad perspective of signaling events in bacterial infections. Further, the identified candidate biomolecules could potentially aid future research efforts concerning the possibility in differential diagnosis of gram-positive and negative bacterial infections.

## Introduction

Innate immune system is the first line of defense against invading pathogens that are highly conserved across species [1]. The principal characteristic of innate immunity is to recognize and to eliminate the invading pathogens. Recognition of pathogens is mediated by germ-line encoded pattern recognition receptors (PRRs) present in the outer membrane of innate immune cells [2]. These PRRs bind to evolutionarily conserved pathogen-associated molecular patterns (PAMPs) that play a key role in host defense mechanism. Among several PRRs, the toll-like receptors (TLRs) are the best characterized, in terms of the PAMPs recognition and activation of its corresponding pathways.

Several PAMPs have been noticed in gram-positive (gram +ve) and gram-negative (gram -ve) bacteria such as flagellin, lipoarabinomannan, lipoteichoic acid, lipopolysaccharide and peptidoglycan. In general, wide range of gram-positive bacteria containing peptidoglycan, lipoarabinomannan and lipoteichoic acid are predominantly recognized by TLR 2, and gram-negative bacteria with lipopolysaccharide by TLR 4 [3]. Upon PAMPs recognition, TLR 2 and 4 initiate multiple pathways that subsequently lead to complex inflammatory responses. Although, both the TLRs share common signaling mechanism yet they may exhibit specificity as well, resulting in the release of diverse range of inflammatory mediators like cytokines, cell adhesion molecules and growth factors [4], [5]. Importantly, cytokines play a pivotal role in the inflammatory cascade in response to TLRs activation.

Several studies have reported the altered levels of cytokines such as TNF α, IL-6, IL-8 and PCT in bacterial infection, which are suggested as diagnostic markers [6], [7]. However, the altered levels of these cytokines are not specific but also attributed to other disease conditions, irrespective of bacterial infection. In addition, the current gold standard culture positive method is time consuming, meantime the inflammatory cascade progresses to cause multi-organ failure leading to morbidity and mortality [8]. Hence, the identification of biomarkers specific to bacterial infection is needed for early and rapid diagnosis. Understanding the molecular mechanism between host recognition and immune response at the system level is a prerequisite for biomarker discovery [9]–[11].

In this study, we propose an integrative systems biological approach to uncover common and unique mechanisms between the receptors TLR 2 and TLR 4 that are activated with bacterial ligands. In particular, the potential application of this study is to identify early diagnostic markers for bacterial infections and to establish the bioavailability of these markers in body fluids for rapid detection.

## Results and Discussion

**Figure 1:**
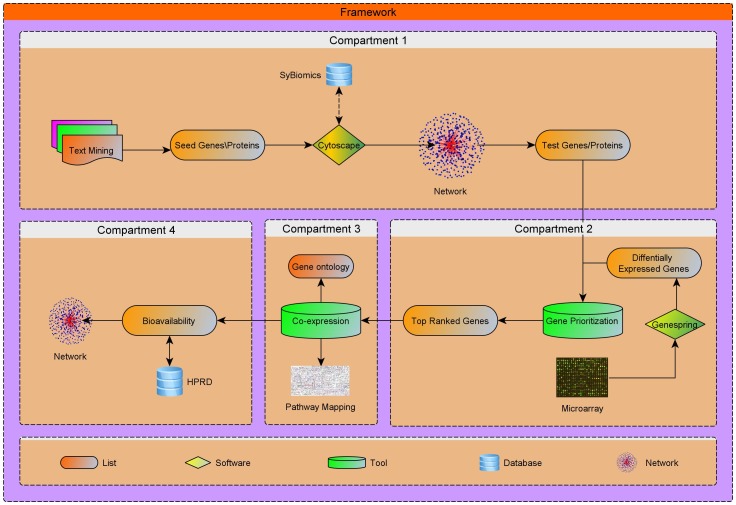
Systems biological framework for developing a receptor mediated network. The framework consists of four major compartments. The first compartment provides the seed genes/proteins from data mining and output as a network. The second compartment involves gene expression and prioritization of TLRs network proteins. The third compartment takes the top prioritized proteins as input to generate co-expression map, ontology and pathways. The final compartment of bioavailability network provides the proteins expressed in various body fluids for feasible detection.

**Figure 2:**
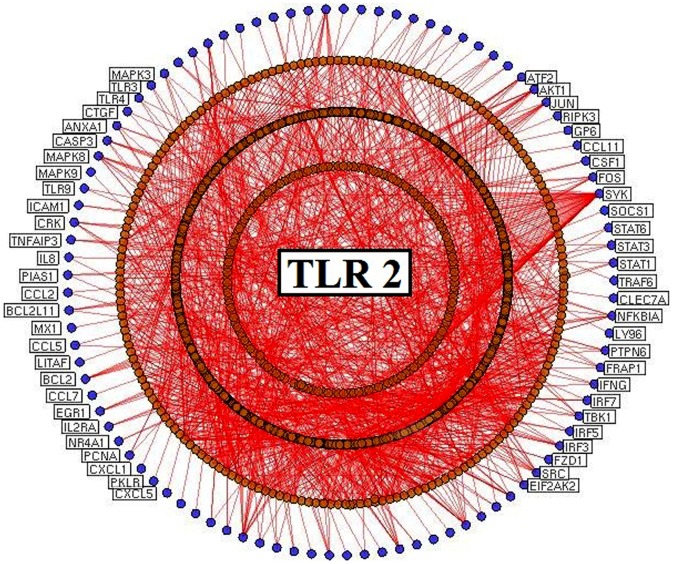
Protein-protein interaction network for Toll-like receptor 2. Literature mining tools extract the seed genes/proteins from research articles, and cytoscape generated the protein interaction network. The nodes (blue) represent seed proteins and orange-colored nodes correspond to seed interacting proteins. All the edges (red) represent interactions between the nodes.

**Figure 3:**
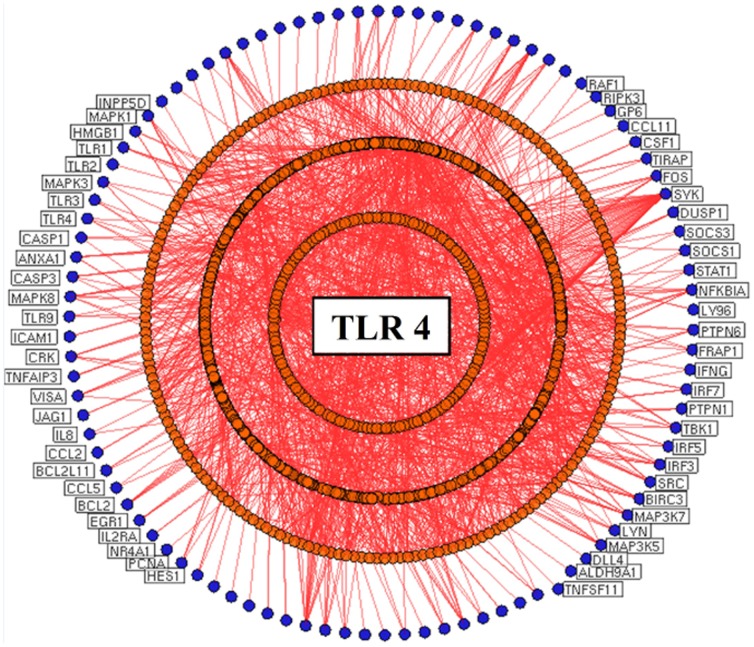
Protein-protein interaction network for Toll-like receptor 4. Literature mining tools extract the seed genes/proteins from research articles and cytoscape generated the protein interaction network. The nodes (blue) represent seed proteins and orange colored nodes represents seed interacting proteins. All the edges (red) represent interactions between the nodes.

In this study, a systems biological framework (Fig. 1) was developed to illustrate toll-like receptor 2 and 4 mediated signaling mechanism in gram-positive and negative bacterial infections. We used Medline ranker to retrieve articles relevance to the queries (query 1:“*TLR 2* or “*Toll-like receptor 2*”); (query 2: “*TLR 4* or “*Toll-like receptor 4*”) to obtain 968 and 988 efficient articles associated to search terms, respectively. From the retrieved results, the abstracts with p-value≤0.05 were selected and subjected to PESCADOR to extract 191 and 186 potential proteins for TLR 2 and 4. Further, the proteins were manually curated to remove duplicates and to create a potential seed list to generate PPI network for TLR 2 (Fig. 2) and TLR 4 (Fig. 3).

### Protein-Protein interaction network for the TLRs

The constructed TLR networks reveal the complex signaling interactions in TLR 2 and 4 in response to pathogen invasion. In TLR 2 network, there were 4091 nodes with 66068 edges, whilst there were 4076 nodes connected by 67898 edges in TLR 4 network. Further, the topological properties of the networks were analyzed for comparison, which include fundamental characteristics such as centrality, connectivity, heterogeneity and shortest path (Table 1) [12]–[14]. Topological analysis of both the networks showed that TLR 4 possesses more connecting components compared to TLR 2. For instance, in Table 1 the TLR 4 network had measurements of characteristic path length 2.363, network diameter 6 and a larger network centralization value 0.749, whilst the TLR 2 network was 2.388, 6 and 0.737, respectively. In particular, the network heterogeneity of TLR 2 was 2.080, while the heterogeneity of TLR 4 was measured to be 2.066. Overall, the variation in topological parameters describes the complexity and the existence of possible differential mechanism in TLR 2 and 4. Also, several common nodes were noticed between the networks that inclined to be crowded with higher interactions, suggesting the presence of collective regulatory mechanism in both TLRs [15].

**Table 1:** Network topology. The topology of TLR 2 and 4 networks.

Features	TLR 2	TLR 4
*Clustering coefficient*	0.256	0.260
*Connected components*	5	7
*Network Diameter*	6	6
*Network Radius*	1	3
*Network Centralization*	0.737	0.749
*Shortest Path Length*	16691312 (99%)	16560830 (99%)
*Characteristic Path length*	2.388	2.363
*Avg. number of neighbors*	32.299	33.316
*Number of Nodes*	4091	4076
*Network Density*	0.008	0.008
*Network Heterogeneity*	2.080	2.066
*Isolated Nodes*	3	6
*Number of self-loops*	0	0
*Multi-edge node pairs*	0	0

### Network association with bacterial infection

To further, investigate the relevance of the networks in bacterial infections, the microarray data set was obtained from GEO, accession number GSE6535 [16]. Gene Spring GX 7.3 microarray software was used to identify the differentially expressed genes in patients infected with bacteria (1) gram-positive *vs*. control, (2) gram-negative *vs.* control with the fold change≥2 and p-value≤0.05 as cut-off criteria [17], [18]. A total of 968 genes were significantly differentially expressed in gram +ve and 1024 in gram -ve bacterial infection (Table S1). Some of these differentially expressed genes encoding proteins were reported to influence the signaling mechanism. For instance, the differentially expressed gene, SF3B4 in TLR 2 was observed to interact with 49 neighbors in the network which was associated with Myd88 signaling pathway [19]. Also, PTPN1B in TLR 4 network was reported to have an essential role in regulation of MAPKs, NF-kappaB and IRF3 [20] which are key regulatory components against invading pathogens. These result evidence the potential significant relationship between network connectivity and bacterial infections.

### Protein prioritization with differential gene expression

In order to identify potential proteins in TLRs network, we used a strategy of protein prioritization based on functional characteristic features [21], [22]. The Toppgene tool [23] was used to prioritize candidate proteins by adopting the template of known gene-diseases association data as *training set* to rank the proteins in the *test set*. In this context, the differentially expressed genes in gram +ve infected patients were used as *training set* and the TLR 2 specific proteins were considered as *test set*. The 50 top ranked proteins (Table 2) were identified and subsequently taken for further analysis. Similar procedure was followed to determine the top 50 proteins (Table 2) in TLR 4 with their respective *training set* (gram -ve infected patients) and *test set.* Overall, these highly ranked candidate proteins play a vital role in bacterial pathogenesis that may provide insights into the pathways associated to TLR 2 and 4.

**Table 2:** Gene prioritization. List of top fifty genes from gene prioritization.

TLR 2		TLR 4	
API5*	NIN	AGFG1*	MMS19*
BRD8*	NRG1	ANAPC1*	NEUROD1*
CCNT1*	NUBP2	CDC20*	NFAT5*
CHD7*	PAGR1	CDC23*	NOTCH1
CIAO1	PCBD2	CKS1B*	NUP93*
CLIC4*	PDE12	CLK2*	POLR2L*
DPY30	PRDM1*	CPSF7*	PRPF19*
EIF2AK2*	PURA	DIMT1*	PRPF6*
ELF2*	RAD54L2*	DUSP1*	PRPF8
EPHA5*	RAP1GDS1*	EIF3L	PTBP3*
FHOD1*	RUSC2*	FANCF	RBMX
FLI1*	SNX4*	FANCG*	RCC1
FOXA1*	SOX1	FLOT2	RRAS2*
FTSJ3*	SP100*	FRYL*	RREB1
GPM6B*	SRSF1*	HDAC7	SMAD9
IVNS1ABP*	STK25*	HES6	SNRPD1*
JAZF1	STRBP	LEF1*	SON*
KDM4C*	TAB2*	LRCH1	TBC1D9
LRIG1	TMCO3	MAML2	TCF12*
MED14*	TRIM24	MAP2K6*	TFDP1*
MOB4*	TTK*	MARCKSL1*	TIAL1
MOXD1*	WDR26*	MAU2	TXNL4A
NAP1L4	WDR82*	MCM4*	UPF1*
NCBP1*	ZBTB5*	MDN1*	WLS*
NCOA7	ZFPM2*	MED25	XPO5*

*coexpressed genes.

### Co-expression and crosstalk mechanisms

**Figure 4:**
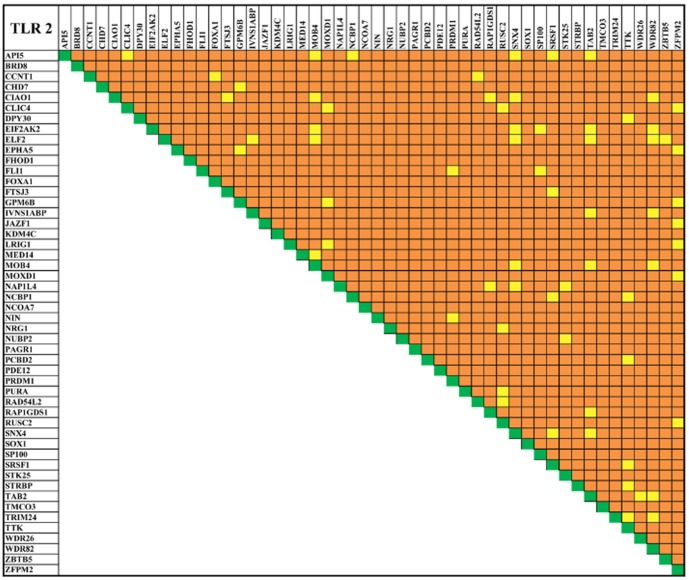
Toll-like receptor 2 co-expression map. The map represents the co-expressed of prioritized top 50 genes of TLR 2 network. The plot represent the wide range of expression pattern between the genes indicated as co-expression (yellow), no co-expression (orange) and self-expression (green).

**Figure 5:**
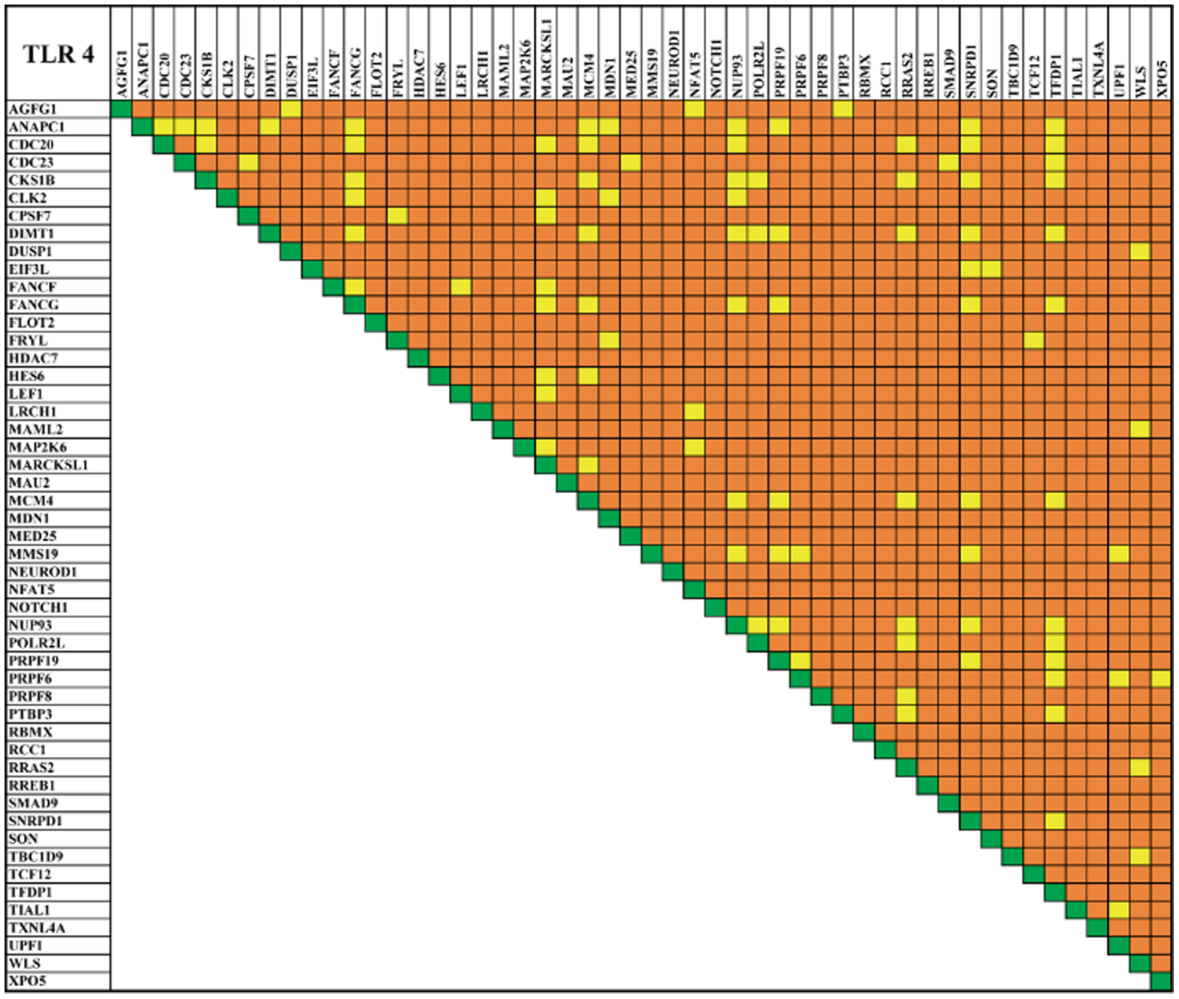
Toll-like receptor 4 co-expression map. The map represents the co-expressed of prioritized top 50 genes of TLR 4 network. The plot represent the wide range of expression pattern between the genes indicated as co-expression (yellow), no co-expression (orange) and self-expression (green).

**Figure 6:**
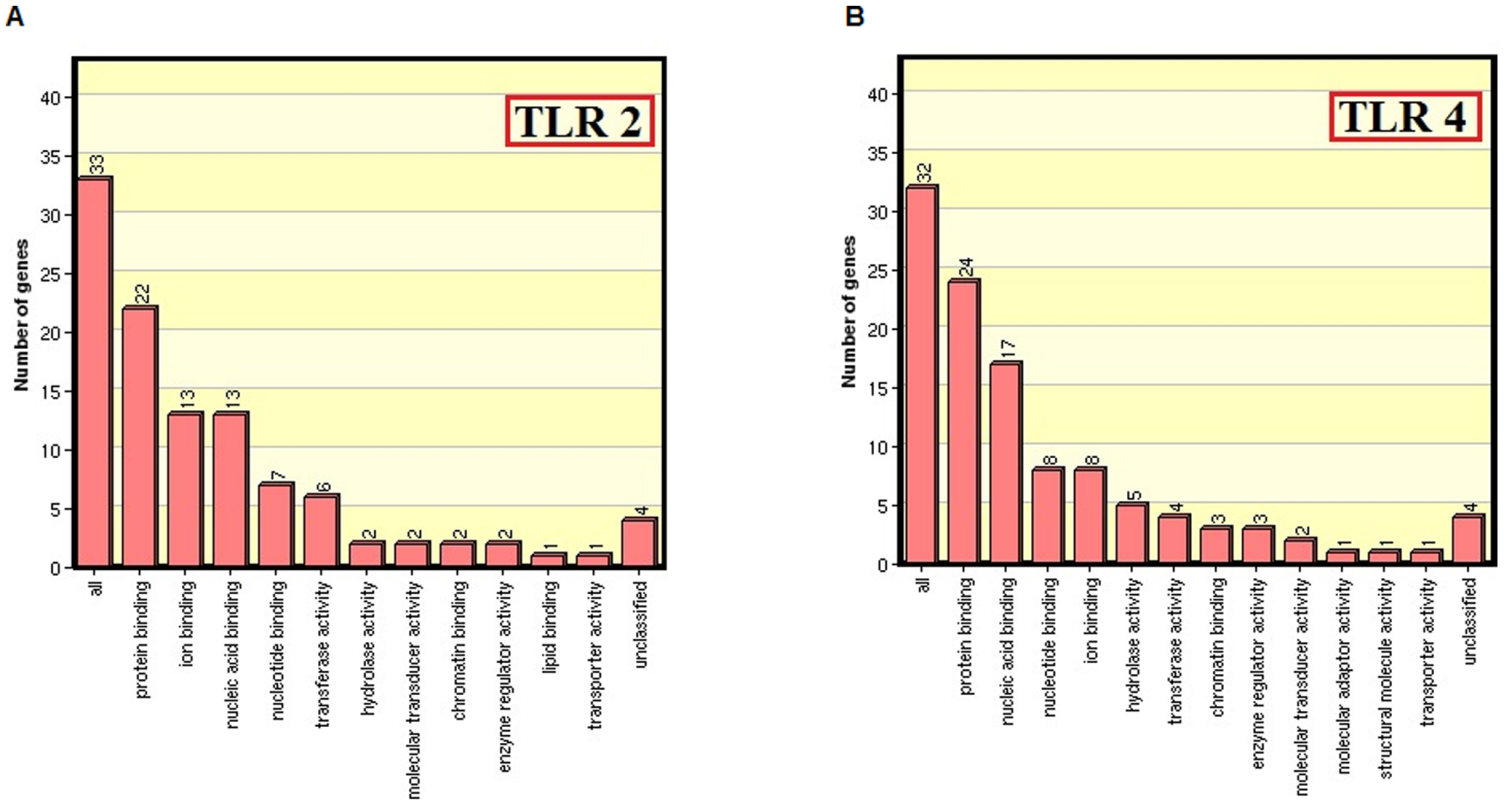
Gene ontology. The ontological classification of proteins associated to TLR2 (A) and TLR4 (B) receptors were extracted from the gene ontology database. The inputted 33 proteins of TLR2 and 32 proteins of TLR4 showed a range of functional distributions. Of which, protein binding activity was mostly attributed by the genes that influence signal transduction in bacterial infections.

**Figure 7:**
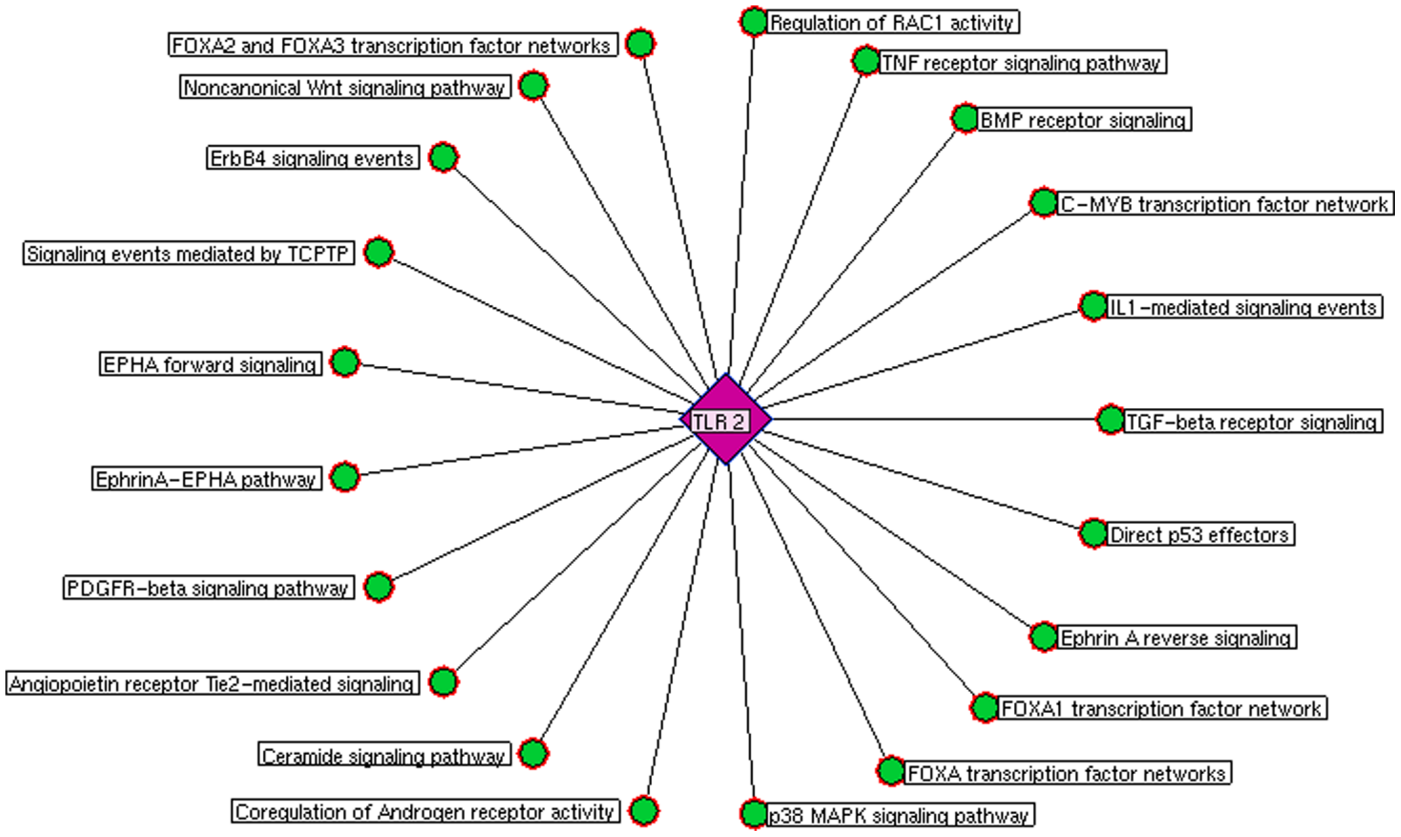
Toll-like receptor 2 mediated pathways. The prioritized co-expressed genes\proteins were assigned to biological pathways which showed twenty one significant pathways associated to TLR 2 network.

**Figure 8:**
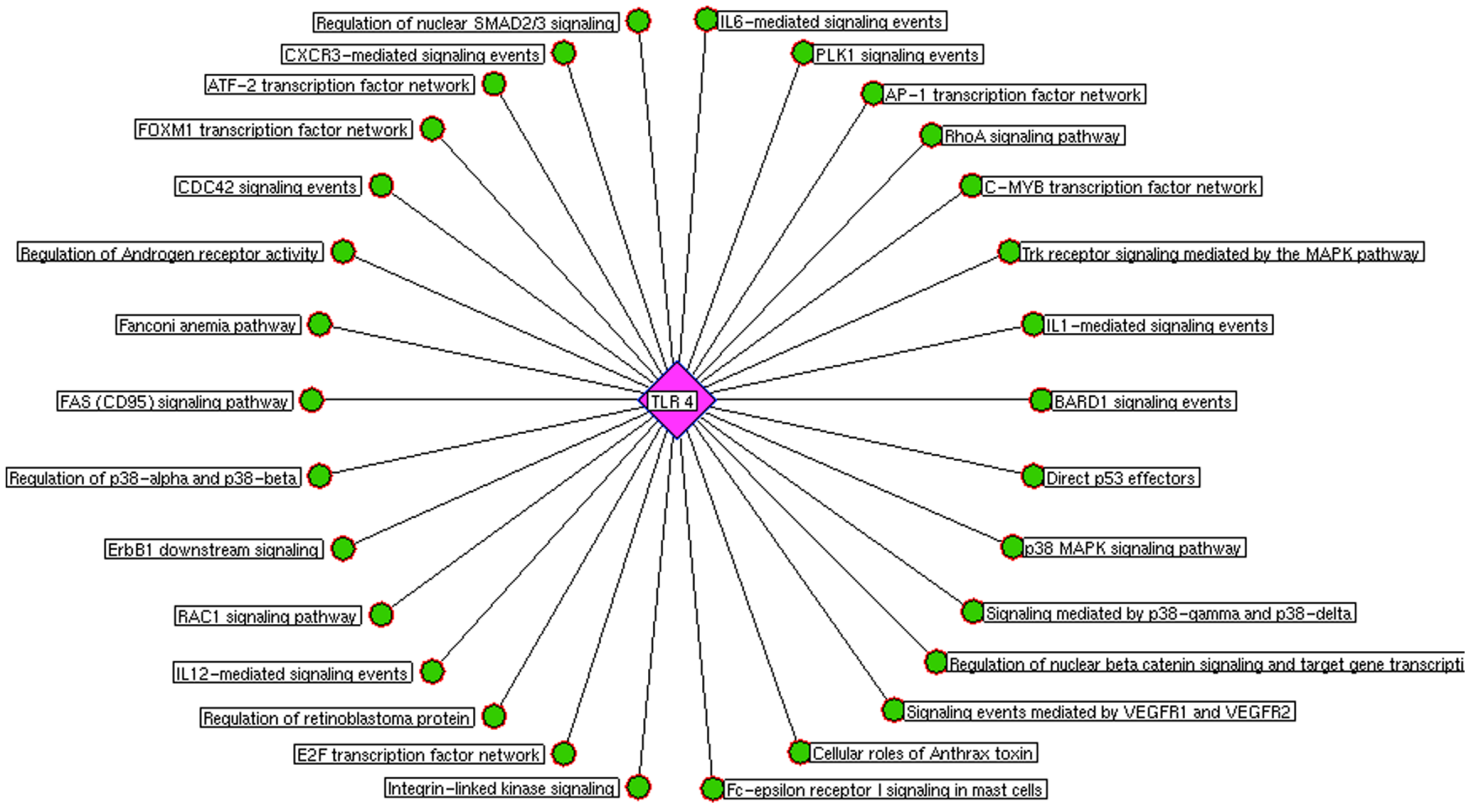
Toll-like receptor 4 mediated pathways. The prioritized co-expressed genes\proteins were assigned to biological pathways which showed thirty significant pathways associated to TLR 4 network.

**Figure 9:**
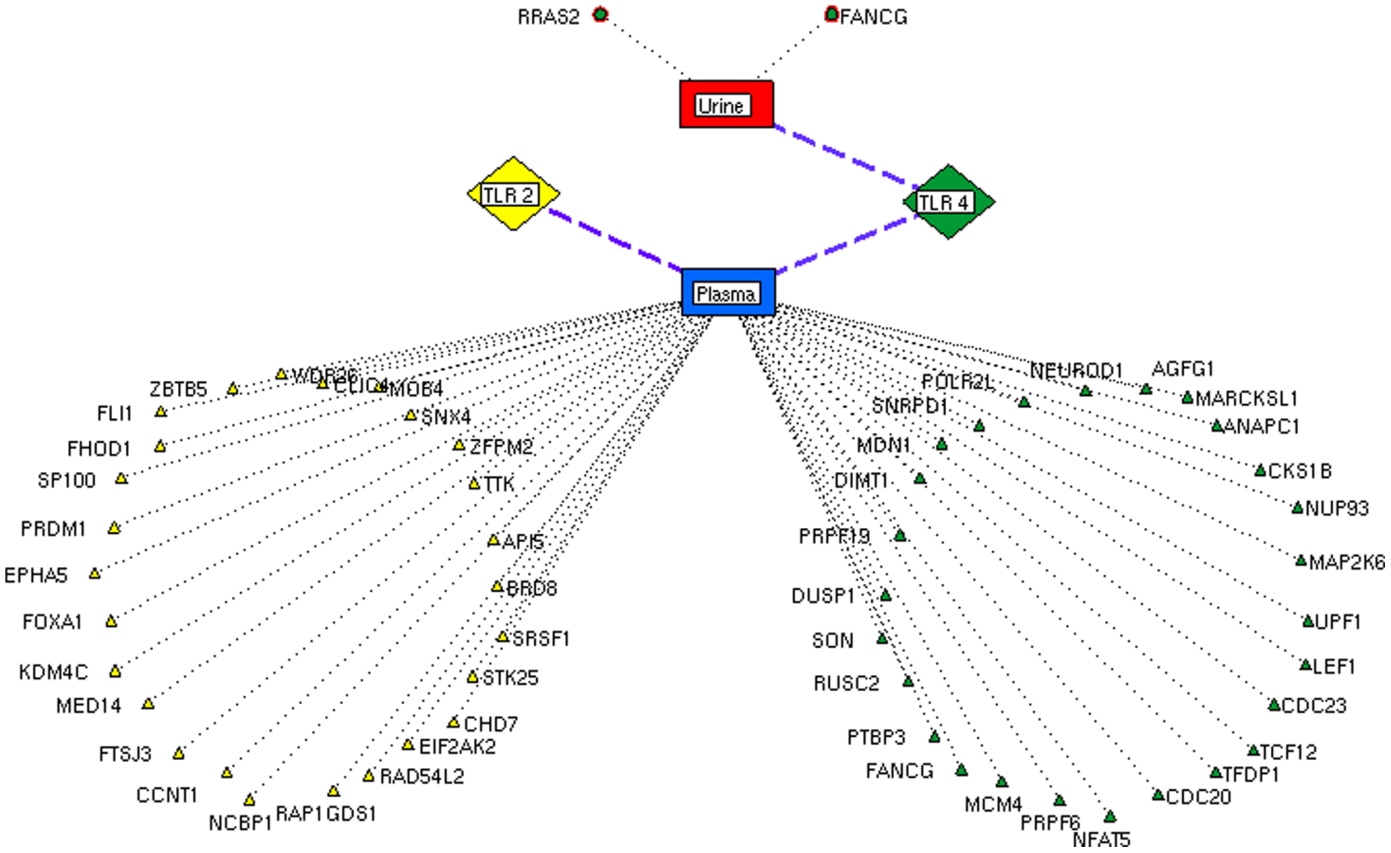
Bioavailability of candidate biomolecules in body fluids. Bioavailability network provides the proteins expressed in plasma and urine for feasible detection. The proteins expressed in fluids represented as triangle for plasma, colored yellow (Gram positive), green (Gram negative) and proteins expressed in urine represented as ellipse for Gram negative. No specific proteins were found in urine for gram positive infections.

Pathway analysis is crucial in understanding the molecular events involved in pathological conditions [24]. Most of these processes are the results of multiple genes working co-operatively in pathways. Several studies suggest that the genes co-expression is essential for pathway regulation [25]–[27]. Hence, prior to pathway analysis, we demonstrated the gene\protein co-expression pattern for the prioritized proteins using Genefriends [28] tool. Among the analyzed protein (Table 2), 33 of TLR2 and 32 of TLR 4 were co-expressed (Fig. 4 and 5). These co-expressed proteins were further considered for gene ontology (Fig. 6) and pathway analysis to reveal the molecular and signaling mechanism in bacterial infection. The nature curated PID database was executed to identify the pathway mechanisms for TLR 2, TLR 4 and TLR2U4 (*common proteins between TLR 2 and TLR 4*) which showed an existence of key signaling events behind the receptors (Fig. 7 and 8). In addition, TLR2U4 resulted pathways suggest the occurrence of a common downstream process in both the receptors (Table S2) [29], [30]. For instance, the regulations of p38-alpha and p38-beta pathways were found to be linked with both the TLRs [31]. In addition, these pathways were associated with mitogen activated protein kinase (MAPK) pathway [32] that attributes to molecular cascades such as apoptosis, inflammation, cell migration and differentiation. MAPK cascades are signaling modules that transduce extra stimuli into intra cellular response through NFκB in the host [32]. Activation of MAPKs is an initial signaling event after PAMPs recognition that mediates NFκB, which in turn induce genes of IL-1 family of inflammatory cytokines [33]. Interestingly, IL1 mediated pathway was identified as a common pathway between both the receptors. Overall, the pathway analysis establishes that the recognition of specific PAMPs by respective TLRs will activate separate and shared pathways. It is suggested that the potential collaboration between the shared signaling pathways of both the receptors may enhance the transcriptional and cellular response against invading pathogenic microbe. Activation of the responses exhibits several inflammatory mediators which have been extensively studied and reported to be the candidate markers for bacterial infections [34]. Though many markers have been reported, their application towards clinical diagnosis is still juvenile. In addition, most of these markers failed to differentiate bacterial infections from other immunological conditions. Hence, a system-level approach would be promising in understanding complex mechanisms of host response to determine candidate markers for diagnosis. Furthermore, the bioavailability of the candidate markers driven from such approach need to be validated in body fluids (plasma and urine) to develop rapid diagnostic procedures. In this context, the analysis of co-expressed proteins in gram +ve (TLR 2) and gram -ve (TLR 4) bacterial infections were found in plasma and urine (Fig. 9). In gram -ve infection, 26 proteins were expressed in plasma and two were found in urine. Similarly, for gram +ve infection, 26 proteins were expressed in plasma whereas no candidate markers were found in urine indicating that blood plasma could be a suitable medium for detection of gram +ve and gram -ve bacterial infections. Though, the identified individual candidate markers could help in differential diagnosis, the combination of these markers would promote the development of multi-marker system for effective detection and diagnosis of bacterial infections.

## Conclusions

In conclusion, a systems biological knowledge about the host signaling mechanism upon activation of TLR 2 and 4 will pave the way towards identification of new candidate biomolecules associated to gram-positive and negative bacterial infections. In addition, our result demonstrates that the existence of common and unique pathway mechanism, which could be used to formulate new therapeutic approaches to improve disease condition. Moreover, experimental and clinical studies are a prerequisite to accomplish these observations.

## Materials and Methods

### Text mining

To extract a more efficient set of PubMed abstracts relevant to our study, Medline Ranker [35], a text mining tool was used. Medline Ranker computes most discriminating words for the given query, and compares with the abstracts available at Medline database and ranks them based on word relevancy. In this context, we retrieved a list of top ranked abstracts with its corresponding PubMed accession number (PMID) for the keywords, “*TLR 2*” or “*Toll-like receptor 2*”, “*TLR 4*” or “*Toll-like receptor 4*” with the search criteria between the years 2003 to 2013. Further, these selected PMID were imported into the PESCADOR [36], to extract genes/proteins associated to TLR 2 and 4, respectively. These extracted proteins were curated to remove duplicates from the identified potential seed proteins of TLR 2 and 4.

### Network generation and topological analysis

Seed proteins were subjected to Cytoscape software [37] to generate protein-protein interaction networks (PPI) for TLR 2 and 4, respectively. Each network was created using BisoGenet plug-in [38] with experimentally validated PPIs from six databases (BIOGRID, INTACT, MINT, DIP, BIND and HPRD) to expand the seed proteins into a network. However, these networks were limited to first interacting neighbors of seed proteins to have the direct impact on TLR 2 and TLR 4 activation and regulation. In addition, self-loops and duplicated edges were removed, and the topological features were calculated by the network analyzer to determine the network property.

### Gene expression data

Microarray data was retrieved from NCBI, Gene Expression Omnibus (GEO) repository [39] using the accession number GSE 6535. The data include four sample sets (i) *healthy individuals* (ii) *sepsis patients infected with gram +ve bacteria* (iii) *sepsis patients infected with gram -ve bacteria* and (iv) *sepsis patients infected with co-bacterial infection*. All samples in the data set was log transformed, normalized (perchip: normalization to the 75 percentile shift; per gene: normalization to median across all samples) and filtered based on fold change (two fold). Further, we applied analysis of variance (ANOVA) to determine the statistical significance (p≤0.05) of differentially expressed genes using Gene Spring GX.7.3 (Agilent Technologies Inc., Santa Clara, California) software.

### Gene-prioritization

Toppgene [23] was used to prioritize the key molecules that play a vital role in TLR 2 and 4 signaling mechanism. Toppgene tools prioritize the candidate genes/proteins in the test set based on molecular functional similarity against training gene list. The microarray differentially expressed genes were considered as *training set* and systems biology driven proteins that are specific to TLR 2 and TLR 4 were input as *test sets*. Further, the top fifty prioritized proteins in the *test set* of TLR 2 and TLR 4 were identified and subjected to co-expression analysis.

### Co-expression analysis

The co-expression analysis was carried out using GeneFriends [28] tool for the identified top 50 ranked proteins. The GeneFriends uses a vote-counting method to rank co-expressed genes by creating co-expression map from the genomic data at NCBI GEO data set. In this context, the top ranked proteins for TLR 2 and TLR 4 were filtered based on co-expression and subjected to gene ontology and pathway analysis.

### Pathway analysis

In order to understand the functional and signaling mechanism of the proteins, we input the co-expressed proteins into nature curated Pathway Interaction Database (PID) [40]. The pathway interaction database identifies canonical pathways associated with a given list of proteins to determine the key regulating molecules in TLR 2 and TLR 4 signaling mechanism.

### Candidate proteins in body fluids

To facilitate feasible diagnosis, we evaluated the availability of co-expressed genes encoding proteins in various biological fluids [41]. Protein expressed in plasma and urine were retrieved from the human protein reference database (HPRD) [42] and mapped to the co-expressed protein for the feasible diagnosis.

## Supporting Information

Table S1 **Significantly differentially expressed genes.** Microarray analysis of bacterial infections patients showed a significantly differentially expressed for Gram-positive and Gram-negative bacterial infection, which considered as training set for gene prioritization.
(PDF)

Table S2 **Common pathways between TLR 2 and TLR 4.** **I**dentified common pathways (TLR2U4) mediated by both the TLRs suggests the occurrence of a common downstream process in bacterial infections.
(PDF)
