# Supplementary material for: A Systems Biological Approach Reveals Multiple Crosstalk Mechanism between Gram-Positive and Negative Bacterial Infections: An Insight into Core Mechanism and Unique Molecular Signatures
Source: PLoS One. 2014 Feb 28;9(2):e89993. doi: 10.1371/journal.pone.0089993 (PMC3938579; doi:10.1371/journal.pone.0089993)
Supplement: Table S1 — Significantly differentially expressed genes. Microarray analysis of bacterial infections patients showed a significantly differentially expressed for Gram-positive and Gram-negative bacterial infection, which considered as training set for gene prioritization. (PDF) [file pone.0089993.s001.pdf]

Additional file 1: Significantly differentially expressed genes

GSE 6535: Gram positive bacterial infected patients - Differentially expressed genes

TLR 2 Training set

ID	Gene.title	P.Value
AB001451	SHC (Src homology 2 domain containing) transforming protein 2	2.82E-02
AB002301	microtubule associated serine/threonine kinase family member 4	1.69E-02
AB002306	chromodomain helicase DNA binding protein 9	4.12E-02
AB002319	zinc finger, FYVE domain containing 26	3.67E-02
AB002340	tetratricopeptide repeat and ankyrin repeat containing 1	1.99E-02
AB002365	prune homolog 2 (Drosophila)	3.21E-02
AB002368	exportin 6	1.68E-03
AB002437	EPB41L4A antisense RNA 1	1.95E-03
AB002444	toll-like receptor adaptor molecule 2	3.21E-02
AB007859	zinc finger, ZZ-type with EF-hand domain 1	3.12E-02
AB007869	ribosomal RNA processing 8, methyltransferase, homolog (yeast)	3.24E-02
AB007896	prolyl endopeptidase-like	4.25E-02
AB007916	solute carrier family 35, member E2B	5.43E-03
AB007928	OTU domain containing 3	1.54E-02
AB007932	plexin A2	9.02E-03
AB007950	transmembrane and coiled-coil domain family 2	3.07E-02
AB007965	cysteine/histidine-rich 1	8.22E-03
AB011112	neurobeachin-like 2	4.86E-02
AB011171	pleckstrin homology domain containing, family G (with RhoGef domain) member 3	1.80E-02
AB011173	lysine (K)-specific demethylase 1A	2.48E-02
AB011539	multiple EGF-like-domains 6	5.13E-04
AB014524	exophilin 5	1.19E-02
AB014548	PDS5, regulator of cohesion maintenance, homolog A (S. cerevisiae)	1.91E-02
AB014550	structural maintenance of chromosomes flexible hinge domain containing 1	1.68E-02
AB014557	obscurin-like 1	1.92E-02
AB015349	GRB2 associated, regulator of MAPK1-like	1.30E-02
AB018260	Rho-related BTB domain containing 2	1.78E-02
AB018292	dendrin	1.70E-02

AB018319	UFM1-specific ligase 1	2.26E-02
AB018322	transmembrane and coiled-coil domain family 1	1.82E-03
AB018324	salt-inducible kinase 2	5.71E-03
AB018325	ArfGAP with RhoGAP domain, ankyrin repeat and PH domain 1	8.02E-04
AB020713	nucleoporin 210kDa	9.36E-03
AB023147	KIAA0930	4.50E-03
AB023161	dynein, axonemal, heavy chain 7	1.87E-02
AB023190	microtubule associated serine/threonine kinase 1	3.11E-02
AB023195	additional sex combs like 1 (Drosophila)	7.23E-03
AB023230	FERM domain containing 4B	2.92E-02
AB023420	heat shock 70kDa protein 4	1.17E-02
AB025194	protein tyrosine phosphatase, non-receptor type 23	4.53E-02
AB026436	dual specificity phosphatase 10	4.86E-02
AB026542	WAS protein family, member 2	2.53E-02
AB028449	dicer 1, ribonuclease type III	3.08E-02
AB028963	MON2 homolog (S. cerevisiae)	7.54E-03
AB032953	teneurin transmembrane protein 2	3.36E-02
AB032967	zinc finger protein 473	9.94E-03
AB032969	KIAA1143	9.22E-04
AB032986	ISY1-RAB43 readthrough	1.91E-02
AB032988	thioredoxin-related transmembrane protein 4	2.06E-02
AB033046	glutamate receptor, ionotropic, delta 1	1.04E-02
AB033073	sulfatase 2	2.58E-04
AB033083	KIAA1257	2.25E-02
AB033096	alanyl-tRNA synthetase 2, mitochondrial	1.51E-02
AB033100	phosphatase domain containing, paladin 1	3.81E-02
AB033101	filamin A interacting protein 1	2.52E-02
AB033110	inturned planar cell polarity protein	2.37E-02
AB037716	SH3 and PX domains 2B	1.15E-02
AB037722	HECT, C2 and WW domain containing E3 ubiquitin protein ligase 2	2.57E-02
AB037730	kelch-like family member 13	4.68E-02
AB037735	Rho GTPase activating protein 28	2.72E-02
AB037743	TBC1 domain family, member 14	9.37E-03

AB037785	microtubule associated monooxygenase, calponin and LIM domain containing 3	1.98E-02
AB037792	KIAA1109	6.32E-03
AB037820	membrane-associated ring finger (C3HC4) 4, E3 ubiquitin protein ligase	2.04E-02
AB037836	phosphatidylinositol-3,4,5-trisphosphate-dependent Rac exchange factor 1	1.22E-03
AB037851	KIAA1430	4.03E-02
AB039920	KCNQ1 downstream neighbor (non-protein coding)	4.76E-02
AB040812	p21 protein (Cdc42/Rac)-activated kinase 7	2.21E-02
AB040899	endogenous retrovirus group 3, member 2	3.27E-02
AB040929	contactin 3 (plasmacytoma associated)	3.79E-02
AB040954	GTPase activating protein and VPS9 domains 1	4.13E-02
AB040968	hyperpolarization activated cyclic nucleotide-gated potassium channel 3	3.52E-02
AB046765	fibrosin-like 1	2.36E-02
AB046768	erythrocyte membrane protein band 4.1 like 5	8.68E-03
AB046777	AT rich interactive domain 2 (ARID, RFX-like)	3.43E-02
AB046781	uveal autoantigen with coiled-coil domains and ankyrin repeats	2.06E-02
AB046782	protocadherin 18	5.12E-03
AB046815	DEAD (Asp-Glu-Ala-Asp) box polypeptide 55	2.25E-02
AB046822	NCK-associated protein 5-like	1.70E-02
AB046830	mesoderm induction early response 1 homolog (Xenopus laevis)	8.79E-03
AB046842	protein phosphatase 4, regulatory subunit 4	7.06E-03
AB046859	obscurin, cytoskeletal calmodulin and titin-interacting RhoGEF	1.72E-02
AB046861	ankyrin repeat domain 36	1.99E-02
AF000672	ELK1, member of ETS oncogene family	3.80E-02
AF005082	long intergenic non-protein coding RNA 302	1.42E-02
AF007132	abhydrolase domain containing 5	3.22E-02
AF007153	v-erb-a erythroblastic leukemia viral oncogene homolog 4 (avian)	3.16E-02
AF007835	chromosome 1 open reading frame 116	4.54E-02
AF010236	sarcoglycan, delta (35kDa dystrophin-associated glycoprotein)	4.67E-03
AF024699	zinc finger and SCAN domain containing 9	1.48E-02
AF029777	K(lysine) acetyltransferase 2A	4.63E-02
AF035282	chromosome 1 open reading frame 21	3.53E-02
AF039023	RAN binding protein 6	2.35E-02
AF040964	HAUS augmin-like complex, subunit 3	3.95E-02

AF043324	N-myristoyltransferase 1	2.47E-02
AF049140	ubiquitin-conjugating enzyme E2 variant 2	4.62E-02
AF052146	family with sequence similarity 105, member A	7.86E-03
AF052148	X-ray repair complementing defective repair in Chinese hamster cells 6	7.47E-03
AF052497	myozenin 3	2.93E-02
AF055084	G protein-coupled receptor 98	4.41E-02
AF056450	death-associated protein	1.09E-02
AF064078	insulin receptor-related receptor	4.60E-02
AF069307	solute carrier family 5 (sodium-dependent vitamin transporter), member 6	3.24E-02
AF070525	GrpE-like 1, mitochondrial (E. coli)	4.14E-02
AF070617	family with sequence similarity 172, member A	3.78E-02
AF070637	N-terminal EF-hand calcium binding protein 2	2.61E-02
AF075028	fragile X mental retardation, autosomal homolog 1	1.52E-02
AF075029	poly(A) polymerase gamma	2.97E-02
AF075085	oncoprotein induced transcript 3	7.12E-03
AF085836	myeloid/lymphoid or mixed-lineage leukemia (trithorax homolog, Drosophila); translocated to, 4	3.30E-03
AF085855	pregnancy-associated plasma protein A, pappalysin 1	3.29E-02
AF085877	SSSCA1 antisense RNA 1 (head to head)	8.40E-03
AF085987	family with sequence similarity 101, member B	9.55E-03
AF086040	forkhead box P2	1.37E-02
AF086050	metal response element binding transcription factor 2	2.81E-02
AF086098	chromosome 2 open reading frame 72	1.63E-02
AF086124	small lysine-rich protein 1	3.21E-02
AF086127	zinc finger protein 641	4.94E-02
AF086175	transmembrane protein 245	1.77E-02
AF086189	Ras association (RalGDS/AF-6) and pleckstrin homology domains 1	1.80E-02
AF086198	hexokinase 2	1.63E-02
AF086345	splA/ryanodine receptor domain and SOCS box containing 4	4.52E-03
AF086386	muskelin 1, intracellular mediator containing kelch motifs	1.31E-03
AF086390	signal peptide, CUB domain, EGF-like 2	1.00E-02
AF086406	CUE domain containing 2	5.39E-03
AF086428	zinc finger protein 585A	1.14E-02
AF086480	keratin 74	4.89E-02

AF086529	GRB2 associated, regulator of MAPK1	4.27E-02
AF086541	death associated protein-like 1	2.30E-03
AF087984	major facilitator superfamily domain containing 8	2.98E-02
AF088057	glycosylphosphatidylinositol anchored high density lipoprotein binding protein 1	1.86E-02
AF088062	uncharacterized LOC644873	1.16E-02
AF089897	PAP associated domain containing 5	3.86E-03
AF090102	poly(A) binding protein interacting protein 2B	3.97E-02
AF090906	Rho guanine nucleotide exchange factor (GEF) 12	3.02E-02
AF095289	pituitary tumor-transforming 3, pseudogene	8.28E-03
AF097645	integrator complex subunit 6	1.99E-02
AF104921	succinate-CoA ligase, alpha subunit	1.62E-02
AF111846	uncharacterized LOC28994	5.10E-03
AF111849	ELOVL fatty acid elongase 5	7.23E-03
AF112207	eukaryotic translation initiation factor 2B, subunit 4 delta, 67kDa	4.14E-02
AF112219	esterase D	3.67E-02
AF113701	ribosomal protein L22	7.71E-03
AF114263	chromosome 15 open reading frame 41	1.16E-02
AF116652	solute carrier family 16, member 10 (aromatic amino acid transporter)	7.78E-03
AF116682	ABRA C-terminal like	9.56E-03
AF117338	KAT8 regulatory NSL complex subunit 3	3.19E-02
AF118124	myeloid cell leukemia sequence 1 (BCL2-related)	6.01E-05
AF124366	family with sequence similarity 167, member A	3.68E-04
AF131778	discs, large (Drosophila) homolog-associated protein 3	2.76E-02
AF131790	SH3 and multiple ankyrin repeat domains 2	1.48E-02
AF131803	transmembrane protein 109	3.35E-02
AF131823	ankyrin 2, neuronal	3.29E-02
AF140710	ribosomal protein S6 kinase, 90kDa, polypeptide 2	2.14E-02
AF142063	oculomedin	1.34E-02
AF147314	family with sequence similarity 43, member A	3.80E-02
AF147421	ARHGAP5 antisense RNA 1 (head to head)	3.81E-02
AF149724	inhibitor of growth family, X-linked, pseudogene	4.96E-02
AF151109	mitochondrial ribosomal protein L36	1.06E-02
AF153330	solute carrier family 19 (thiamine transporter), member 2	1.90E-02

AF155103	ankyrin repeat domain 13A	2.36E-03
AF155108	coiled-coil domain containing 34	4.30E-02
AF155654	Morf4 family associated protein 1-like 1	3.78E-02
AF157323	F-box and leucine-rich repeat protein 5	1.13E-02
AF157562	elongation factor RNA polymerase II	2.14E-03
AF160477	poliovirus receptor-related 4	3.74E-02
AF161360	zinc finger, DHHC-type containing 21	7.18E-03
AF161383	coiled-coil domain containing 167	1.43E-02
AF161401	mitochondrial ribosomal protein L32	2.80E-02
AF161436	aminoadipate-semialdehyde dehydrogenase	3.26E-03
AF161437	glutamate-rich 1	1.65E-03
AF174487	BCL2-related ovarian killer	1.34E-02
AF174605	F-box protein 25	3.61E-02
AF176012	DnaJ (Hsp40) homolog, subfamily C, member 12	2.81E-02
AF177473	transient receptor potential cation channel, subfamily M, member 5	3.71E-02
AF178930	nucleotide-binding oligomerization domain containing 2	4.74E-02
AF179890	polymerase (DNA-directed), delta 4, accessory subunit	1.21E-02
AF181994	Fanconi anemia, complementation group F	3.45E-02
AF182069	prokineticin 2	3.91E-02
AF182416	NIF3 NGG1 interacting factor 3-like 1 (S. cerevisiae)	4.73E-02
AF182423	tRNA methyltransferase 11 homolog (S. cerevisiae)	1.29E-02
AF185573	myomesin 1	3.03E-02
AF192979	zinc finger protein 229	1.09E-02
AF195192	matrix metallopeptidase 27	2.89E-02
AF207547	large tumor suppressor kinase 2	8.04E-03
AF208232	interferon, alpha-inducible protein 27-like 2	3.70E-03
AF209930	chordin	1.83E-02
AF217190	DEAH (Asp-Glu-Ala-His) box polypeptide 36	4.73E-02
AF218008	major facilitator superfamily domain containing 12	2.36E-02
AF225422	MIF4G domain containing	4.08E-02
AF231023	cadherin, EGF LAG seven-pass G-type receptor 3	3.47E-02
AF234532	myosin X	9.68E-04
AF234654	placenta-specific 1	1.72E-02

AF235022	RAB38, member RAS oncogene family	1.55E-02
AF235049	inhibitor of Bruton agammaglobulinemia tyrosine kinase	4.13E-03
AF237952	retinol dehydrogenase 14 (all-trans/9-cis/11-cis)	4.26E-02
AF239156	peptide deformylase (mitochondrial)	3.18E-02
AF245505	matrix-remodelling associated 5	1.44E-02
AF248646	matrix metallopeptidase 26	4.75E-03
AF251025	zinc finger, FYVE domain containing 1	4.37E-03
AF264717	myotubularin related protein 4	2.32E-02
AF264750	lysine (K)-specific methyltransferase 2C	5.17E-03
AF264781	chromosome 11 open reading frame 24	1.74E-02
AF268872	eukaryotic elongation factor, selenocysteine-tRNA-specific	4.84E-02
AF274347	PR domain containing 7	2.64E-02
AF277719	hydroxy-delta-5-steroid dehydrogenase, 3 beta- and steroid delta-isomerase 7	4.04E-02
AF282887	heparanase 2	4.38E-02
AF288207	cysteinyl-tRNA synthetase	3.20E-02
AF289485	chromosome 12 open reading frame 10	1.46E-02
AF294629	ALX homeobox 4	4.24E-02
AF301463	SMAD specific E3 ubiquitin protein ligase 2	4.03E-03
AF302502	pellino E3 ubiquitin protein ligase family member 2	1.72E-02
AF305836	DIO3 opposite strand/antisense RNA (head to head)	1.13E-02
AF311862	lin-7 homolog B (C. elegans)	3.86E-02
AF311912	secreted frizzled-related protein 2	2.74E-02
AF317058	porcupine homolog (Drosophila)	2.06E-02
AF320294	ATP-binding cassette, sub-family G (WHITE), member 8	1.53E-02
AJ008151	mitogen-activated protein kinase kinase kinase 14	2.01E-03
AJ009817	Alport syndrome, mental retardation, midface hypoplasia and elliptocytosis chromosomal region gene	3.83E-02
AJ224166	matrin 3	5.49E-04
AJ227863	muscleblind-like splicing regulator 1	1.45E-03
AJ238899	neuronal guanine nucleotide exchange factor	4.02E-02
AJ249902	SPARC related modular calcium binding 2	5.76E-03
AJ249975	ankyrin repeat domain 2 (stretch responsive muscle)	2.69E-02
AJ252011	acid-sensing (proton-gated) ion channel family member 5	2.28E-02
AJ276429	SLAM family member 7	3.73E-02

AJ276510	uncharacterized protein, clone pT-Adv JuaX22	4.70E-02
AJ295148	O-sialoglycoprotein endopeptidase-like 1	3.48E-02
AK000002	ATP-binding cassette, sub-family C (CFTR/MRP), member 10	4.97E-02
AK000004	FYVE, RhoGEF and PH domain containing 3	1.69E-02
AK000049	charged multivesicular body protein 4C	1.00E-02
AK000102	zinc finger protein 280C	2.17E-02
AK000127	BCL2-like 14 (apoptosis facilitator)	3.31E-02
AK000263	R3H domain containing 4	4.45E-02
AK000569	zinc finger protein 30	3.04E-04
AK000643	ankyrin repeat and SOCS box containing 9	2.66E-02
AK000724	karyopherin alpha 6 (importin alpha 7)	4.41E-02
AK000851	TBC1 domain family, member 22A	3.39E-03
AK000995	BTB (POZ) domain containing 18	4.45E-02
AK001067	nuclear factor of activated T-cells 5, tonicity-responsive	6.76E-03
AK001069	UPF3 regulator of nonsense transcripts homolog A (yeast)	4.32E-04
AK001814	uncharacterized LOC100505876	2.98E-03
AK001822	arrestin domain containing 1	2.45E-02
AK001843	family with sequence similarity 126, member B	5.27E-03
AK001942	family with sequence similarity 49, member A	1.12E-02
AK002000	neuronal PAS domain protein 3	1.94E-02
AK002138	caspase recruitment domain family, member 14	4.65E-02
AK002209	uncharacterized LOC100506655	3.42E-02
AK021457	suppressor of Ty 20 homolog (S. cerevisiae)	1.61E-02
AK021510	phosphatidylinositol-4,5-bisphosphate 3-kinase, catalytic subunit alpha	2.33E-02
AK021541	sidekick cell adhesion molecule 2	4.06E-02
AK021543	DNM3 opposite strand/antisense RNA	3.95E-02
AK021552	mir-100-let-7a-2 cluster host gene (non-protein coding)	2.02E-02
AK021560	F-box and leucine-rich repeat protein 13	2.82E-02
AK021583	small nuclear ribonucleoprotein 200kDa (U5)	2.91E-02
AK021607	essential meiotic endonuclease 1 homolog 1 (S. pombe)	4.18E-02
AK021715	DEAD (Asp-Glu-Ala-Asp) box helicase 6	3.59E-04
AK021725	BMP2 inducible kinase	3.21E-02
AK021728	prickle homolog 1 (Drosophila)	2.62E-02

AK021762	Ras and Rab interactor 3	6.30E-03
AK021852	dachsous 1 (Drosophila)	2.96E-03
AK022003	TAF15 RNA polymerase II, TATA box binding protein (TBP)-associated factor, 68kDa	4.29E-02
AK022090	guanylate cyclase 1, soluble, alpha 2	1.24E-02
AK022200	dynactin 5 (p25)	1.18E-02
AK022212	gasdermin D	3.25E-03
AK022255	chromosome 8 open reading frame 60	2.78E-02
AK022290	SET and MYND domain containing 1	1.36E-02
AK022326	catenin (cadherin-associated protein), alpha 1, 102kDa	2.55E-02
AK022400	septin 4	2.85E-02
AK022401	tuberous sclerosis 2	4.31E-02
AK022412	dedicator of cytokinesis 6	9.76E-03
AK022437	FRMD6 antisense RNA 1	4.27E-02
AK022446	solute carrier family 39 (zinc transporter), member 14	1.16E-02
AK022478	SET binding factor 2	4.21E-02
AK022537	ribosome production factor 1 homolog (S. cerevisiae)	1.09E-02
AK022551	fibrosin	3.65E-02
AK022604	centrosomal protein 76kDa	3.57E-02
AK022613	Fanconi anemia, complementation group D2	1.75E-02
AK022638	fukutin related protein	2.92E-02
AK022711	MAP7 domain containing 3	2.93E-02
AK022810	MAP6 domain containing 1	6.02E-03
AK022811	cytochrome b5 domain containing 1	3.55E-02
AK022909	microcephalin 1	5.88E-03
AK022955	cell division cycle associated 7-like	3.54E-02
AK023071	salvador homolog 1 (Drosophila)	3.41E-02
AK023114	NAD kinase	1.71E-02
AK023185	cytochrome c oxidase assembly homolog 19 (S. cerevisiae)	4.82E-02
AK023220	alpha tubulin acetyltransferase 1	2.89E-02
AK023250	family with sequence similarity 204, member A	2.36E-02
AK023284	zinc finger, AN1-type domain 3	4.31E-02
AK023312	MAN1B1 antisense RNA 1 (head to head)	1.85E-03
AK023400	membrane-associated ring finger (C3HC4) 7, E3 ubiquitin protein ligase	2.59E-02

AK023404	cyclin B2	4.83E-02
AK023435	GTPase, very large interferon inducible pseudogene 1	1.22E-02
AK023557	core 1 synthase, glycoprotein-N-acetylgalactosamine 3-beta-galactosyltransferase, 1	3.06E-02
AK023589	U2 small nuclear RNA auxiliary factor 1	2.49E-02
AK023636	membrane-associated ring finger (C3HC4) 8, E3 ubiquitin protein ligase	1.19E-02
AK023641	SH3 domain and tetratricopeptide repeats 2	7.85E-03
AK023645	cyclin T2	2.77E-02
AK023754	hairy and enhancer of split 2 (Drosophila)	2.17E-02
AK023755	triggering receptor expressed on myeloid cells-like 2	1.38E-02
AK023769	zinc finger protein 552	1.55E-02
AK023803	ADP-ribosylation factor 1	1.62E-02
AK023897	retinoblastoma binding protein 9	4.31E-02
AK023901	zinc finger protein 407	2.47E-02
AK023911	IBA57, iron-sulfur cluster assembly homolog (S. cerevisiae)	3.66E-02
AK023916	DEP domain containing MTOR-interacting protein	1.53E-02
AK023971	chromosome 16 open reading frame 59	2.66E-03
AK024160	zinc finger protein 431	4.32E-02
AK024165	chromosome X open reading frame 36	4.88E-02
AK024177	long intergenic non-protein coding RNA 963	6.21E-03
AK024216	N(alpha)-acetyltransferase 60, NatF catalytic subunit	4.33E-02
AK024235	tigger transposable element derived 4	2.54E-02
AK024238	cadherin 6, type 2, K-cadherin (fetal kidney)	7.52E-03
AK024270	kelch-like family member 24	3.39E-02
AK024275	proline rich 5 like	3.31E-02
AK024282	synapse differentiation inducing 1	3.05E-02
AK024286	SAYSVFN motif domain containing 1	4.09E-02
AK024289	coiled-coil domain containing 24	3.42E-02
AK024327	RPTOR independent companion of MTOR, complex 2	9.10E-03
AK024341	ubiquitin specific peptidase 34	2.25E-03
AK024423	autophagy related 16-like 2 (S. cerevisiae)	3.73E-02
AK024462	sialic acid binding Ig-like lectin 1, sialoadhesin	1.99E-03
AK024484	WD repeat domain 5	3.43E-02
AK024489	protein phosphatase 1, regulatory subunit 3E	1.49E-02

AK024535	signal transducer and activator of transcription 3 (acute-phase response factor)	1.36E-03
AK024551	transmembrane protein 204	1.31E-02
AK024570	mitochondrial ribosomal protein L24	4.47E-02
AK024618	prostate transmembrane protein, androgen induced 1	2.14E-02
AK024632	transmembrane protein 185B	3.82E-02
AK024643	zinc finger protein 496	1.58E-02
AK024690	F-box and leucine-rich repeat protein 20	1.83E-03
AK024699	pancreatic progenitor cell differentiation and proliferation factor homolog (zebrafish)	3.86E-02
AK024714	kinesin light chain 1	4.99E-02
AK024738	peroxisome proliferator-activated receptor alpha	2.57E-02
AK024747	HAUS augmin-like complex, subunit 1	2.06E-03
AK024756	family with sequence similarity 118, member B	4.87E-04
AK024801	SET domain containing 6	2.25E-02
AK024843	dCTP pyrophosphatase 1	3.52E-02
AK024865	UDP-N-acetyl-alpha-D-galactosamine:polypeptide N-acetylgalactosaminyltransferase 12 (GalNAc-T12)	2.55E-02
AK024889	laminin, alpha 3	5.74E-03
AK024890	pyrin and HIN domain family, member 1	1.88E-02
AK024944	ELK4, ETS-domain protein (SRF accessory protein 1)	1.59E-02
AK024981	CREB regulated transcription coactivator 3	1.77E-02
AK025036	dual specificity phosphatase 18	4.27E-02
AK025092	spastic paraplegia 11 (autosomal recessive)	3.34E-02
AK025111	butyrophilin-like 8	9.44E-05
AK025265	fem-1 homolog c (C. elegans)	1.43E-03
AK025308	Gse1 coiled-coil protein	2.82E-02
AK025316	activating transcription factor 6	1.73E-03
AK025419	Spi-B transcription factor (Spi-1/PU.1 related)	3.56E-02
AK025489	GEN1 Holliday junction 5' flap endonuclease	6.38E-03
AK025520	acyl-CoA binding domain containing 3	3.88E-02
AK025562	chloride channel, voltage-sensitive 5	3.07E-02
AK025585	prolyl-tRNA synthetase 2, mitochondrial (putative)	2.40E-03
AK025592	5-azacytidine induced 2	2.47E-02
AK025598	chromosome 2 open reading frame 44	4.85E-02
AK025602	small integral membrane protein 7	2.77E-02

AK025615	branched chain amino-acid transaminase 1, cytosolic	2.35E-02
AK025695	transmembrane emp24 protein transport domain containing 8	9.54E-03
AK025758	nuclear factor of activated T-cells, cytoplasmic, calcineurin-dependent 2	7.30E-03
AK025798	VMA21 vacuolar H+-ATPase homolog (S. cerevisiae)	3.07E-02
AK025908	chromosome 12 open reading frame 65	1.32E-02
AK025965	biorientation of chromosomes in cell division 1-like 1	1.54E-02
AK025985	zinc finger and BTB domain containing 38	4.31E-02
AK026039	rogdi homolog (Drosophila)	1.97E-02
AK026157	zinc finger protein 335	2.80E-02
AK026196	dehydrogenase/reductase (SDR family) member 11	4.35E-02
AK026265	LON peptidase N-terminal domain and ring finger 3	1.60E-02
AK026266	fer-1-like 4 (C. elegans) pseudogene	4.12E-02
AK026277	bora, aurora kinase A activator	2.64E-04
AK026288	ATH1, acid trehalase-like 1 (yeast)	3.24E-02
AK026299	transcriptional adaptor 2B	1.93E-03
AK026309	calcium/calmodulin-dependent protein kinase II gamma	2.68E-02
AK026321	LGALS8 antisense RNA 1	1.78E-02
AK026366	zinc finger protein 677	3.22E-02
AK026373	heterogeneous nuclear ribonucleoprotein A2/B1	4.88E-02
AK026486	nucleic acid binding protein 1	1.17E-02
AK026496	EF-hand domain (C-terminal) containing 2	3.48E-04
AK026502	uncharacterized LOC100507637	3.85E-02
AK026518	myosin XIX	3.20E-02
AK026663	dedicator of cytokinesis 5	2.82E-02
AK026700	centrosomal protein 97kDa	3.57E-02
AK026709	FAT atypical cadherin 4	3.99E-02
AK026798	NACHT and WD repeat domain containing 1	1.45E-02
AK026841	solute carrier family 12 (potassium/chloride transporters), member 8	4.59E-02
AK026874	chromosome 1 open reading frame 54	2.13E-02
AK026898	forkhead box P1	2.43E-04
AK026942	arylsulfatase B	3.89E-02
AK026945	tribbles homolog 3 (Drosophila)	3.96E-02
AK026959	FOS-like antigen 2	3.24E-02

AK027094	asparaginyl-tRNA synthetase 2, mitochondrial (putative)	2.30E-03
AK027124	MICAL-like 2	3.88E-02
AK027180	myocyte enhancer factor 2D	2.10E-02
AK027190	pleckstrin homology domain containing, family S member 1	3.67E-03
AK027239	eukaryotic translation initiation factor 4E family member 2	2.18E-02
AL049382	chromosome 12 open reading frame 29	1.94E-02
AL049670	TIP41, TOR signaling pathway regulator-like (S. cerevisiae)	4.07E-02
AL049675	uncharacterized LOC100506963	3.41E-03
AL049925	pygopus homolog 1 (Drosophila)	8.62E-04
AL049943	family with sequence similarity 98, member A	2.02E-02
AL049955	NADH dehydrogenase (ubiquinone) complex I, assembly factor 3	3.44E-02
AL049963	solute carrier family 39 (zinc transporter), member 8	8.31E-03
AL049985	RAB22A, member RAS oncogene family	3.44E-02
AL050024	sclerostin domain containing 1	1.27E-02
AL050074	TBC1 domain family, member 30	3.85E-02
AL050078	post-GPI attachment to proteins 1	7.96E-05
AL050097	DKFZP586B0319 protein	4.65E-03
AL050143	sushi, nidogen and EGF-like domains 1	4.97E-02
AL050288	autophagy related 4B, cysteine peptidase	1.76E-02
AL050297	R3H domain and coiled-coil containing 1	2.00E-02
AL050298	ankyrin repeat domain 32	1.20E-02
AL050346	rhomboid domain containing 3	2.93E-02
AL050358	solute carrier family 26, member 10	1.83E-02
AL050370	kelch-like family member 35	1.03E-02
AL079274	homeobox A2	4.84E-02
AL079310	HMG box domain containing 4	3.69E-02
AL080062	methylmalonic aciduria (cobalamin deficiency) cblC type, with homocystinuria	1.69E-02
AL080066	serine palmitoyltransferase, small subunit A	1.13E-03
AL080092	paroxysmal nonkinesigenic dyskinesia	1.54E-02
AL080095	uncharacterized LOC100509635	3.65E-02
AL080111	NIMA-related kinase 7	1.92E-02
AL080113	DEAD (Asp-Glu-Ala-Asp) box helicase 17	4.35E-02
AL080133	spectrin repeat containing, nuclear envelope 2	6.75E-03

AL080191	uncharacterized LOC26102	3.06E-02
AL080200	long intergenic non-protein coding RNA 588	8.38E-03
AL109702	adaptor-related protein complex 3, sigma 2 subunit	3.43E-02
AL110139	family with sequence similarity 174, member B	8.81E-03
AL110161	helicase with zinc finger	1.76E-02
AL110199	adenomatosis polyposis coli down-regulated 1	3.93E-02
AL110226	ADAM metallopeptidase with thrombospondin type 1 motif, 7	4.36E-02
AL110235	cannabinoid receptor interacting protein 1	2.26E-03
AL110261	C1q and tumor necrosis factor related protein 5///membrane frizzled-related protein	2.32E-02
AL117439	chromosome 20 open reading frame 26	1.64E-02
AL117444	F-box protein 31	3.90E-02
AL117448	DENN/MADD domain containing 5A	1.43E-02
AL117478	G-protein signaling modulator 1	1.98E-02
AL117529	uncharacterized LOC100653005	1.88E-02
AL117565	cysteine-serine-rich nuclear protein 1	4.85E-02
AL117568	ubiquitin-conjugating enzyme E2K	3.30E-02
AL117580	uncharacterized LOC26077	1.91E-02
AL117595	Kruppel-like factor 6	2.09E-02
AL117663	transcription factor 3	2.85E-02
AL122047	ARV1 homolog (S. cerevisiae)	1.01E-03
AL122088	LysM, putative peptidoglycan-binding, domain containing 1	3.12E-03
AL133027	coiled-coil domain containing 136	4.02E-02
AL133031	ligand dependent nuclear receptor corepressor-like	4.17E-02
AL133035	filamin binding LIM protein 1	4.45E-02
AL133053	KAT8 regulatory NSL complex subunit 1-like	3.83E-02
AL133057	leucine-rich repeats and WD repeat domain containing 1	9.51E-03
AL133068	Mov10l1, Moloney leukemia virus 10-like 1, homolog (mouse)	7.22E-03
AL133069	F-box protein, helicase, 18	3.21E-02
AL133087	ankyrin repeat domain 44	2.73E-02
AL133094	PHD finger protein 10	7.39E-03
AL133097	interleukin 17 receptor D	1.81E-02
AL133101	centrosomal protein 85kDa-like	3.33E-02
AL133105	family with sequence similarity 20, member A	2.18E-02

AL133108	zinc finger homeobox 3	1.49E-02
AL133585	DEAD (Asp-Glu-Ala-Asp) box helicase 5	1.92E-02
AL137268	angel homolog 1 (Drosophila)	1.32E-02
AL137270	long intergenic non-protein coding RNA 939	3.58E-02
AL137290	aryl hydrocarbon receptor nuclear translocator	1.12E-03
AL137310	solute carrier family 4, sodium bicarbonate cotransporter, member 8	2.71E-02
AL137340	uncharacterized protein DKFZp761C1711	2.63E-02
AL137382	solute carrier family 22, member 31	2.92E-02
AL137385	heat shock transcription factor family member 5	2.53E-02
AL137430	uncharacterized LOC283070	5.09E-03
AL137437	solute carrier family 6, member 17	4.42E-02
AL137445	uncharacterized LOC401320	3.61E-02
AL137479	two pore segment channel 2	2.27E-02
AL137560	protein tyrosine phosphatase, receptor type, E	2.63E-02
AL137589	F-box and leucine-rich repeat protein 19	2.35E-02
AL137597	tumor protein p53 inducible nuclear protein 2	2.88E-03
AL137678	sel-1 suppressor of lin-12-like 2 (C. elegans)	1.32E-02
AL137679	exoribonuclease 1	1.32E-02
AL137709	STARD13 antisense RNA	2.16E-02
AL137722	von Willebrand factor A domain containing 1	2.65E-02
AL137761	family with sequence similarity 110, member C	3.35E-02
AL157421	SPAG5 antisense RNA 1	3.67E-03
AL157438	mitogen-activated protein kinase 1	3.41E-02
AL157442	glutamate receptor, ionotropic, N-methyl D-aspartate-associated protein 1 (glutamate binding)	4.38E-02
AL161992	MIT, microtubule interacting and transport, domain containing 1	1.77E-02
AL353944	runt-related transcription factor 2	1.38E-03
AL359055	metastasis associated in colon cancer 1	2.00E-02
AL359562	polycomb group ring finger 3	1.49E-02
AL359591	transmembrane protein 55A	7.15E-03
AL359943	uncharacterized LOC115110	3.15E-02
AL365370	zinc finger protein 77	7.70E-03
AL365410	myeloid/lymphoid or mixed-lineage leukemia (trithorax homolog, Drosophila); translocated to, 1	4.87E-02
AL390174	SLIT-ROBO Rho GTPase activating protein 3	4.16E-02

AL390181	uncharacterized LOC642852	1.42E-02
AY005822	acyl-CoA thioesterase 2	4.26E-02
AY009090	C-type lectin domain family 7, member A	6.76E-03
AY009151	centromere protein K	1.09E-02
AY009398	wingless-type MMTV integration site family, member 4	9.77E-03
AY010111	cadherin-related 23	1.55E-02
D13540	protein tyrosine phosphatase, non-receptor type 11	6.15E-03
D16892	heat shock protein 90kDa beta (Grp94), member 1	1.92E-02
D17130	H3 histone, family 3A, pseudogene 4///H3 histone, family 3A	3.83E-02
D17188	SENP3-EIF4A1 readthrough///small nucleolar RNA, H/ACA box 67///eukaryotic translation initiation fa	2.20E-02
D17200	KAT8 regulatory NSL complex subunit 1-like///ribulose-5-phosphate-3-epimerase	3.13E-02
D17216	transmembrane emp24 protein transport domain containing 4	7.98E-03
D25271	uncharacterized LOC100128651	1.20E-03
D26018	polymerase (DNA-directed), delta 3, accessory subunit	2.08E-03
D28476	thyroid hormone receptor interactor 12	7.28E-03
D29012	proteasome (prosome, macropain) subunit, beta type, 6	2.17E-02
D29958	exosome component 7	1.20E-02
D38438	postmeiotic segregation increased 2 pseudogene 4	4.62E-03
D42039	mesoderm development candidate 2	2.07E-02
D70833	zinc finger protein 208	3.69E-02
D86961	lipoma HMGIC fusion partner-like 2	4.54E-02
D86978	nucleoporin 205kDa	4.88E-02
D86980	tetratricopeptide repeat domain 9	9.58E-03
D86985	KIAA0232	1.50E-02
D87452	inositol hexakisphosphate kinase 1	1.84E-02
J02625	cytochrome P450, family 2, subfamily E, polypeptide 1	1.73E-02
J04205	Sjogren syndrome antigen B (autoantigen La)	1.43E-02
J05158	carboxypeptidase N, polypeptide 2	3.92E-02
J05200	ryanodine receptor 1 (skeletal)	1.02E-02
K01900	interferon, alpha 8	1.91E-02
K03200	antigen p97 (melanoma associated) identified by monoclonal antibodies 133.2 and 96.5	4.83E-02
L05500	adenylate cyclase 1 (brain)	1.98E-02
L10284	calnexin	1.63E-02

L11924	macrophage stimulating 1 (hepatocyte growth factor-like)	9.21E-03
L20686	son of sevenless homolog 2 (Drosophila)	4.34E-03
L21934	sterol O-acyltransferase 1	6.76E-03
L33813	alpha thalassemia/mental retardation syndrome X-linked	3.04E-02
L37198	SH3-domain binding protein 2	1.51E-02
L77561	DiGeorge syndrome critical region gene 11 (non-protein coding)	4.12E-02
M12807	CD4 molecule	2.57E-02
M14200	diazepam binding inhibitor (GABA receptor modulator, acyl-CoA binding protein)	1.62E-03
M17017	interleukin 8	6.89E-03
M23161	multiple coagulation factor deficiency 2	1.27E-02
M26663	kallikrein-related peptidase 3	8.21E-03
M29540	carcinoembryonic antigen-related cell adhesion molecule 5	1.79E-02
M34356	cAMP responsive element binding protein 1	3.30E-02
M58050	CD46 molecule, complement regulatory protein	1.33E-02
M60314	bone morphogenetic protein 5	2.11E-02
M82882	E74-like factor 1 (ets domain transcription factor)	3.72E-02
M95610	collagen, type IX, alpha 2	3.53E-02
NM_000016	acyl-CoA dehydrogenase, C-4 to C-12 straight chain	3.03E-03
NM_000027	aspartylglucosaminidase	4.75E-02
NM_000028	amylo-alpha-1, 6-glucosidase, 4-alpha-glucanotransferase	1.49E-02
NM_000035	aldolase B, fructose-bisphosphate	2.29E-02
NM_000040	apolipoprotein C-III	8.92E-03
NM_000042	apolipoprotein H (beta-2-glycoprotein I)	1.54E-02
NM_000054	arginine vasopressin receptor 2	4.81E-02
NM_000055	butyrylcholinesterase	1.63E-02
NM_000067	carbonic anhydrase II	1.41E-02
NM_000103	cytochrome P450, family 19, subfamily A, polypeptide 1	2.51E-02
NM_000104	cytochrome P450, family 1, subfamily B, polypeptide 1	3.46E-03
NM_000116	tafazzin	4.73E-02
NM_000119	erythrocyte membrane protein band 4.2	3.47E-03
NM_000127	exostosin glycosyltransferase 1	4.47E-02
NM_000142	fibroblast growth factor receptor 3	1.33E-02
NM_000144	frataxin	1.62E-02

NM_000168	GLI family zinc finger 3	1.74E-02
NM_000172	guanine nucleotide binding protein (G protein), alpha transducing activity polypeptide 1	2.56E-02
NM_000181	glucuronidase, beta	1.07E-03
NM_000199	N-sulfoglucosamine sulfohydrolase	1.30E-02
NM_000202	iduronate 2-sulfatase	2.52E-03
NM_000290	phosphoglycerate mutase 2 (muscle)	4.50E-02
NM_000295	serpin peptidase inhibitor, clade A (alpha-1 antiproteinase, antitrypsin), member 1	4.91E-02
NM_000299	plakophilin 1 (ectodermal dysplasia/skin fragility syndrome)	1.84E-02
NM_000305	paraoxonase 2	4.82E-02
NM_000311	prion protein	1.65E-02
NM_000313	protein S (alpha)	1.00E-02
NM_000320	quinoid dihydropteridine reductase	2.09E-02
NM_000328	retinitis pigmentosa GTPase regulator	4.71E-02
NM_000363	troponin I type 3 (cardiac)	2.71E-04
NM_000372	tyrosinase	5.24E-03
NM_000382	aldehyde dehydrogenase 3 family, member A2	2.80E-02
NM_000392	ATP-binding cassette, sub-family C (CFTR/MRP), member 2	3.83E-02
NM_000403	UDP-galactose-4-epimerase	2.95E-02
NM_000426	laminin, alpha 2	1.98E-03
NM_000437	platelet-activating factor acetylhydrolase 2, 40kDa	1.38E-02
NM_000454	superoxide dismutase 1, soluble	1.64E-02
NM_000456	sulfite oxidase	1.81E-02
NM_000482	apolipoprotein A-IV	3.64E-02
NM_000492	cystic fibrosis transmembrane conductance regulator (ATP-binding cassette sub-family C, member 7)	4.63E-03
NM_000502	eosinophil peroxidase	2.69E-03
NM_000509	fibrinogen gamma chain	4.80E-02
NM_000521	hexosaminidase B (beta polypeptide)	2.88E-02
NM_000522	homeobox A13	1.15E-02
NM_000550	tyrosinase-related protein 1	4.20E-02
NM_000562	complement component 8, alpha polypeptide	4.34E-03
NM_000565	interleukin 6 receptor	4.77E-02
NM_000627	latent transforming growth factor beta binding protein 1	2.78E-02
NM_000663	4-aminobutyrate aminotransferase	4.78E-02

NM_000690	aldehyde dehydrogenase 2 family (mitochondrial)	3.42E-02
NM_000696	aldehyde dehydrogenase 9 family, member A1	3.15E-02
NM_000700	annexin A1	2.03E-02
NM_000702	ATPase, Na+/K+ transporting, alpha 2 polypeptide	9.93E-03
NM_000707	arginine vasopressin receptor 1B	2.98E-02
NM_000712	biliverdin reductase A	1.17E-02
NM_000726	calcium channel, voltage-dependent, beta 4 subunit	3.30E-03
NM_000744	cholinergic receptor, nicotinic, alpha 4 (neuronal)	6.34E-03
NM_000761	cytochrome P450, family 1, subfamily A, polypeptide 2	2.94E-02
NM_000779	cytochrome P450, family 4, subfamily B, polypeptide 1	2.73E-03
NM_000788	deoxycytidine kinase	4.36E-03
NM_000814	gamma-aminobutyric acid (GABA) A receptor, beta 3	2.18E-02
NM_000815	gamma-aminobutyric acid (GABA) A receptor, delta	3.43E-02
NM_000821	gamma-glutamyl carboxylase	9.88E-03
NM_000830	glutamate receptor, ionotropic, kainate 1	2.79E-02
NM_000833	glutamate receptor, ionotropic, N-methyl D-aspartate 2A	1.75E-02
NM_000849	glutathione S-transferase mu 3 (brain)	4.73E-02
NM_000873	intercellular adhesion molecule 2	8.15E-03
NM_000895	leukotriene A4 hydrolase	7.72E-03
NM_000909	neuropeptide Y receptor Y1	3.44E-02
NM_000935	procollagen-lysine, 2-oxoglutarate 5-dioxygenase 2	3.69E-02
NM_000955	prostaglandin E receptor 1 (subtype EP1), 42kDa	2.86E-02
NM_000963	prostaglandin-endoperoxide synthase 2 (prostaglandin G/H synthase and cyclooxygenase)	8.55E-04
NM_000975	ribosomal protein L11	2.23E-02
NM_000979	ribosomal protein L18	4.62E-02
NM_001001	ribosomal protein L36a-like	2.34E-02
NM_001010	ribosomal protein S6	5.27E-03
NM_001011	ribosomal protein S7	2.22E-02
NM_001022	ribosomal protein S19	5.47E-03
NM_001024	ribosomal protein S21	3.96E-02
NM_001033	ribonucleotide reductase M1	1.93E-02
NM_001044	solute carrier family 6 (neurotransmitter transporter, dopamine), member 3	2.00E-02
NM_001045	solute carrier family 6 (neurotransmitter transporter, serotonin), member 4	1.67E-02

NM_001051	somatostatin receptor 3	4.98E-02
NM_001053	somatostatin receptor 5	4.17E-02
NM_001055	sulfotransferase family, cytosolic, 1A, phenol-preferring, member 1	1.30E-02
NM_001056	sulfotransferase family, cytosolic, 1C, member 2	1.02E-02
NM_001065	tumor necrosis factor receptor superfamily, member 1A	3.78E-02
NM_001085	serpin peptidase inhibitor, clade A (alpha-1 antiproteinase, antitrypsin), member 3	8.08E-03
NM_001099	acid phosphatase, prostate	4.96E-03
NM_001107	acylphosphatase 1, erythrocyte (common) type	3.47E-02
NM_001111	adenosine deaminase, RNA-specific	1.01E-02
NM_001117	adenylate cyclase activating polypeptide 1 (pituitary)	8.99E-03
NM_001120	major facilitator superfamily domain containing 10	4.81E-02
NM_001122	perilipin 2	4.91E-02
NM_001152	solute carrier family 25 (mitochondrial carrier; adenine nucleotide translocator), member 5	5.55E-03
NM_001172	arginase 2	2.23E-02
NM_001189	NK3 homeobox 2	2.37E-02
NM_001200	bone morphogenetic protein 2	2.50E-02
NM_001216	carbonic anhydrase IX	5.79E-03
NM_001225	caspase 4, apoptosis-related cysteine peptidase	4.98E-04
NM_001228	caspase 8, apoptosis-related cysteine peptidase	8.76E-03
NM_001248	ectonucleoside triphosphate diphosphohydrolase 3	1.32E-02
NM_001258	cyclin-dependent kinase 3	2.25E-03
NM_001261	cyclin-dependent kinase 9	3.91E-02
NM_001262	cyclin-dependent kinase inhibitor 2C (p18, inhibits CDK4)	1.52E-03
NM_001274	checkpoint kinase 1	2.92E-02
NM_001276	chitinase 3-like 1 (cartilage glycoprotein-39)	4.37E-07
NM_001281	tubulin folding cofactor B	5.08E-03
NM_001282	adaptor-related protein complex 2, beta 1 subunit	5.10E-03
NM_001288	chloride intracellular channel 1	4.19E-03
NM_001321	cysteine and glycine-rich protein 2	9.84E-03
NM_001326	cleavage stimulation factor, 3' pre-RNA, subunit 3, 77kDa	2.13E-02
NM_001346	diacylglycerol kinase, gamma 90kDa	2.12E-02
NM_001350	death-domain associated protein	3.33E-02
NM_001353	aldo-keto reductase family 1, member C1	2.73E-02

NM_001365	discs, large homolog 4 (Drosophila)	1.03E-02
NM_001397	endothelin converting enzyme 1	3.34E-03
NM_001424	epithelial membrane protein 2	1.60E-02
NM_001427	engrailed homeobox 2	8.07E-03
NM_001432	epiregulin	4.19E-02
NM_001438	estrogen-related receptor gamma	1.36E-02
NM_001440	exostosin-like glycosyltransferase 3	3.68E-02
NM_001455	forkhead box O3	6.33E-03
NM_001463	frizzled-related protein	4.08E-02
NM_001483	glioblastoma amplified sequence	2.01E-02
NM_001487	biogenesis of lysosomal organelles complex-1, subunit 1	1.50E-02
NM_001495	GDNF family receptor alpha 2	4.19E-02
NM_001500	GDP-mannose 4,6-dehydratase	4.16E-02
NM_001503	glycosylphosphatidylinositol specific phospholipase D1	5.03E-03
NM_001505	G protein-coupled estrogen receptor 1	3.22E-02
NM_001513	glutathione S-transferase zeta 1	1.36E-03
NM_001516	general transcription factor IIH, polypeptide 3, 34kDa	3.16E-02
NM_001533	heterogeneous nuclear ribonucleoprotein L	3.08E-02
NM_001537	heat shock factor binding protein 1	4.31E-02
NM_001545	immature colon carcinoma transcript 1	1.69E-02
NM_001550	interferon-related developmental regulator 1	6.38E-03
NM_001553	insulin-like growth factor binding protein 7	7.30E-04
NM_001561	tumor necrosis factor receptor superfamily, member 9	2.45E-02
NM_001570	interleukin-1 receptor-associated kinase 2	3.02E-02
NM_001586	testis expressed 28	4.06E-02
NM_001619	adrenergic, beta, receptor kinase 1	3.05E-02
NM_001645	apolipoprotein C-I	7.62E-03
NM_001651	aquaporin 5	4.24E-02
NM_001677	ATPase, Na+/K+ transporting, beta 1 polypeptide	4.19E-02
NM_001696	ATPase, H+ transporting, lysosomal 31kDa, V1 subunit E1	2.80E-02
NM_001703	brain-specific angiogenesis inhibitor 2	4.97E-02
NM_001719	bone morphogenetic protein 7	2.23E-02
NM_001731	B-cell translocation gene 1, anti-proliferative	1.06E-02

NM_001734	complement component 1, s subcomponent	3.83E-03
NM_001735	complement component 5	3.33E-03
NM_001806	CCAAT/enhancer binding protein (C/EBP), gamma	6.88E-03
NM_001814	cathepsin C	1.04E-04
NM_001815	carcinoembryonic antigen-related cell adhesion molecule 3	4.52E-02
NM_001827	CDC28 protein kinase regulatory subunit 2	3.92E-02
NM_001830	chloride channel, voltage-sensitive 4	2.07E-02
NM_001862	cytochrome c oxidase subunit Vb	1.46E-02
NM_001863	cytochrome c oxidase subunit VIb polypeptide 1 (ubiquitous)	2.61E-02
NM_001864	cytochrome c oxidase subunit VIIa polypeptide 1 (muscle)	2.40E-02
NM_001887	crystallin, beta B1	1.18E-02
NM_001896	casein kinase 2, alpha prime polypeptide	2.31E-02
NM_001912	cathepsin L1	1.62E-02
NM_001934	distal-less homeobox 4	1.59E-02
NM_001955	endothelin 1	4.44E-02
NM_001958	eukaryotic translation elongation factor 1 alpha 2	4.02E-03
NM_001966	enoyl-CoA, hydratase/3-hydroxyacyl CoA dehydrogenase	1.38E-03
NM_001978	dematin actin binding protein	1.70E-02
NM_001985	electron-transfer-flavoprotein, beta polypeptide	1.35E-02
NM_001995	acyl-CoA synthetase long-chain family member 1	5.56E-03
NM_002006	fibroblast growth factor 2 (basic)	4.71E-05
NM_002009	fibroblast growth factor 7	4.36E-02
NM_002013	FK506 binding protein 3, 25kDa	4.52E-04
NM_002014	FK506 binding protein 4, 59kDa	4.94E-02
NM_002015	forkhead box O1	4.45E-02
NM_002037	FYN oncogene related to SRC, FGR, YES	2.94E-02
NM_002047	glycyl-tRNA synthetase	2.70E-03
NM_002079	glutamic-oxaloacetic transaminase 1, soluble	4.81E-02
NM_002105	H2A histone family, member X	4.05E-02
NM_002107	H3 histone, family 3A	6.98E-03
NM_002109	histidyl-tRNA synthetase	1.59E-03
NM_002114	human immunodeficiency virus type I enhancer binding protein 1	1.73E-02
NM_002128	high mobility group box 1	3.56E-02

NM_002130	3-hydroxy-3-methylglutaryl-CoA synthase 1 (soluble)	9.83E-03
NM_002144	homeobox B1	3.71E-02
NM_002150	4-hydroxyphenylpyruvate dioxygenase	7.17E-03
NM_002151	hepsin	2.08E-03
NM_002205	integrin, alpha 5 (fibronectin receptor, alpha polypeptide)	2.17E-03
NM_002206	integrin, alpha 7	5.31E-04
NM_002208	integrin, alpha E (antigen CD103, human mucosal lymphocyte antigen 1; alpha polypeptide)	1.16E-02
NM_002209	integrin, alpha L (antigen CD11A (p180), lymphocyte function-associated antigen 1; alpha polypeptide)	2.60E-02
NM_002212	eukaryotic translation initiation factor 6	9.68E-03
NM_002216	inter-alpha-trypsin inhibitor heavy chain 2	4.75E-02
NM_002247	potassium large conductance calcium-activated channel, subfamily M, alpha member 1	2.84E-03
NM_002262	killer cell lectin-like receptor subfamily D, member 1	1.94E-02
NM_002264	karyopherin alpha 1 (importin alpha 5)	9.97E-03
NM_002276	keratin 19	3.60E-02
NM_002287	leukocyte-associated immunoglobulin-like receptor 1	1.39E-04
NM_002288	leukocyte-associated immunoglobulin-like receptor 2	6.57E-03
NM_002296	lamin B receptor	7.34E-03
NM_002312	ligase IV, DNA, ATP-dependent	2.77E-02
NM_002314	LIM domain kinase 1	2.19E-02
NM_002319	leucine-rich repeats and calponin homology (CH) domain containing 4	1.66E-02
NM_002333	low density lipoprotein receptor-related protein 3	2.27E-02
NM_002337	low density lipoprotein receptor-related protein associated protein 1	3.25E-04
NM_002356	myristoylated alanine-rich protein kinase C substrate	3.01E-04
NM_002357	MAX dimerization protein 1	1.81E-02
NM_002358	MAD2 mitotic arrest deficient-like 1 (yeast)	3.05E-02
NM_002373	microtubule-associated protein 1A	2.14E-02
NM_002413	microsomal glutathione S-transferase 2	2.21E-02
NM_002435	mannose phosphate isomerase	2.37E-02
NM_002444	moesin	2.08E-02
NM_002446	mitogen-activated protein kinase kinase kinase 10	2.89E-02
NM_002450	metallothionein 1L (gene/pseudogene)	1.02E-03
NM_002452	nudix (nucleoside diphosphate linked moiety X)-type motif 1	1.38E-02
NM_002454	5-methyltetrahydrofolate-homocysteine methyltransferase reductase	3.72E-02

NM_002461	mevalonate (diphospho) decarboxylase	4.13E-03
NM_002488	NADH dehydrogenase (ubiquinone) 1 alpha subcomplex, 2, 8kDa	1.33E-02
NM_002489	NADH dehydrogenase (ubiquinone) 1 alpha subcomplex, 4, 9kDa	1.33E-02
NM_002490	NADH dehydrogenase (ubiquinone) 1 alpha subcomplex, 6, 14kDa	1.60E-02
NM_002495	NADH dehydrogenase (ubiquinone) Fe-S protein 4, 18kDa (NADH-coenzyme Q reductase)	2.99E-02
NM_002503	nuclear factor of kappa light polypeptide gene enhancer in B-cells inhibitor, beta	3.60E-02
NM_002514	nephroblastoma overexpressed	4.92E-03
NM_002537	ornithine decarboxylase antizyme 2	4.30E-02
NM_002546	tumor necrosis factor receptor superfamily, member 11b	4.60E-02
NM_002553	origin recognition complex, subunit 5	6.84E-03
NM_002557	oviductal glycoprotein 1, 120kDa	4.73E-03
NM_002567	phosphatidylethanolamine binding protein 1	3.98E-02
NM_002575	serpin peptidase inhibitor, clade B (ovalbumin), member 2	7.38E-03
NM_002584	paired box 7	1.28E-02
NM_002587	protocadherin 1	2.71E-02
NM_002590	protocadherin 8	1.97E-02
NM_002592	proliferating cell nuclear antigen	6.24E-03
NM_002594	proprotein convertase subtilisin/kexin type 2	1.02E-02
NM_002600	phosphodiesterase 4B, cAMP-specific	2.91E-02
NM_002615	serpin peptidase inhibitor, clade F (alpha-2 antiplasmin, pigment epithelium derived factor), member [illegible]	4.94E-02
NM_002625	6-phosphofructo-2-kinase/fructose-2,6-biphosphatase 1	2.07E-02
NM_002628	profilin 2	1.58E-02
NM_002630	progastricsin (pepsinogen C)	2.07E-02
NM_002643	phosphatidylinositol glycan anchor biosynthesis, class F	1.87E-02
NM_002666	perilipin 1	3.62E-02
NM_002695	polymerase (RNA) II (DNA directed) polypeptide E, 25kDa	6.10E-03
NM_002696	polymerase (RNA) II (DNA directed) polypeptide G	7.15E-03
NM_002714	protein phosphatase 1, regulatory subunit 10	1.71E-02
NM_002729	hematopoietically expressed homeobox	1.16E-02
NM_002737	protein kinase C, alpha	4.12E-03
NM_002749	mitogen-activated protein kinase 7	3.25E-02
NM_002769	protease, serine, 1 (trypsin 1)	2.32E-02
NM_002770	protease, serine, 2 (trypsin 2)	1.03E-02

NM_002771	protease, serine, 3	4.87E-02
NM_002786	proteasome (prosome, macropain) subunit, alpha type, 1	1.44E-02
NM_002789	proteasome (prosome, macropain) subunit, alpha type, 4	2.31E-02
NM_002790	proteasome (prosome, macropain) subunit, alpha type, 5	8.64E-03
NM_002794	proteasome (prosome, macropain) subunit, beta type, 2	3.71E-02
NM_002799	proteasome (prosome, macropain) subunit, beta type, 7	5.35E-03
NM_002801	proteasome (prosome, macropain) subunit, beta type, 10	2.83E-03
NM_002803	proteasome (prosome, macropain) 26S subunit, ATPase, 2	3.63E-02
NM_002807	proteasome (prosome, macropain) 26S subunit, non-ATPase, 1	1.68E-02
NM_002826	quiescin Q6 sulfhydryl oxidase 1	4.54E-02
NM_002828	protein tyrosine phosphatase, non-receptor type 2	3.81E-02
NM_002829	protein tyrosine phosphatase, non-receptor type 3	2.62E-02
NM_002831	protein tyrosine phosphatase, non-receptor type 6	2.37E-02
NM_002836	protein tyrosine phosphatase, receptor type, A	2.12E-02
NM_002837	protein tyrosine phosphatase, receptor type, B	5.16E-03
NM_002843	protein tyrosine phosphatase, receptor type, J	3.02E-02
NM_002853	RAD1 homolog (S. pombe)	6.73E-04
NM_002859	paxillin	4.79E-03
NM_002865	RAB2A, member RAS oncogene family	3.98E-02
NM_002878	RAD51 homolog D (S. cerevisiae)	7.75E-03
NM_002880	v-raf-1 murine leukemia viral oncogene homolog 1	7.02E-04
NM_002905	retinol dehydrogenase 5 (11-cis/9-cis)	3.57E-02
NM_002922	regulator of G-protein signaling 1	2.29E-02
NM_002933	ribonuclease, RNase A family, 1 (pancreatic)	5.59E-03
NM_002979	sterol carrier protein 2	6.48E-03
NM_002980	secretin receptor	3.89E-02
NM_002989	chemokine (C-C motif) ligand 21	4.64E-02
NM_003003	SEC14-like 1 (S. cerevisiae)	1.11E-02
NM_003033	ST3 beta-galactoside alpha-2,3-sialyltransferase 1	6.97E-03
NM_003034	ST8 alpha-N-acetyl-neuraminide alpha-2,8-sialyltransferase 1	5.18E-03
NM_003035	SCL/TAL1 interrupting locus	1.61E-02
NM_003057	solute carrier family 22 (organic cation transporter), member 1	2.94E-02
NM_003060	solute carrier family 22 (organic cation/carnitine transporter), member 5	3.48E-03

NM_003064	secretory leukocyte peptidase inhibitor	2.15E-02
NM_003075	SWI/SNF related, matrix associated, actin dependent regulator of chromatin, subfamily c, member 2	9.11E-03
NM_003093	small nuclear ribonucleoprotein polypeptide C	5.88E-03
NM_003096	small nuclear ribonucleoprotein polypeptide G	3.36E-02
NM_003118	secreted protein, acidic, cysteine-rich (osteonectin)	4.50E-02
NM_003131	serum response factor (c-fos serum response element-binding transcription factor)	3.59E-02
NM_003133	signal recognition particle 9kDa	8.30E-03
NM_003134	signal recognition particle 14kDa (homologous Alu RNA binding protein)	4.43E-02
NM_003143	single-stranded DNA binding protein 1, mitochondrial	1.27E-02
NM_003152	signal transducer and activator of transcription 5A	1.92E-02
NM_003153	signal transducer and activator of transcription 6, interleukin-4 induced	3.96E-02
NM_003161	ribosomal protein S6 kinase, 70kDa, polypeptide 1	1.27E-02
NM_003205	transcription factor 12	3.26E-02
NM_003211	thymine-DNA glycosylase	3.87E-02
NM_003234	transferrin receptor (p90, CD71)	2.14E-02
NM_003242	transforming growth factor, beta receptor II (70/80kDa)	4.48E-02
NM_003248	thrombospondin 4	1.77E-02
NM_003270	tetraspanin 6	1.08E-02
NM_003277	claudin 5	4.29E-03
NM_003314	tetratricopeptide repeat domain 1	2.37E-02
NM_003331	tyrosine kinase 2	2.35E-02
NM_003340	ubiquitin-conjugating enzyme E2D 3	1.00E-02
NM_003366	ubiquinol-cytochrome c reductase core protein II	1.53E-02
NM_003384	vaccinia related kinase 1	7.20E-05
NM_003394	wingless-type MMTV integration site family, member 10B	4.91E-02
NM_003406	tyrosine 3-monooxygenase/tryptophan 5-monooxygenase activation protein, zeta polypeptide	4.59E-02
NM_003407	ZFP36 ring finger protein	1.43E-02
NM_003456	zinc finger protein 205	1.38E-02
NM_003467	chemokine (C-X-C motif) receptor 4	1.20E-02
NM_003470	ubiquitin specific peptidase 7 (herpes virus-associated)	1.41E-02
NM_003474	ADAM metallopeptidase domain 12	4.55E-02
NM_003477	pyruvate dehydrogenase complex, component X	8.68E-03
NM_003478	cullin 5	2.04E-02

NM_003500	acyl-CoA oxidase 2, branched chain	4.66E-02
NM_003501	acyl-CoA oxidase 3, pristanoyl	3.52E-02
NM_003520	histone cluster 1, H2bn	7.90E-04
NM_003544	histone cluster 1, H4b	4.14E-02
NM_003566	early endosome antigen 1	2.27E-02
NM_003581	NCK adaptor protein 2	1.23E-03
NM_003599	suppressor of Ty 3 homolog (S. cerevisiae)	6.66E-03
NM_003603	sorbin and SH3 domain containing 2	3.76E-02
NM_003608	G protein-coupled receptor 65	3.72E-03
NM_003626	protein tyrosine phosphatase, receptor type, f polypeptide (PTPRF), interacting protein (liprin), alpha 1	5.34E-03
NM_003648	diacylglycerol kinase, delta 130kDa	4.09E-03
NM_003721	regulatory factor X-associated ankyrin-containing protein	5.19E-03
NM_003749	insulin receptor substrate 2	4.96E-02
NM_003770	keratin 37	1.86E-02
NM_003776	mitochondrial ribosomal protein L40	1.92E-02
NM_003796	URI1, prefoldin-like chaperone	3.39E-02
NM_003804	receptor (TNFRSF)-interacting serine-threonine kinase 1	2.28E-02
NM_003854	interleukin 1 receptor-like 2	8.02E-03
NM_003887	ArfGAP with SH3 domain, ankyrin repeat and PH domain 2	2.51E-02
NM_003891	protein Z, vitamin K-dependent plasma glycoprotein	2.65E-02
NM_003897	immediate early response 3	2.12E-02
NM_003905	NEDD8 activating enzyme E1 subunit 1	1.74E-02
NM_003908	eukaryotic translation initiation factor 2, subunit 2 beta, 38kDa	6.25E-03
NM_003909	copine III	2.42E-02
NM_003940	ubiquitin specific peptidase 13 (isopeptidase T-3)	4.35E-03
NM_004039	annexin A2	3.96E-02
NM_004045	antioxidant 1 copper chaperone	3.13E-05
NM_004046	ATP synthase, H+ transporting, mitochondrial F1 complex, alpha subunit 1, cardiac muscle	9.11E-03
NM_004048	beta-2-microglobulin	4.84E-02
NM_004066	centrin, EF-hand protein, 1	4.05E-02
NM_004071	CDC-like kinase 1	1.72E-02
NM_004126	guanine nucleotide binding protein (G protein), gamma 11	8.94E-03
NM_004128	general transcription factor IIF, polypeptide 2, 30kDa	8.38E-03

NM_004130	glycogenin 1	3.36E-02
NM_004146	NADH dehydrogenase (ubiquinone) 1 beta subcomplex, 7, 18kDa	4.20E-02
NM_004148	ninjurin 1	1.85E-02
NM_004152	ornithine decarboxylase antizyme 1	3.14E-02
NM_004177	syntaxin 3	2.96E-02
NM_004190	lipase, gastric	1.87E-02
NM_004207	solute carrier family 16, member 3 (monocarboxylic acid transporter 4)	3.70E-02
NM_004226	serine/threonine kinase 17b	7.83E-03
NM_004236	COP9 signalosome subunit 2	4.30E-02
NM_004247	elongation factor Tu GTP binding domain containing 2	4.26E-02
NM_004251	RAB9A, member RAS oncogene family	1.53E-02
NM_004261	15 kDa selenoprotein	4.99E-02
NM_004264	mediator complex subunit 21	2.61E-02
NM_004270	mediator complex subunit 7	2.26E-03
NM_004279	peptidase (mitochondrial processing) beta	1.84E-02
NM_004280	eukaryotic translation elongation factor 1 epsilon 1	3.96E-02
NM_004284	chromodomain helicase DNA binding protein 1-like	3.49E-02
NM_004288	cytohesin 1 interacting protein	4.43E-02
NM_004293	guanine deaminase	2.18E-02
NM_004311	ADP-ribosylation factor-like 3	3.48E-03
NM_004318	aspartate beta-hydroxylase	3.45E-02
NM_004331	BCL2/adenovirus E1B 19kDa interacting protein 3-like	1.47E-07
NM_004337	oxidative stress induced growth inhibitor family member 2	1.07E-04
NM_004362	calmegin	2.33E-03
NM_004366	chloride channel, voltage-sensitive 2	3.64E-02
NM_004403	deafness, autosomal dominant 5	6.06E-03
NM_004415	desmoplakin	6.95E-03
NM_004417	dual specificity phosphatase 1	2.14E-02
NM_004430	early growth response 3	3.75E-02
NM_004444	EPH receptor B4	1.07E-02
NM_004451	estrogen-related receptor alpha	4.99E-03
NM_004453	electron-transferring-flavoprotein dehydrogenase	1.39E-02
NM_004470	FK506 binding protein 2, 13kDa	5.25E-04

NM_004472	forkhead box D1	4.16E-02
NM_004483	glycine cleavage system protein H (aminomethyl carrier)	4.68E-02
NM_004491	Rho GTPase activating protein 35	2.27E-02
NM_004519	potassium voltage-gated channel, KQT-like subfamily, member 3	1.17E-02
NM_004541	NADH dehydrogenase (ubiquinone) 1 alpha subcomplex, 1, 7.5kDa	1.54E-02
NM_004542	NADH dehydrogenase (ubiquinone) 1 alpha subcomplex, 3, 9kDa	1.67E-02
NM_004548	NADH dehydrogenase (ubiquinone) 1 beta subcomplex, 10, 22kDa	2.73E-02
NM_004549	NADH dehydrogenase (ubiquinone) 1, subcomplex unknown, 2, 14.5kDa	1.16E-04
NM_004552	NADH dehydrogenase (ubiquinone) Fe-S protein 5, 15kDa (NADH-coenzyme Q reductase)	8.32E-03
NM_004553	NADH dehydrogenase (ubiquinone) Fe-S protein 6, 13kDa (NADH-coenzyme Q reductase)	1.54E-02
NM_004569	phosphatidylinositol glycan anchor biosynthesis, class H	3.94E-03
NM_004587	ribosome binding protein 1	1.21E-02
NM_004634	bromodomain and PHD finger containing, 1	2.99E-02
NM_004661	cell division cycle 23	4.48E-02
NM_004672	mitogen-activated protein kinase kinase kinase 6	7.99E-03
NM_004676	PTPN13-like, Y-linked	3.83E-02
NM_004689	metastasis associated 1	2.70E-02
NM_004693	keratin 75	2.93E-02
NM_004717	diacylglycerol kinase, iota	1.13E-02
NM_004747	discs, large homolog 5 (Drosophila)	5.81E-03
NM_004755	ribosomal protein S6 kinase, 90kDa, polypeptide 5	1.41E-02
NM_004757	aminoacyl tRNA synthetase complex-interacting multifunctional protein 1	6.02E-03
NM_004763	integrin beta 1 binding protein 1	2.44E-03
NM_004765	B-cell CLL/lymphoma 7C	3.71E-03
NM_004774	mediator complex subunit 1	2.82E-02
NM_004776	UDP-Gal:betaGlcNAc beta 1,4- galactosyltransferase, polypeptide 5	3.69E-02
NM_004799	zinc finger, FYVE domain containing 9	4.70E-02
NM_004836	eukaryotic translation initiation factor 2-alpha kinase 3	3.45E-02
NM_004850	Rho-associated, coiled-coil containing protein kinase 2	2.33E-03
NM_004862	lipopolysaccharide-induced TNF factor	3.84E-03
NM_004874	BCL2-associated athanogene 4	3.82E-02
NM_004879	etoposide induced 2.4	2.93E-03
NM_004889	ATP synthase, H+ transporting, mitochondrial Fo complex, subunit F2	2.28E-02

NM_004890	sperm associated antigen 7	1.43E-02
NM_004891	mitochondrial ribosomal protein L33	4.90E-03
NM_004894	chromosome 14 open reading frame 2	9.01E-03
NM_004895	NLR family, pyrin domain containing 3	2.20E-03
NM_004913	VPS9 domain containing 1	3.63E-03
NM_004941	DEAH (Asp-Glu-Ala-His) box polypeptide 8	4.32E-03
NM_004948	desmocollin 1	2.72E-02
NM_004959	nuclear receptor subfamily 5, group A, member 1	1.33E-03
NM_004960	fused in sarcoma	1.71E-03
NM_004966	heterogeneous nuclear ribonucleoprotein F	2.55E-02
NM_004975	potassium voltage-gated channel, Shab-related subfamily, member 1	5.01E-03
NM_004985	v-Ki-ras2 Kirsten rat sarcoma viral oncogene homolog	8.83E-03
NM_004992	methyl CpG binding protein 2 (Rett syndrome)	9.57E-03
NM_004996	ATP-binding cassette, sub-family C (CFTR/MRP), member 1	4.41E-02
NM_005001	NADH dehydrogenase (ubiquinone) 1 alpha subcomplex, 7, 14.5kDa	3.95E-02
NM_005002	NADH dehydrogenase (ubiquinone) 1 alpha subcomplex, 9, 39kDa	3.84E-04
NM_005004	NADH dehydrogenase (ubiquinone) 1 beta subcomplex, 8, 19kDa	3.11E-02
NM_005005	NADH dehydrogenase (ubiquinone) 1 beta subcomplex, 9, 22kDa	1.87E-02
NM_005020	phosphodiesterase 1C, calmodulin-dependent 70kDa	5.75E-03
NM_005034	polymerase (RNA) II (DNA directed) polypeptide K, 7.0kDa	9.54E-03
NM_005044	protein kinase, X-linked	1.80E-02
NM_005060	RAR-related orphan receptor C	1.14E-02
NM_005080	X-box binding protein 1	1.25E-02
NM_005096	zinc finger, MYM-type 3	3.62E-02
NM_005098	musculin	1.71E-02
NM_005103	fasciculation and elongation protein zeta 1 (zygin I)	2.32E-02
NM_005134	protein phosphatase 4, regulatory subunit 1	2.09E-02
NM_005145	guanine nucleotide binding protein (G protein), gamma 7	2.43E-04
NM_005151	ubiquitin specific peptidase 14 (tRNA-guanine transglycosylase)	1.31E-02
NM_005156	polypyrimidine tract binding protein 3	1.21E-03
NM_005163	v-akt murine thymoma viral oncogene homolog 1	1.53E-02
NM_005165	aldolase C, fructose-bisphosphate	1.69E-02
NM_005174	ATP synthase, H+ transporting, mitochondrial F1 complex, gamma polypeptide 1	4.57E-04

NM_005182	carbonic anhydrase VII	3.36E-02
NM_005188	Cbl proto-oncogene, E3 ubiquitin protein ligase	2.86E-03
NM_005192	cyclin-dependent kinase inhibitor 3	2.19E-03
NM_005211	colony stimulating factor 1 receptor	7.43E-03
NM_005231	cortactin	3.57E-02
NM_005239	v-ets erythroblastosis virus E26 oncogene homolog 2 (avian)	4.83E-02
NM_005254	GA binding protein transcription factor, beta subunit 1	7.47E-04
NM_005284	G protein-coupled receptor 6	3.18E-02
NM_005288	G protein-coupled receptor 12	1.05E-02
NM_005293	G protein-coupled receptor 20	3.24E-02
NM_005295	G protein-coupled receptor 22	4.54E-02
NM_005301	G protein-coupled receptor 35	2.55E-02
NM_005303	free fatty acid receptor 1	4.41E-02
NM_005312	Rap guanine nucleotide exchange factor (GEF) 1	2.31E-05
NM_005323	histone cluster 1, H1t	1.25E-02
NM_005324	H3 histone, family 3B (H3.3B)	3.71E-02
NM_005325	histone cluster 1, H1a	3.77E-02
NM_005329	hyaluronan synthase 3	5.11E-03
NM_005332	hemoglobin, zeta	1.47E-02
NM_005333	holocytochrome c synthase	1.02E-03
NM_005346	heat shock 70kDa protein 1B	8.04E-03
NM_005368	myoglobin	2.13E-03
NM_005372	v-mos Moloney murine sarcoma viral oncogene homolog	3.72E-02
NM_005375	v-myb myeloblastosis viral oncogene homolog (avian)	2.35E-02
NM_005383	sialidase 2 (cytosolic sialidase)	1.41E-02
NM_005384	nuclear factor, interleukin 3 regulated	1.58E-02
NM_005389	protein-L-isoaspartate (D-aspartate) O-methyltransferase	3.32E-03
NM_005443	3'-phosphoadenosine 5'-phosphosulfate synthase 1	4.74E-02
NM_005448	bone morphogenetic protein 15	2.28E-02
NM_005452	WD repeat domain 46	3.94E-02
NM_005460	synuclein, alpha interacting protein	2.40E-02
NM_005469	acyl-CoA thioesterase 8	2.57E-02
NM_005486	target of myb1 (chicken)-like 1	6.94E-03

NM_005498	adaptor-related protein complex 1, mu 2 subunit	5.34E-03
NM_005511	melan-A	2.06E-02
NM_005518	3-hydroxy-3-methylglutaryl-CoA synthase 2 (mitochondrial)	1.94E-02
NM_005521	T-cell leukemia homeobox 1	1.41E-02
NM_005576	lysyl oxidase-like 1	3.37E-02
NM_005601	natural killer cell group 7 sequence	2.81E-02
NM_005631	smoothened, frizzled family receptor	2.52E-03
NM_005679	TATA box binding protein (TBP)-associated factor, RNA polymerase I, C, 110kDa	2.16E-02
NM_005689	ATP-binding cassette, sub-family B (MDR/TAP), member 6	3.15E-02
NM_005694	COX17 cytochrome c oxidase copper chaperone	4.52E-02
NM_005710	polyglutamine binding protein 1	4.10E-02
NM_005713	collagen, type IV, alpha 3 (Goodpasture antigen) binding protein	4.12E-02
NM_005714	potassium channel, subfamily K, member 7	3.93E-02
NM_005787	ALG3, alpha-1,3- mannosyltransferase	1.89E-02
NM_005802	topoisomerase I binding, arginine/serine-rich, E3 ubiquitin protein ligase	3.13E-02
NM_005845	ATP-binding cassette, sub-family C (CFTR/MRP), member 4	2.00E-04
NM_005850	splicing factor 3b, subunit 4, 49kDa	3.58E-02
NM_005855	receptor (G protein-coupled) activity modifying protein 1	1.41E-02
NM_005858	A kinase (PRKA) anchor protein 8	3.58E-02
NM_005860	follistatin-like 3 (secreted glycoprotein)	4.72E-02
NM_005863	neuroepithelial cell transforming 1	1.97E-02
NM_005866	sigma non-opioid intracellular receptor 1	4.97E-04
NM_005867	Down syndrome critical region gene 4	4.07E-03
NM_005875	eukaryotic translation initiation factor 1B	2.87E-02
NM_005884	p21 protein (Cdc42/Rac)-activated kinase 4	1.70E-03
NM_005891	acetyl-CoA acetyltransferase 2	1.12E-02
NM_005892	formin-like 1	5.94E-03
NM_005909	microtubule-associated protein 1B	3.37E-02
NM_005917	malate dehydrogenase 1, NAD (soluble)	1.54E-02
NM_006010	mesencephalic astrocyte-derived neurotrophic factor	4.46E-02
NM_006013	ribosomal protein L10	3.23E-03
NM_006017	prominin 1	1.71E-02
NM_006022	TSC22 domain family, member 1	9.09E-03

NM_006025	endonuclease, polyU-specific	1.73E-03
NM_006037	histone deacetylase 4	1.21E-02
NM_006042	heparan sulfate (glucosamine) 3-O-sulfotransferase 3A1	2.60E-02
NM_006044	histone deacetylase 6	5.94E-03
NM_006056	neuromedin U receptor 1	2.86E-02
NM_006066	aldo-keto reductase family 1, member A1 (aldehyde reductase)	2.91E-02
NM_006067	ER membrane protein complex subunit 8	1.64E-02
NM_006069	murine retrovirus integration site 1 homolog	2.76E-02
NM_006079	Cbp/p300-interacting transactivator, with Glu/Asp-rich carboxy-terminal domain, 2	3.54E-02
NM_006088	tubulin, beta 4B class IVb	4.91E-03
NM_006102	carboxypeptidase Q	6.82E-03
NM_006117	enoyl-CoA delta isomerase 2	3.02E-02
NM_006121	keratin 1	1.53E-02
NM_006152	lymphoid-restricted membrane protein	6.89E-03
NM_006156	neural precursor cell expressed, developmentally down-regulated 8	4.74E-02
NM_006157	NEL-like 1 (chicken)	4.69E-02
NM_006167	NK3 homeobox 1	2.02E-02
NM_006189	olfactory marker protein	2.96E-02
NM_006204	phosphodiesterase 6C, cGMP-specific, cone, alpha prime	2.18E-02
NM_006233	polymerase (RNA) II (DNA directed) polypeptide I, 14.5kDa	4.68E-02
NM_006236	POU class 3 homeobox 3	4.17E-02
NM_006251	protein kinase, AMP-activated, alpha 1 catalytic subunit	1.21E-02
NM_006262	peripherin	2.66E-02
NM_006279	ST3 beta-galactoside alpha-2,3-sialyltransferase 3	1.03E-02
NM_006290	tumor necrosis factor, alpha-induced protein 3	4.14E-02
NM_006296	vaccinia related kinase 2	5.78E-03
NM_006332	interferon, gamma-inducible protein 30	1.50E-02
NM_006335	translocase of inner mitochondrial membrane 17 homolog A (yeast)	2.71E-02
NM_006346	progesterone immunomodulatory binding factor 1	2.45E-02
NM_006347	peptidylprolyl isomerase H (cyclophilin H)	5.44E-03
NM_006348	component of oligomeric golgi complex 5	1.68E-02
NM_006350	follistatin	2.81E-02
NM_006351	translocase of inner mitochondrial membrane 44 homolog (yeast)	1.44E-03

NM_006359	solute carrier family 9, subfamily A (NHE6, cation proton antiporter 6), member 6	2.37E-02
NM_006378	sema domain, immunoglobulin domain (Ig), transmembrane domain (TM) and short cytoplasmic doma	3.76E-02
NM_006398	ubiquitin D	4.96E-02
NM_006403	neural precursor cell expressed, developmentally down-regulated 9	1.53E-02
NM_006406	peroxiredoxin 4	4.92E-03
NM_006415	serine palmitoyltransferase, long chain base subunit 1	4.30E-02
NM_006456	ST6 (alpha-N-acetyl-neuraminyl-2,3-beta-galactosyl-1,3)-N-acetylgalactosaminide alpha-2,6-sialyltrans	1.16E-02
NM_006480	regulator of G-protein signaling 14	2.61E-02
NM_006495	ecotropic viral integration site 2B	5.35E-04
NM_006513	seryl-tRNA synthetase	3.13E-02
NM_006536	chloride channel accessory 2	4.82E-02
NM_006540	nuclear receptor coactivator 2	4.06E-03
NM_006554	metaxin 2	2.75E-03
NM_006556	phosphomevalonate kinase	7.20E-03
NM_006561	CUGBP, Elav-like family member 2	7.33E-03
NM_006566	CD226 molecule	2.03E-02
NM_006570	Ras-related GTP binding A	2.39E-04
NM_006584	chaperonin containing TCP1, subunit 6B (zeta 2)	3.98E-02
NM_006590	ubiquitin specific peptidase 39	1.28E-02
NM_006620	HBS1-like (S. cerevisiae)	1.32E-02
NM_006633	IQ motif containing GTPase activating protein 2	4.25E-02
NM_006638	ribonuclease P/MRP 40kDa subunit	1.37E-02
NM_006674	HLA complex P5 (non-protein coding)	1.57E-02
NM_006682	fibrinogen-like 2	2.41E-02
NM_006687	actin-like 7A	3.01E-02
NM_006695	RUN domain containing 3A	2.34E-02
NM_006712	Fas-activated serine/threonine kinase	5.49E-03
NM_006718	pleiomorphic adenoma gene-like 1	1.11E-02
NM_006729	diaphanous-related formin 2	2.30E-03
NM_006738	A kinase (PRKA) anchor protein 13	6.14E-03
NM_006750	syntrophin, beta 2 (dystrophin-associated protein A1, 59kDa, basic component 2)	4.02E-02
NM_006751	sperm specific antigen 2	4.02E-02
NM_006756	transcription elongation factor A (SII), 1	6.47E-03

NM_006762	lysosomal protein transmembrane 5	3.67E-03
NM_006783	gap junction protein, beta 6, 30kDa	8.97E-04
NM_006790	myotilin	3.94E-02
NM_006793	peroxiredoxin 3	2.64E-02
NM_006799	protease, serine, 21 (testisin)	2.65E-02
NM_006810	protein disulfide isomerase family A, member 5	9.43E-03
NM_006811	serine incorporator 3	3.25E-03
NM_006824	EBNA1 binding protein 2	5.11E-03
NM_006825	cytoskeleton-associated protein 4	5.60E-05
NM_006830	ubiquinol-cytochrome c reductase, complex III subunit XI	6.74E-03
NM_006834	RAB32, member RAS oncogene family	4.67E-02
NM_006860	intraflagellar transport 27 homolog (Chlamydomonas)	4.96E-02
NM_006875	pim-2 oncogene	6.27E-04
NM_006876	UDP-GlcNAc:betaGal beta-1,3-N-acetylglucosaminyltransferase 1	2.96E-03
NM_006886	ATP synthase, H+ transporting, mitochondrial F1 complex, epsilon subunit	1.06E-02
NM_006891	crystallin, gamma D	2.75E-02
NM_006904	protein kinase, DNA-activated, catalytic polypeptide	7.64E-03
NM_006933	solute carrier family 5 (sodium/myo-inositol cotransporter), member 3	3.39E-02
NM_006977	zinc finger and BTB domain containing 25	3.36E-02
NM_006978	ring finger protein 113A	3.09E-03
NM_007056	CLK4-associating serine/arginine rich protein	3.88E-02
NM_007064	kalirin, RhoGEF kinase	9.28E-03
NM_007075	WD repeat domain 45	3.26E-03
NM_007080	LSM6 homolog, U6 small nuclear RNA associated (S. cerevisiae)	1.14E-02
NM_007085	follistatin-like 1	4.29E-02
NM_007100	ATP synthase, H+ transporting, mitochondrial Fo complex, subunit E	3.65E-02
NM_007106	ubiquitin-like 3	3.70E-02
NM_007108	transcription elongation factor B (SIII), polypeptide 2 (18kDa, elongin B)	4.15E-02
NM_007152	zinc finger protein 195	2.72E-02
NM_007171	protein-O-mannosyltransferase 1	4.28E-02
NM_007172	nucleoporin 50kDa	3.60E-03
NM_007178	serine/threonine kinase receptor associated protein	2.90E-02
NM_007187	WW domain binding protein 4	4.02E-02

NM_007188	ATP-binding cassette, sub-family B (MDR/TAP), member 8	2.46E-02
NM_007195	polymerase (DNA directed) iota	3.87E-02
NM_007211	Ras association (RalGDS/AF-6) domain family (N-terminal) member 8	1.53E-02
NM_007219	ring finger protein 24	4.60E-02
NM_007222	zinc fingers and homeoboxes 1	1.04E-02
NM_007229	protein kinase C and casein kinase substrate in neurons 2	3.75E-02
NM_007235	exportin, tRNA	1.56E-02
NM_007250	Kruppel-like factor 8	3.60E-02
NM_007254	polynucleotide kinase 3'-phosphatase	3.74E-02
NM_007283	monoglyceride lipase	2.72E-02
NM_007285	GABA(A) receptor-associated protein-like 2	3.61E-02
NM_007289	membrane metallo-endopeptidase	4.34E-05
NM_007326	cytochrome b5 reductase 3	2.23E-02
NM_007351	multimerin 1	2.70E-02
NM_007360	killer cell lectin-like receptor subfamily K, member 1	1.30E-02
NM_007366	phospholipase A2 receptor 1, 180kDa	4.25E-02
NM_012071	COMM domain containing 3	1.88E-02
NM_012072	CD93 molecule	1.55E-02
NM_012090	microtubule-actin crosslinking factor 1	3.64E-02
NM_012111	AHA1, activator of heat shock 90kDa protein ATPase homolog 1 (yeast)	1.35E-02
NM_012117	chromobox homolog 5	1.52E-02
NM_012143	tuftelin interacting protein 11	4.23E-04
NM_012155	echinoderm microtubule associated protein like 2	1.33E-02
NM_012160	F-box and leucine-rich repeat protein 4	4.46E-02
NM_012179	F-box protein 7	1.40E-02
NM_012192	translocase of inner mitochondrial membrane 10 homolog B (yeast)	2.54E-02
NM_012222	mutY homolog (E. coli)	4.29E-02
NM_012229	5'-nucleotidase, cytosolic II	1.78E-02
NM_012260	2-hydroxyacyl-CoA lyase 1	5.85E-03
NM_012338	tetraspanin 12	1.36E-02
NM_012341	GTP binding protein 4	2.93E-03
NM_012391	SAM pointed domain containing ets transcription factor	3.28E-03
NM_012404	acidic (leucine-rich) nuclear phosphoprotein 32 family, member D	1.29E-02

NM_012413	glutaminyl-peptide cyclotransferase	8.12E-04
NM_012427	kallikrein-related peptidase 5	4.93E-02
NM_012433	splicing factor 3b, subunit 1, 155kDa	4.58E-02
NM_012455	pleckstrin and Sec7 domain containing 4	2.18E-02
NM_012483	granulysin	1.63E-03
NM_013229	apoptotic peptidase activating factor 1	3.95E-03
NM_013235	drosha, ribonuclease type III	1.03E-03
NM_013236	ataxin 10	3.31E-02
NM_013238	DnaJ (Hsp40) homolog, subfamily C, member 15	1.62E-02
NM_013257	serum/glucocorticoid regulated kinase family, member 3	4.36E-02
NM_013291	cleavage and polyadenylation specific factor 1, 160kDa	2.99E-02
NM_013300	family with sequence similarity 216, member A	2.47E-02
NM_013301	coiled-coil domain containing 106	6.18E-03
NM_013345	G protein-coupled receptor 132	2.65E-02
NM_013363	procollagen C-endopeptidase enhancer 2	4.91E-02
NM_013385	cytohesin 4	2.66E-02
NM_013387	ubiquinol-cytochrome c reductase, complex III subunit X	1.30E-02
NM_013388	prolactin regulatory element binding	1.15E-02
NM_013393	FtsJ RNA methyltransferase homolog 2 (E. coli)	4.52E-02
NM_013437	low density lipoprotein receptor-related protein 12	4.62E-02
NM_013438	ubiquilin 1	2.19E-02
NM_013442	stomatin (EPB72)-like 2	1.46E-02
NM_013446	makorin ring finger protein 1	4.04E-02
NM_013952	paired box 8	2.63E-03
NM_013974	dimethylarginine dimethylaminohydrolase 2	2.87E-02
NM_014002	inhibitor of kappa light polypeptide gene enhancer in B-cells, kinase epsilon	5.74E-04
NM_014017	late endosomal/lysosomal adaptor, MAPK and MTOR activator 2	1.57E-02
NM_014018	mitochondrial ribosomal protein S28	2.20E-02
NM_014046	mitochondrial ribosomal protein S18B	2.58E-02
NM_014056	HIG1 hypoxia inducible domain family, member 1A	2.67E-02
NM_014059	regulator of cell cycle	3.53E-02
NM_014060	malignant T cell amplified sequence 1	2.95E-02
NM_014061	melanoma antigen family H, 1	3.23E-02

NM_014121	adenosylhomocysteinase-like 1	8.35E-03
NM_014125	polymerase (DNA directed), theta	3.07E-02
NM_014143	CD274 molecule	4.20E-02
NM_014145	transmembrane protein 230	4.52E-02
NM_014156	DDB1 and CUL4 associated factor 13	4.42E-02
NM_014165	NADH dehydrogenase (ubiquinone) complex I, assembly factor 4	3.56E-03
NM_014175	mitochondrial ribosomal protein L15	8.47E-03
NM_014187	transmembrane protein 208	3.37E-02
NM_014205	zinc finger, HIT-type containing 2	3.70E-02
NM_014222	NADH dehydrogenase (ubiquinone) 1 alpha subcomplex, 8, 19kDa	1.66E-02
NM_014230	signal recognition particle 68kDa	2.08E-02
NM_014232	vesicle-associated membrane protein 2 (synaptobrevin 2)	1.01E-02
NM_014247	Rap guanine nucleotide exchange factor (GEF) 2	2.08E-02
NM_014252	solute carrier family 25 (mitochondrial carrier; ornithine transporter) member 15	8.17E-03
NM_014302	Sec61 gamma subunit	2.44E-02
NM_014305	TDP-glucose 4,6-dehydratase	2.43E-02
NM_014317	prenyl (decaprenyl) diphosphate synthase, subunit 1	3.06E-03
NM_014330	protein phosphatase 1, regulatory subunit 15A	7.43E-03
NM_014333	cell adhesion molecule 1	9.37E-03
NM_014335	EP300 interacting inhibitor of differentiation 1	1.49E-03
NM_014342	mitochondrial carrier 2	2.04E-02
NM_014344	four jointed box 1 (Drosophila)	4.34E-02
NM_014362	3-hydroxyisobutyryl-CoA hydrolase	1.79E-02
NM_014364	glyceraldehyde-3-phosphate dehydrogenase, spermatogenic	2.70E-02
NM_014373	G protein-coupled receptor 160	2.51E-02
NM_014383	zinc finger and BTB domain containing 32	4.77E-02
NM_014408	trafficking protein particle complex 3	1.27E-02
NM_014433	rhabdoid tumor deletion region gene 1	3.22E-02
NM_014447	ADP-ribosylation factor interacting protein 1	1.72E-02
NM_014461	contactin 6	5.40E-03
NM_014466	tektin 2 (testicular)	5.64E-03
NM_014473	DIM1 dimethyladenosine transferase 1 homolog (S. cerevisiae)	3.52E-02
NM_014483	RNA binding motif, single stranded interacting protein 3	1.22E-03

NM_014498	golgi integral membrane protein 4	3.81E-02
NM_014499	purinergic receptor P2Y, G-protein coupled, 10	1.23E-02
NM_014519	zinc finger protein 232	3.10E-02
NM_014520	MYB binding protein (P160) 1a	1.43E-03
NM_014522	protocadherin 11 X-linked	3.52E-02
NM_014548	tropomodulin 2 (neuronal)	2.44E-02
NM_014583	LIM and cysteine-rich domains 1	1.23E-02
NM_014593	CXXC finger protein 1	4.32E-02
NM_014596	zinc ribbon domain containing 1	3.24E-02
NM_014618	deleted in bladder cancer 1	4.48E-03
NM_014622	von Willebrand factor A domain containing 5A	4.21E-04
NM_014623	male-enhanced antigen 1	2.30E-03
NM_014633	Ctr9, Paf1/RNA polymerase II complex component, homolog (S. cerevisiae)	4.18E-02
NM_014654	syndecan 3	3.78E-02
NM_014660	PHD finger protein 14	2.11E-02
NM_014673	ER membrane protein complex subunit 2	5.19E-04
NM_014677	regulating synaptic membrane exocytosis 2	1.34E-02
NM_014678	protein phosphatase 6, regulatory subunit 2	4.10E-03
NM_014681	DEAH (Asp-Glu-Ala-His) box polypeptide 34	1.94E-02
NM_014690	family with sequence similarity 131, member B	1.36E-02
NM_014693	endothelin converting enzyme 2	3.74E-02
NM_014700	RAB11 family interacting protein 3 (class II)	1.60E-02
NM_014704	centrosomal protein 104kDa	1.99E-02
NM_014705	dedicator of cytokinesis 4	1.33E-03
NM_014717	zinc finger protein 536	3.72E-02
NM_014722	family with sequence similarity 65, member B	2.09E-02
NM_014725	StAR-related lipid transfer (START) domain containing 8	5.40E-03
NM_014727	lysine (K)-specific methyltransferase 2B	5.54E-03
NM_014729	thymocyte selection-associated high mobility group box	4.60E-02
NM_014736	KIAA0101	4.14E-02
NM_014763	mitochondrial ribosomal protein L19	2.13E-02
NM_014764	DAZ associated protein 2	1.54E-02
NM_014774	EF-hand calcium binding domain 14	3.89E-02

NM_014800	engulfment and cell motility 1	5.55E-03
NM_014801	pecanex-like 2 (Drosophila)	3.55E-02
NM_014809	KIAA0319	1.51E-02
NM_014814	proteasome (prosome, macropain) 26S subunit, non-ATPase, 6	1.48E-02
NM_014824	FCH and double SH3 domains 2	2.02E-02
NM_014830	zinc finger and BTB domain containing 39	2.45E-03
NM_014857	RAB GTPase activating protein 1-like	2.46E-03
NM_014863	carbohydrate (N-acetylgalactosamine 4-sulfate 6-O) sulfotransferase 15	4.54E-03
NM_014867	kelch repeat and BTB (POZ) domain containing 11	2.66E-02
NM_014874	mitofusin 2	2.16E-02
NM_014878	KIAA0020	9.95E-03
NM_014882	Rho GTPase activating protein 25	4.58E-02
NM_014911	AP2 associated kinase 1	1.96E-03
NM_014918	chondroitin sulfate synthase 1	7.60E-03
NM_014922	NLR family, pyrin domain containing 1	6.62E-04
NM_014925	R3H domain containing 2	1.99E-02
NM_014933	SEC31 homolog A (S. cerevisiae)	2.03E-04
NM_014945	actin binding LIM protein family, member 3	2.84E-03
NM_014965	trafficking protein, kinesin binding 1	1.78E-02
NM_014967	FANCD2/FANCI-associated nuclease 1	1.93E-02
NM_014969	WD repeat domain 47	1.42E-02
NM_015046	senataxin	9.60E-03
NM_015193	activity-regulated cytoskeleton-associated protein	2.59E-02
NM_015322	fem-1 homolog b (C. elegans)	2.03E-03
NM_015492	chromosome 15 open reading frame 39	2.03E-02
NM_015507	EGF-like-domain, multiple 6	1.23E-02
NM_015556	signal-induced proliferation-associated 1 like 1	4.92E-02
NM_015607	chromatin target of PRMT1	3.44E-02
NM_015626	WD repeat and SOCS box containing 1	2.67E-03
NM_015640	SERPINE1 mRNA binding protein 1	6.23E-03
NM_015722	calcyon neuron-specific vesicular protein	2.28E-02
NM_015917	glutathione S-transferase kappa 1	6.34E-03
NM_015920	ribosomal protein S27-like	1.59E-02

NM_015922	NAD(P) dependent steroid dehydrogenase-like	5.16E-03
NM_015925	lipolysis stimulated lipoprotein receptor	4.16E-02
NM_015926	testis expressed 264	3.18E-02
NM_015932	proteasome maturation protein	4.95E-02
NM_015965	NADH dehydrogenase (ubiquinone) 1 alpha subcomplex, 13	3.11E-02
NM_015972	polymerase (RNA) I polypeptide D, 16kDa	4.82E-02
NM_015999	adiponectin receptor 1	2.73E-02
NM_016001	UTP18 small subunit (SSU) processome component homolog (yeast)	4.90E-03
NM_016010	zinc finger, C2HC-type containing 1A	2.21E-02
NM_016022	APH1A gamma secretase subunit	2.70E-02
NM_016048	isochorismatase domain containing 1	4.79E-02
NM_016053	coiled-coil domain containing 53	5.11E-05
NM_016058	TP53RK binding protein	1.97E-02
NM_016063	HD domain containing 2	3.73E-03
NM_016065	mitochondrial ribosomal protein S16	2.83E-02
NM_016067	mitochondrial ribosomal protein S18C	1.73E-03
NM_016095	GINS complex subunit 2 (Psf2 homolog)	4.11E-02
NM_016100	N(alpha)-acetyltransferase 20, NatB catalytic subunit	3.66E-02
NM_016107	zinc finger RNA binding protein	3.20E-02
NM_016122	coiled-coil domain containing 41	2.11E-02
NM_016180	solute carrier family 45, member 2	2.78E-02
NM_016200	N(alpha)-acetyltransferase 38, NatC auxiliary subunit	4.19E-02
NM_016205	platelet derived growth factor C	9.94E-03
NM_016207	cleavage and polyadenylation specific factor 3, 73kDa	9.81E-03
NM_016218	polymerase (DNA directed) kappa	1.29E-03
NM_016248	A kinase (PRKA) anchor protein 11	3.12E-02
NM_016255	family with sequence similarity 8, member A1	1.88E-02
NM_016270	Kruppel-like factor 2 (lung)	9.14E-03
NM_016274	pleckstrin homology domain containing, family O member 1	4.14E-03
NM_016287	heterochromatin protein 1, binding protein 3	4.05E-02
NM_016289	calcium binding protein 39	1.30E-02
NM_016301	GPN-loop GTPase 3	1.87E-02
NM_016303	WW domain binding protein 5	1.24E-02

NM_016304	ribosomal L24 domain containing 1	7.87E-04
NM_016306	DnaJ (Hsp40) homolog, subfamily B, member 11	4.46E-02
NM_016310	polymerase (RNA) III (DNA directed) polypeptide K, 12.3 kDa	8.18E-03
NM_016328	GTF2I repeat domain containing 1	2.12E-02
NM_016329	Scm-like with four mbt domains 1	8.93E-03
NM_016331	zinc finger protein 639	2.56E-02
NM_016332	methionine sulfoxide reductase B1	1.91E-02
NM_016360	translational activator of mitochondrially encoded cytochrome c oxidase I	3.60E-02
NM_016364	dual specificity phosphatase 13	1.25E-02
NM_016404	tRNA methyltransferase 11-2 homolog (S. cerevisiae)	2.25E-02
NM_016411	chromosome 11 open reading frame 73	5.33E-03
NM_016438	HIG1 hypoxia inducible domain family, member 1B	8.43E-03
NM_016448	denticleless E3 ubiquitin protein ligase homolog (Drosophila)	4.19E-02
NM_016468	COX16 cytochrome c oxidase assembly homolog (S. cerevisiae)	3.92E-02
NM_016497	mitochondrial ribosomal protein L51	2.94E-02
NM_016499	transmembrane protein 216	1.68E-04
NM_016500	polysaccharide biosynthesis domain containing 1	2.30E-02
NM_016504	mitochondrial ribosomal protein L27	1.41E-03
NM_016505	zinc finger, CCHC domain containing 17	1.65E-02
NM_016508	cyclin-dependent kinase-like 3	4.67E-03
NM_016509	C-type lectin domain family 1, member B	3.91E-02
NM_016510	selenocysteine lyase	1.75E-03
NM_016550	cyclin-dependent kinase 2 interacting protein	1.12E-02
NM_016553	nucleoporin 62kDa	1.48E-02
NM_016558	SCAN domain containing 1	3.62E-02
NM_016563	RAS-like, family 12	1.16E-02
NM_016589	translocase of inner mitochondrial membrane domain containing 1	4.96E-02
NM_016617	ubiquitin-fold modifier 1	9.30E-03
NM_016625	arginine/serine-rich coiled-coil 1	1.00E-02
NM_016638	ADP-ribosylation-like factor 6 interacting protein 4	4.81E-02
NM_016929	chloride intracellular channel 5	4.90E-02
NM_016943	taste receptor, type 2, member 3	4.67E-02
NM_017413	apelin	3.64E-02

NM_017415	kelch-like family member 3	1.90E-02
NM_017446	mitochondrial ribosomal protein L39	1.45E-02
NM_017458	major vault protein	2.52E-02
NM_017459	microfibrillar-associated protein 2	4.13E-02
NM_017544	NFKB repressing factor	1.46E-02
NM_017548	CDV3 homolog (mouse)	3.67E-02
NM_017575	smg-6 homolog, nonsense mediated mRNA decay factor (C. elegans)	4.23E-02
NM_017577	GRAM domain containing 1C	2.81E-02
NM_017596	kinesin family member 21B	1.48E-02
NM_017617	notch 1	3.45E-02
NM_017637	basonuclin 2	1.21E-02
NM_017675	cadherin-related family member 2	2.25E-02
NM_017695	synaptotagmin-like 2	2.34E-02
NM_017712	pyroglutamyl-peptidase I	1.20E-02
NM_017726	protein phosphatase 1, regulatory (inhibitor) subunit 14D	4.40E-02
NM_017729	EPS8-like 1	4.87E-02
NM_017742	zinc finger, CCHC domain containing 2	2.16E-02
NM_017755	NOP2/Sun RNA methyltransferase family, member 2	2.82E-02
NM_017777	Meckel syndrome, type 1	4.46E-02
NM_017792	mitogen-activated protein kinase kinase kinase kinase 4	6.62E-03
NM_017817	RAB20, member RAS oncogene family	3.78E-02
NM_017823	dual specificity phosphatase 23	2.40E-02
NM_017832	family with sequence similarity 206, member A	4.17E-03
NM_017836	solute carrier family 41, member 3	1.79E-02
NM_017849	transmembrane protein 127	1.58E-02
NM_017851	poly (ADP-ribose) polymerase family, member 16	2.38E-03
NM_017881	nicotinamide riboside kinase 1	3.15E-02
NM_017885	host cell factor C1 regulator 1 (XPO1 dependent)	1.17E-02
NM_017895	DEAD (Asp-Glu-Ala-Asp) box polypeptide 27	4.88E-02
NM_017911	family with sequence similarity 118, member A	1.13E-02
NM_017913	cell division cycle 37-like 1	2.08E-02
NM_017915	PARP1 binding protein	9.85E-03
NM_017949	CUE domain containing 1	3.57E-02

NM_017956	tRNA methyltransferase 12 homolog (S. cerevisiae)	3.98E-02
NM_017980	LIM and senescent cell antigen-like domains 2	1.47E-02
NM_017986	solute carrier family 52, riboflavin transporter, member 1	2.72E-02
NM_017998	chromosome 9 open reading frame 40	2.76E-02
NM_018011	arginine and glutamate rich 1	3.60E-02
NM_018040	G patch domain containing 2	2.16E-02
NM_018060	isoleucyl-tRNA synthetase 2, mitochondrial	2.79E-03
NM_018073	tripartite motif containing 68	4.07E-02
NM_018084	coiled-coil domain containing 88A	3.99E-02
NM_018105	THAP domain containing, apoptosis associated protein 1	1.33E-02
NM_018113	limb region 1 homolog (mouse)-like	2.33E-02
NM_018117	WD repeat domain 11	1.69E-02
NM_018127	elaC ribonuclease Z 2	2.94E-02
NM_018135	mitochondrial ribosomal protein S18A	4.19E-03
NM_018139	dynein, axonemal, assembly factor 2	9.25E-03
NM_018141	mitochondrial ribosomal protein S10	4.63E-02
NM_018172	family with sequence similarity 86, member C1	3.92E-04
NM_018179	activating transcription factor 7 interacting protein	2.09E-02
NM_018186	chromosome 1 open reading frame 112	1.12E-02
NM_018203	kelch domain containing 8A	4.89E-02
NM_018210	carbohydrate kinase domain containing	8.22E-04
NM_018215	paraneoplastic Ma antigen family-like 1	5.64E-03
NM_018225	smu-1 suppressor of mec-8 and unc-52 homolog (C. elegans)	3.87E-02
NM_018227	ubiquitin-like modifier activating enzyme 6	2.18E-02
NM_018230	nucleoporin 133kDa	4.27E-03
NM_018239	leucine rich repeat containing 20	7.77E-03
NM_018248	nei endonuclease VIII-like 3 (E. coli)	4.39E-02
NM_018249	CDK5 regulatory subunit associated protein 2	6.39E-05
NM_018250	integrator complex subunit 9	2.07E-02
NM_018275	chromosome 7 open reading frame 43	3.99E-03
NM_018283	nudix (nucleoside diphosphate linked moiety X)-type motif 15	2.67E-03
NM_018318	coiled-coil domain containing 91	2.27E-02
NM_018326	GTPase, IMAP family member 4	4.81E-02

NM_018340	calcineurin-like phosphoesterase domain containing 1	7.69E-03
NM_018353	MIS18 binding protein 1	3.84E-02
NM_018358	ATP-binding cassette, sub-family F (GCN20), member 3	4.91E-02
NM_018365	meiosis-specific nuclear structural 1	1.62E-02
NM_018367	alkaline ceramidase 3	2.65E-02
NM_018368	LMBR1 domain containing 1	3.11E-02
NM_018383	WD repeat domain 33	1.26E-02
NM_018391	ubiquitin specific peptidase 48	3.51E-02
NM_018401	serine/threonine kinase 32B	4.89E-02
NM_018402	interleukin 26	4.54E-02
NM_018413	carbohydrate (chondroitin 4) sulfotransferase 11	1.55E-02
NM_018416	forkhead box J2	2.98E-02
NM_018434	ring finger protein 130	8.08E-03
NM_018444	pyruvate dehyrogenase phosphatase catalytic subunit 1	8.83E-03
NM_018449	ubiquitin associated protein 2	1.97E-02
NM_018460	Rho GTPase activating protein 15	3.01E-02
NM_018465	plasminogen receptor, C-terminal lysine transmembrane protein	2.68E-02
NM_018467	unconventional SNARE in the ER 1 homolog (S. cerevisiae)	7.98E-03
NM_018515	solute carrier organic anion transporter family, member 4C1	7.68E-03
NM_018556	signal-regulatory protein gamma	6.76E-03
NM_018569	adaptor-related protein complex 1 associated regulatory protein	2.16E-02
NM_018630	derlin 1	2.07E-02
NM_018643	triggering receptor expressed on myeloid cells 1	1.61E-04
NM_018648	NOP10 ribonucleoprotein	1.46E-02
NM_018649	H2A histone family, member Y2	6.54E-03
NM_018666	sarcoma antigen 1	1.24E-03
NM_018686	cytidine monophosphate N-acetylneuraminic acid synthetase	3.53E-02
NM_018691	family with sequence similarity 114, member A2	3.59E-02
NM_018840	chromosome 20 open reading frame 24	2.26E-02
NM_018944	MIS18 kinetochore protein homolog A (S. pombe)	9.11E-04
NM_018967	syntrophin, gamma 1	4.83E-03
NM_018971	G protein-coupled receptor 27	1.55E-02
NM_018973	dolichyl-phosphate mannosyltransferase polypeptide 3	3.65E-02

NM_018977	neuroligin 3	1.11E-02
NM_018988	glucose-fructose oxidoreductase domain containing 1	3.20E-03
NM_019037	exosome component 4	2.09E-03
NM_019095	cardiolipin synthase 1	3.62E-03
NM_019101	apolipoprotein M	1.94E-02
NM_019106	septin 3	5.72E-03
NM_019598	kallikrein-related peptidase 12	3.51E-02
NM_019604	cytotoxic and regulatory T cell molecule	3.01E-02
NM_019609	carboxypeptidase X (M14 family), member 1	2.40E-02
NM_019617	gastrokine 1	2.47E-02
NM_019619	par-3 partitioning defective 3 homolog (C. elegans)	2.40E-02
NM_019845	reprimo, TP53 dependent G2 arrest mediator candidate	1.09E-02
NM_019892	inositol polyphosphate-5-phosphatase, 72 kDa	4.16E-02
NM_020119	zinc finger CCCH-type, antiviral 1	2.00E-02
NM_020143	partner of NOB1 homolog (S. cerevisiae)	1.17E-02
NM_020157	otoraplin	6.81E-03
NM_020158	exosome component 5	1.12E-02
NM_020163	sema domain, immunoglobulin domain (Ig), short basic domain, secreted, (semaphorin) 3G	2.45E-03
NM_020168	p21 protein (Cdc42/Rac)-activated kinase 6	3.91E-02
NM_020183	aryl hydrocarbon receptor nuclear translocator-like 2	4.11E-02
NM_020191	mitochondrial ribosomal protein S22	4.29E-02
NM_020198	coiled-coil domain containing 47	3.08E-02
NM_020199	chromosome 5 open reading frame 15	1.06E-02
NM_020240	CDC42 small effector 2	1.25E-02
NM_020243	translocase of outer mitochondrial membrane 22 homolog (yeast)	1.41E-02
NM_020348	cyclin M1	8.73E-03
NM_020353	phospholipid scramblase 4	2.26E-02
NM_020356	Cas scaffolding protein family member 4	3.15E-03
NM_020367	poly (ADP-ribose) polymerase family, member 11	2.72E-02
NM_020375	chromosome 12 open reading frame 5	3.26E-02
NM_020377	cysteinyl leukotriene receptor 2	1.48E-02
NM_020401	nucleoporin 107kDa	2.57E-02
NM_020403	protocadherin 9	5.75E-03

NM_020415	resistin	6.19E-03
NM_020524	pre-B-cell leukemia homeobox interacting protein 1	1.98E-02
NM_020528	poly(rC) binding protein 3	4.74E-02
NM_020530	oncostatin M	2.61E-02
S74620	protein kinase C, eta	3.25E-02
S80797	insulin-like growth factor 2 receptor	7.06E-03
S82470	membrane bound O-acyltransferase domain containing 7	1.56E-03
S82769	gamma-aminobutyric acid (GABA) A receptor, gamma 3	2.29E-03
S94541	solute carrier family 25 (mitochondrial iron transporter), member 37	3.12E-03
U09410	zinc finger protein 131	3.77E-02
U15553	transcription factor 7-like 1 (T-cell specific, HMG-box)	1.01E-02
U18937	histidyl-tRNA synthetase 2, mitochondrial	2.02E-02
U25750	transmembrane protein 106A	3.85E-03
U27655	regulator of G-protein signaling 3	7.93E-03
U41387	DEAD (Asp-Glu-Ala-Asp) box helicase 21	1.74E-02
U57962	NEDD4 binding protein 2-like 1	3.53E-02
U66048	long intergenic non-protein coding RNA 894	1.27E-02
U67847	ST6 beta-galactosamide alpha-2,6-sialyltranferase 1	4.92E-02
U69263	matrilin 2	1.46E-02
U69645	zinc finger protein 32	5.38E-04
U73799	dynactin 1	1.08E-02
U74612	forkhead box M1	1.40E-02
U79248	long intergenic non-protein coding RNA 667	2.94E-02
U79273	eukaryotic translation initiation factor 4A1	2.78E-02
U80018	proline dehydrogenase (oxidase) 2	4.77E-02
U81599	homeobox B13	3.15E-02
U89277	polyhomeotic homolog 1 (Drosophila)	3.02E-02
U90653	zinc finger, DHHC-type containing 1	1.95E-02
U92816	endogenous retrovirus group H, member 6	3.52E-02
U93181	SET binding factor 1	8.16E-03
U94386	RNA binding motif protein, Y-linked, family 3, member A pseudogene	4.82E-02
V00539	interferon, alpha 22, pseudogene	1.21E-02
X02067	RNA, 7SL, cytoplasmic 2	2.74E-02

X15183	heat shock protein 90kDa alpha (cytosolic), class A member 1	1.96E-02
X52350	zinc finger protein 25	6.33E-03
X52354	zinc finger protein 551	2.29E-02
X52355	zinc finger protein 28	4.88E-02
X52882	t-complex 1	8.20E-03
X59417	proteasome (prosome, macropain) subunit, alpha type, 6	1.73E-02
X64979	olfactory receptor, family 1, subfamily J, member 4	1.57E-02
X73502	keratin 20	8.86E-03
X74794	minichromosome maintenance complex component 4	1.02E-02
X75684	transmembrane 4 L six family member 1	5.63E-03
X76386	epididymal protein 3B	2.39E-02
X87825	olfactory receptor, family 7, subfamily E, member 47 pseudogene	3.17E-03
X89657	ADAM metallopeptidase domain 3A (pseudogene)	3.28E-02
X91221	solute carrier family 8 (sodium/calcium exchanger), member 1	7.17E-03
X96646	small nucleolar RNA, C/D box 46	5.21E-03
X97303	zinc finger, matrin-type 2	7.87E-03
X98259	M-phase phosphoprotein 8	9.35E-03
X99631	homeobox C12	1.44E-02
X99962	RAB39A, member RAS oncogene family	3.36E-02
Y10210	leucine rich repeat containing 16A	1.24E-02
Y10483	protein kinase, X-linked, pseudogene 1	1.61E-03
Y10529	olfactory receptor, family 7, subfamily E, member 24	3.41E-02
Y11158	small nucleolar RNA, H/ACA box 64	5.30E-03
Y11162	small nucleolar RNA, H/ACA box 68	3.92E-02
Y11897	cysteine-rich hydrophobic domain 1	2.69E-02
Y12839	meiosis 1 associated protein	4.66E-02
Y17867	tenascin XB	9.41E-03
Z11849	growth hormone receptor	5.58E-03
Z22780	cylicin, basic protein of sperm head cytoskeleton 1	4.84E-02
Z25424	MAP kinase interacting serine/threonine kinase 2	3.21E-03
Z25431	NIMA-related kinase 1	2.07E-02
Z25436	muscle, skeletal, receptor tyrosine kinase	4.31E-02
Z50169	sodium channel, voltage gated, type VIII, alpha subunit	4.00E-02

GSE 6535: Gram negative bacterial infected patients - Differentially expressed genes

TLR 4 Training set

ID	Gene.title	P.Value
AB000520	SH2B adaptor protein 2	0.0004725
AB002298	PDZ domain containing 2	0.0051586
AB002301	microtubule associated serine/threonine kinase family member 4	0.0020171
AB002330	U2 snRNP-associated SURP domain containing	0.0207095
AB002347	ubiquitin protein ligase E3 component n-recognin 2	0.0010932
AB002354	pleckstrin homology domain containing, family M (with RUN domain) member 1	0.0113217
AB002365	prune homolog 2 (Drosophila)	0.031962
AB002368	exportin 6	0.0034251
AB002437	EPB41L4A antisense RNA 1	0.0000755
AB007859	zinc finger, ZZ-type with EF-hand domain 1	0.0264288
AB007864	autophagy related 2A	0.0195962
AB007880	SEC14-like 5 (S. cerevisiae)	0.0127705
AB007890	KIAA0430	0.0202173
AB007916	solute carrier family 35, member E2B	0.0014679
AB007965	cysteine/histidine-rich 1	0.0161977
AB007972	protein phosphatase 1, regulatory subunit 12B	0.0043453
AB011089	tripartite motif containing 2	0.0141828
AB011114	Sfi1 homolog, spindle assembly associated (yeast)	0.0038941
AB011131	piccolo presynaptic cytomatrix protein	0.03385
AB011163	kinesin family member 1B	0.0458048
AB011166	structural maintenance of chromosomes 5	0.0312984
AB011173	lysine (K)-specific demethylase 1A	0.0166676
AB013103	membrane-spanning 4-domains, subfamily A, member 5	0.0475193
AB014550	structural maintenance of chromosomes flexible hinge domain containing 1	0.0037261
AB014557	obscurin-like 1	0.0210698
AB015349	GRB2 associated, regulator of MAPK1-like	0.0159737
AB018274	La ribonucleoprotein domain family, member 1	0.0315239
AB018319	UFM1-specific ligase 1	0.004328
AB018322	transmembrane and coiled-coil domain family 1	0.0499809

AB018325	ArfGAP with RhoGAP domain, ankyrin repeat and PH domain 1	0.0000164
AB020632	KIAA0825	0.0073983
AB020710	EH domain binding protein 1	0.018007
AB020713	nucleoporin 210kDa	0.0218849
AB023195	additional sex combs like 1 (Drosophila)	0.0382536
AB023230	FERM domain containing 4B	0.0493782
AB023420	heat shock 70kDa protein 4	0.0019432
AB024704	TPX2, microtubule-associated, homolog (Xenopus laevis)	0.0027592
AB025194	protein tyrosine phosphatase, non-receptor type 23	0.0139485
AB026542	WAS protein family, member 2	0.0184104
AB028998	tensin like C1 domain containing phosphatase (tensin 2)	0.0013569
AB028999	SET domain containing 1B	0.0240339
AB029033	IQ motif and Sec7 domain 3	0.0252314
AB032945	myosin VB	0.0247091
AB032953	teneurin transmembrane protein 2	0.0482277
AB032969	KIAA1143	0.0005259
AB032986	ISY1-RAB43 readthrough	0.0198033
AB032988	thioredoxin-related transmembrane protein 4	0.0397225
AB032991	Nedd4 family interacting protein 2	0.0071271
AB032993	GRIP1 associated protein 1	0.0310445
AB033009	paraneoplastic Ma antigen family-like 2	0.0170467
AB033046	glutamate receptor, ionotropic, delta 1	0.0250613
AB033073	sulfatase 2	0.0002613
AB033092	metastasis associated 1 family, member 3	0.0397925
AB033093	KAT8 regulatory NSL complex subunit 1	0.0140107
AB033094	poly (ADP-ribose) polymerase family, member 14	0.041236
AB033100	phosphatase domain containing, paladin 1	0.0388302
AB033105	KIAA1279	0.0017877
AB035130	cytochrome P450, family 4, subfamily F, polypeptide 12	0.0014517
AB036063	ribonucleotide reductase M2 B (TP53 inducible)	0.0434834
AB037716	SH3 and PX domains 2B	0.0006301
AB037722	HECT, C2 and WW domain containing E3 ubiquitin protein ligase 2	0.0157678
AB037784	neutral cholesterol ester hydrolase 1	0.0206269

AB037785	microtubule associated monooxygenase, calponin and LIM domain containing 3	0.0222877
AB037791	family with sequence similarity 214, member A	0.0189088
AB037792	KIAA1109	0.0210585
AB037836	phosphatidylinositol-3,4,5-trisphosphate-dependent Rac exchange factor 1	0.0008373
AB037851	KIAA1430	0.0003671
AB039920	KCNQ1 downstream neighbor (non-protein coding)	0.0388297
AB040812	p21 protein (Cdc42/Rac)-activated kinase 7	0.0289515
AB040880	BAH domain and coiled-coil containing 1	0.0496707
AB040887	S-phase cyclin A-associated protein in the ER	0.0115378
AB040891	SLAIN motif family, member 2	0.0258027
AB040937	ERI1 exoribonuclease family member 2	0.0239374
AB040938	coiled-coil domain containing 146	0.0284754
AB046777	AT rich interactive domain 2 (ARID, RFX-like)	0.0308429
AB046781	uveal autoantigen with coiled-coil domains and ankyrin repeats	0.0354945
AB046830	mesoderm induction early response 1 homolog (Xenopus laevis)	0.0348738
AB046859	obscurin, cytoskeletal calmodulin and titin-interacting RhoGEF	0.0342412
AB046861	ankyrin repeat domain 36	0.0145478
AF007132	abhydrolase domain containing 5	0.011966
AF007152	abhydrolase domain containing 3	0.0034462
AF007217	thyroid hormone receptor interactor 11	0.0320981
AF010236	sarcoglycan, delta (35kDa dystrophin-associated glycoprotein)	0.0043199
AF016495	aquaporin 9	0.0058556
AF018254	peroxisome proliferator-activated receptor alpha	0.0467727
AF020089	BR serine/threonine kinase 2	0.0424122
AF020314	CD300a molecule	0.0061588
AF026942	radical S-adenosyl methionine domain containing 2	0.0283464
AF026944	interferon-induced protein with tetratricopeptide repeats 2	0.0157073
AF029777	K(lysine) acetyltransferase 2A	0.014888
AF039942	CREB/ATF bZIP transcription factor	0.0424594
AF040964	HAUS augmin-like complex, subunit 3	0.0159984
AF043324	N-myristoyltransferase 1	0.030053
AF047002	Aly/REF export factor	0.0150016
AF050198	R3H domain and coiled-coil containing 1-like	0.032806

AF052146	family with sequence similarity 105, member A	5.072E-05
AF052497	myozenin 3	0.0209181
AF064729	exportin 7	0.0401938
AF064770	diacylglycerol kinase, alpha 80kDa	0.031253
AF064876	hyperpolarization activated cyclic nucleotide-gated potassium channel 1	0.0100901
AF067972	DNA (cytosine-5-)-methyltransferase 3 alpha	0.0432602
AF069469	sterol-C5-desaturase	0.0237185
AF070584	ATP synthase mitochondrial F1 complex assembly factor 2	0.0093843
AF070637	N-terminal EF-hand calcium binding protein 2	0.0245657
AF075083	keratin 23 (histone deacetylase inducible)	0.0301245
AF075085	oncoprotein induced transcript 3	0.0014155
AF076617	solute carrier family 25 (mitochondrial carrier; adenine nucleotide translocator), member 6	0.029038
AF077188	cullin 4A	0.0354858
AF083117	uncharacterized LOC100506513	0.0369213
AF085351	striatin, calmodulin binding protein	0.0195084
AF085855	pregnancy-associated plasma protein A, pappalysin 1	0.044819
AF085867	abl-interactor 2	0.0492625
AF085880	chromosome 5 open reading frame 24	0.0131755
AF085904	retinoblastoma binding protein 4	0.0071116
AF086028	v-erb-b2 erythroblastic leukemia viral oncogene homolog 3 (avian)	0.0438801
AF086029	chromosome 12 open reading frame 79	0.0362234
AF086057	progestin and adipoQ receptor family member IV	0.0342052
AF086081	COMM domain containing 6	0.033293
AF086095	COX assembly mitochondrial protein 1 homolog (S. cerevisiae)	0.038829
AF086098	chromosome 2 open reading frame 72	0.049062
AF086115	PAP associated domain containing 4	0.0032612
AF086127	zinc finger protein 641	0.0274532
AF086148	uncharacterized LOC642533	0.0065092
AF086198	hexokinase 2	0.0064808
AF086234	mitochondrial nucleoid factor 1	0.005286
AF086244	potassium channel regulator	0.0219136
AF086274	ADARB2 antisense RNA 1	0.0061225
AF086393	nanos homolog 1 (Drosophila)	0.0220315

AF086406	CUE domain containing 2	0.0145401
AF086448	phosphorylated adaptor for RNA export	0.0077235
AF088010	transmembrane channel-like 7	0.0228677
AF090099	centrosomal protein 68kDa	0.0034409
AF091034	RAB22A, member RAS oncogene family	0.049576
AF095289	pituitary tumor-transforming 3, pseudogene	0.0478141
AF095844	interferon induced with helicase C domain 1	0.0263198
AF106069	ubiquitin specific peptidase 15	0.0181733
AF111849	ELOVL fatty acid elongase 5	0.0459686
AF112207	eukaryotic translation initiation factor 2B, subunit 4 delta, 67kDa	0.0034501
AF113216	platelet derived growth factor D	0.0136594
AF113701	ribosomal protein L22	0.0299676
AF116652	solute carrier family 16, member 10 (aromatic amino acid transporter)	0.0239796
AF116682	ABRA C-terminal like	0.0012007
AF117222	UDP-Gal:betaGlcNAc beta 1,3-galactosyltransferase, polypeptide 1	0.0382606
AF118124	myeloid cell leukemia sequence 1 (BCL2-related)	0.0011465
AF124366	family with sequence similarity 167, member A	0.0001437
AF129533	F-box and leucine-rich repeat protein 21 (gene/pseudogene)	0.0339957
AF130060	hemogen	0.0206326
AF130064	PRO2122	0.0161097
AF130079	uncharacterized protein PRO2852	0.0113374
AF131748	succinate-CoA ligase, GDP-forming, beta subunit	0.0164089
AF131803	transmembrane protein 109	0.0278291
AF131834	EFR3 homolog B (S. cerevisiae)	0.0257721
AF142063	oculomedin	0.0440669
AF143328	olfactory receptor, family 52, subfamily K, member 3 pseudogene	0.0344421
AF146277	CD2-associated protein	0.0260129
AF147302	iron-responsive element binding protein 2	0.0214376
AF147421	ARHGAP5 antisense RNA 1 (head to head)	0.0335801
AF151109	mitochondrial ribosomal protein L36	0.0288479
AF155065	ferric-chelate reductase 1-like	0.013455
AF155108	coiled-coil domain containing 34	0.0476382
AF155654	Morf4 family associated protein 1-like 1	0.041453

AF157323	F-box and leucine-rich repeat protein 5	0.0015548
AF157562	elongation factor RNA polymerase II	0.0236343
AF161383	coiled-coil domain containing 167	0.005426
AF161386	chromosome 7 open reading frame 55	0.0142475
AF161401	mitochondrial ribosomal protein L32	0.0232215
AF161436	aminoadipate-semialdehyde dehydrogenase	0.0054294
AF161437	glutamate-rich 1	0.021198
AF164438	neuronal PAS domain protein 3	0.0097574
AF174605	F-box protein 25	0.0346881
AF176012	DnaJ (Hsp40) homolog, subfamily C, member 12	0.0030256
AF177473	transient receptor potential cation channel, subfamily M, member 5	0.0334266
AF182417	mitochondrial ribosomal protein S21	0.0345693
AF182419	phosphopantothenoylcysteine decarboxylase	0.0331229
AF182422	mitochondrial ribosomal protein S35	0.0403493
AF204171	popeye domain containing 3	0.0400085
AF208232	interferon, alpha-inducible protein 27-like 2	0.0176017
AF209930	chordin	0.0481262
AF212247	integrin alpha FG-GAP repeat containing 1	0.0048638
AF215923	RAP1, GTP-GDP dissociation stimulator 1	0.0234971
AF217190	DEAH (Asp-Glu-Ala-His) box polypeptide 36	0.0321452
AF218008	major facilitator superfamily domain containing 12	0.0150637
AF221520	oligodendrocyte lineage transcription factor 2	0.0074036
AF222694	lectin, galactoside-binding, soluble, 12	0.0431068
AF222927	SAM domain, SH3 domain and nuclear localization signals 1	0.0328711
AF225422	MIF4G domain containing	0.0205733
AF230316	TSNAX-DISC1 readthrough	0.0245415
AF234532	myosin X	2.865E-05
AF235022	RAB38, member RAS oncogene family	0.0016188
AF235049	inhibitor of Bruton agammaglobulinemia tyrosine kinase	0.0141096
AF239156	peptide deformylase (mitochondrial)	0.0327351
AF244129	lymphocyte antigen 9	0.0364513
AF251025	zinc finger, FYVE domain containing 1	0.0258512
AF254085	POZ (BTB) and AT hook containing zinc finger 1	0.0009498

AF261758	24-dehydrocholesterol reductase	0.0018221
AF263541	dual-specificity tyrosine-(Y)-phosphorylation regulated kinase 4	0.0096071
AF264750	lysine (K)-specific methyltransferase 2C	0.0011528
AF270490	cysteine-rich, DPF motif domain containing 1///peroxisome proliferator-activated receptor alpha	0.0101516
AF275808	armadillo repeat containing 10	0.0427215
AF289485	chromosome 12 open reading frame 10	0.030089
AF290544	alanyl (membrane) aminopeptidase	0.0436329
AF294629	ALX homeobox 4	0.0247609
AF298152	B double prime 1, subunit of RNA polymerase III transcription initiation factor IIIB	0.0078244
AF302505	pellino E3 ubiquitin protein ligase 1	0.0080565
AF305836	DIO3 opposite strand/antisense RNA (head to head)	0.0439762
AF309653	membrane-spanning 4-domains, subfamily A, member 7	0.0454101
AF311287	caspase recruitment domain family, member 9	0.016128
AF317550	PERP, TP53 apoptosis effector	0.0116985
AJ007733	small nucleolar RNA, C/D box 7	0.0125403
AJ008144	mitogen-activated protein kinase kinase kinase 14	0.0031085
AJ011129	GLI pathogenesis-related 2	0.0257104
AJ011409	long intergenic non-protein coding RNA 527	0.046574
AJ011497	claudin 7	0.0157392
AJ132820	ADAM metallopeptidase domain 5, pseudogene	0.0471732
AJ133115	TSC22 domain family, member 4	0.0093275
AJ223366	myeloma overexpressed	0.0453399
AJ224326	ribulose-5-phosphate-3-epimerase	0.023737
AJ227859	egl nine homolog 1 (C. elegans)	0.0026718
AJ227863	muscleblind-like splicing regulator 1	0.0051571
AJ249902	SPARC related modular calcium binding 2	0.0161905
AJ249975	ankyrin repeat domain 2 (stretch responsive muscle)	0.0192858
AJ276207	solute carrier family 6 (proline IMINO transporter), member 20	0.0477935
AJ277276	transcriptional regulating factor 1	0.0039633
AJ299442	SIL1 homolog, endoplasmic reticulum chaperone (S. cerevisiae)	0.0040885
AJ300575	Z-DNA binding protein 1	1.06E-06
AK000004	FYVE, RhoGEF and PH domain containing 3	0.03351
AK000049	charged multivesicular body protein 4C	0.0415641

AK000102	zinc finger protein 280C	0.0216389
AK000229	chromosome 18 open reading frame 49	0.0287007
AK000315	alkB, alkylation repair homolog 5 (E. coli)	0.0290666
AK000529	phosphatidylinositol glycan anchor biosynthesis, class X	0.0462734
AK000660	cyclin-dependent kinase 6	0.0056173
AK000687	solute carrier family 6 (neutral amino acid transporter), member 19	0.0408085
AK000745	metadherin	0.0032416
AK000757	sortilin 1	0.0003361
AK000851	TBC1 domain family, member 22A	0.0210535
AK000933	choroideremia-like (Rab escort protein 2)	0.0057242
AK001036	SMAD family member 5	0.0108052
AK001052	fibroblast growth factor receptor 1	0.0057144
AK001069	UPF3 regulator of nonsense transcripts homolog A (yeast)	0.000469
AK001499	asunder spermatogenesis regulator	0.0022899
AK001814	uncharacterized LOC100505876	0.0008405
AK001843	family with sequence similarity 126, member B	0.0083869
AK001865	ERO1-like beta (S. cerevisiae)	0.0253627
AK001921	zinc finger, FYVE domain containing 21	0.0204381
AK001942	family with sequence similarity 49, member A	0.0216131
AK002107	RAB3B, member RAS oncogene family	0.0300077
AK021457	suppressor of Ty 20 homolog (S. cerevisiae)	0.000189
AK021510	phosphatidylinositol-4,5-bisphosphate 3-kinase, catalytic subunit alpha	0.0115611
AK021552	mir-100-let-7a-2 cluster host gene (non-protein coding)	0.008059
AK021571	ribosomal protein L35a	0.0284613
AK021607	essential meiotic endonuclease 1 homolog 1 (S. pombe)	0.0282439
AK021608	myotubularin related protein 14	0.0242441
AK021619	phosphatase and tensin homolog	0.0479826
AK021620	BMP2 inducible kinase	0.0290252
AK021639	chromosome X open reading frame 21	0.0012104
AK021691	caldesmon 1	0.0162357
AK021717	one cut homeobox 2	0.0344159
AK021737	hypoxia inducible factor 3, alpha subunit	0.0064198
AK021762	Ras and Rab interactor 3	0.039696

AK021770	cytochrome P450, family 20, subfamily A, polypeptide 1	0.0348749
AK021840	TBC1 domain family, member 22B	0.0004104
AK021852	dachsous 1 (Drosophila)	0.0051531
AK021874	transforming growth factor, beta 2	0.0480486
AK021876	solute carrier family 9, subfamily A (NHE5, cation proton antiporter 5), member 5	0.0073793
AK022005	eyes absent homolog 3 (Drosophila)	0.0343742
AK022050	heterogeneous nuclear ribonucleoprotein M	0.0210179
AK022144	tetraspanin 2	0.031695
AK022152	U2 small nuclear RNA auxiliary factor 1	0.0049115
AK022203	coiled-coil and C2 domain containing 2B	0.0093743
AK022215	neuroguidin, EIF4E binding protein	0.0372126
AK022255	chromosome 8 open reading frame 60	0.0257116
AK022260	uncharacterized LOC401052	0.0149004
AK022299	long intergenic non-protein coding RNA 662	0.0014045
AK022339	seryl-tRNA synthetase	0.0011881
AK022376	cyclin D3	0.0048049
AK022409	yippee-like 3 (Drosophila)	0.01424
AK022412	dedicator of cytokinesis 6	0.0161741
AK022418	long intergenic non-protein coding RNA 663	0.0065692
AK022478	SET binding factor 2	0.0498426
AK022487	unc-5 homolog C (C. elegans)	0.0161837
AK022500	migration and invasion inhibitory protein	0.0019969
AK022537	ribosome production factor 1 homolog (S. cerevisiae)	0.0131153
AK022551	fibrosin	0.0245064
AK022567	vasohibin 2	0.0272695
AK022628	forkhead box K1	0.0068573
AK022784	insulin-like growth factor 2 mRNA binding protein 1	0.0010872
AK022811	cytochrome b5 domain containing 1	0.0140154
AK022849	UTP15, U3 small nucleolar ribonucleoprotein, homolog (S. cerevisiae)	0.0252497
AK022850	tetratricopeptide repeat domain 31	0.0499019
AK023009	zinc finger protein 664	0.0445739
AK023035	WD repeat domain 76	0.0238877
AK023071	salvador homolog 1 (Drosophila)	0.0426202

AK023089	solute carrier family 30 (zinc transporter), member 7	0.027231
AK023114	NAD kinase	0.0036492
AK023237	Sel1 repeat containing 1	0.0170536
AK023371	PSMD6 antisense RNA 2	0.0290461
AK023400	membrane-associated ring finger (C3HC4) 7, E3 ubiquitin protein ligase	0.0100063
AK023435	GTPase, very large interferon inducible pseudogene 1	0.0050432
AK023472	coiled-coil domain containing 149	0.0231324
AK023557	core 1 synthase, glycoprotein-N-acetylgalactosamine 3-beta-galactosyltransferase, 1	0.0436695
AK023629	uncharacterized LOC100508120	0.0026011
AK023642	uncharacterized LOC388692	0.0350488
AK023724	helicase with zinc finger 2, transcriptional coactivator	0.017343
AK023749	growth regulation by estrogen in breast cancer-like	0.0356333
AK023755	triggering receptor expressed on myeloid cells-like 2	0.0228753
AK023759	CSRP2 binding protein	0.0269968
AK023803	ADP-ribosylation factor 1	0.0321494
AK023809	uncharacterized LOC284561	0.007508
AK023901	zinc finger protein 407	0.045072
AK023934	tubulin, gamma complex associated protein 5	0.0304698
AK023959	hydroxysteroid dehydrogenase like 2	0.0374059
AK023971	chromosome 16 open reading frame 59	0.0007482
AK023999	ankyrin repeat domain 33B	0.0020636
AK024046	methyltransferase like 8	0.0361499
AK024059	G protein-coupled receptor 180	0.0362115
AK024228	cyclin J-like	0.0025152
AK024270	kelch-like family member 24	0.0037105
AK024275	proline rich 5 like	0.038609
AK024326	aurora kinase A and ninein interacting protein	0.0417537
AK024327	RPTOR independent companion of MTOR, complex 2	0.00401
AK024331	senataxin	0.0008297
AK024341	ubiquitin specific peptidase 34	0.0242438
AK024423	autophagy related 16-like 2 (S. cerevisiae)	0.0126492
AK024480	intermediate filament family orphan 2	0.0121614
AK024484	WD repeat domain 5	0.0195881

AK024504	serine/threonine kinase 40	0.0326765
AK024551	transmembrane protein 204	0.0174258
AK024570	mitochondrial ribosomal protein L24	0.0397986
AK024637	zinc finger, DHHC-type containing 14	0.0107788
AK024639	ATPase type 13A3	0.0080308
AK024665	MUS81 structure-specific endonuclease	0.0207112
AK024722	CAP-GLY domain containing linker protein family, member 4	0.0295288
AK024734	methylthioadenosine phosphorylase	0.0006903
AK024747	HAUS augmin-like complex, subunit 1	0.0306499
AK024756	family with sequence similarity 118, member B	0.0108777
AK024779	chromosome 5 open reading frame 42	0.0070937
AK024821	suppressor of IKBKE 1	0.0069752
AK024889	laminin, alpha 3	0.0396034
AK024890	pyrin and HIN domain family, member 1	0.0122551
AK024913	coiled-coil domain containing 6	0.0019823
AK024953	cytochrome b561 family, member A3	0.0141828
AK024981	CREB regulated transcription coactivator 3	0.013719
AK025109	intraflagellar transport 80 homolog (Chlamydomonas)	0.0357286
AK025110	zinc finger protein 529	0.038058
AK025111	butyrophilin-like 8	0.0019177
AK025272	T-cell activation RhoGTPase activating protein	0.0002979
AK025329	ring finger protein 167	0.0371159
AK025343	hepatocellular carcinoma-related HCRP1	0.0346294
AK025345	leucine zipper protein 1	0.041416
AK025419	Spi-B transcription factor (Spi-1/PU.1 related)	0.0190048
AK025431	RELT-like 1	0.0178202
AK025442	biogenesis of lysosomal organelles complex-1, subunit 5, muted	0.0038289
AK025455	chromosome 14 open reading frame 169	0.0236392
AK025520	acyl-CoA binding domain containing 3	0.0032001
AK025589	StAR-related lipid transfer (START) domain containing 9	0.0407764
AK025615	branched chain amino-acid transaminase 1, cytosolic	0.0045714
AK025662	SHC SH2-domain binding protein 1	0.0097604
AK025758	nuclear factor of activated T-cells, cytoplasmic, calcineurin-dependent 2	0.0198953

AK025784	met proto-oncogene (hepatocyte growth factor receptor)	0.0014179
AK025798	VMA21 vacuolar H+ATPase homolog (S. cerevisiae)	0.0024807
AK025859	ankyrin repeat domain 17	0.0405192
AK025908	chromosome 12 open reading frame 65	0.0366727
AK025909	ephrin-A5	0.0398682
AK025943	NKF3 kinase family member	0.0488631
AK025953	myosin light chain kinase	0.0098177
AK025965	biorientation of chromosomes in cell division 1-like 1	0.0141465
AK025985	zinc finger and BTB domain containing 38	0.0145535
AK026022	chromodomain helicase DNA binding protein 6	0.003488
AK026068	CXADR-like membrane protein	0.0042883
AK026094	ubiquitin-conjugating enzyme E2H	0.0194914
AK026231	mitochondrial rRNA methyltransferase 1 homolog (S. cerevisiae)	0.029907
AK026266	fer-1-like 4 (C. elegans) pseudogene	0.0033107
AK026277	bora, aurora kinase A activator	7.122E-05
AK026288	ATH1, acid trehalase-like 1 (yeast)	0.0249682
AK026309	calcium/calmodulin-dependent protein kinase II gamma	0.0242754
AK026343	GTPase, IMAP family member 6	0.0463373
AK026366	zinc finger protein 677	0.0258161
AK026414	hexokinase domain containing 1	0.0397355
AK026486	nucleic acid binding protein 1	0.041802
AK026496	EF-hand domain (C-terminal) containing 2	0.0415915
AK026518	myosin XIX	0.0011204
AK026537	PHD finger protein 23	0.0402173
AK026583	chromosome 17 open reading frame 58	0.0109484
AK026684	coiled-coil domain containing 126	0.0023949
AK026776	leucine-rich repeat kinase 2	0.0200268
AK026813	STEAP family member 2, metalloreductase	0.0224293
AK026835	transcription factor B2, mitochondrial	0.017718
AK026860	ring finger protein 166	0.0198402
AK026873	uncharacterized LOC643837	0.0172896
AK026898	forkhead box P1	0.0338694
AK026958	coiled-coil domain containing 170	0.0371235

AK027028	transmembrane protein 62	0.0211783
AK027088	serine/threonine kinase 4	0.0405719
AK027094	asparaginyl-tRNA synthetase 2, mitochondrial (putative)	0.0017128
AK027161	tetratricopeptide repeat domain 12	0.038885
AK027180	myocyte enhancer factor 2D	0.0082549
AK027211	dynein, axonemal, heavy chain 3	0.0282177
AK027239	eukaryotic translation initiation factor 4E family member 2	0.0075886
AK027256	propionyl CoA carboxylase, alpha polypeptide	0.0413906
AL049296	polycomb group ring finger 1	0.0391937
AL049382	chromosome 12 open reading frame 29	0.00524
AL049437	dpy-19-like 2 pseudogene 2 (C. elegans)	0.0276428
AL049670	TIP41, TOR signaling pathway regulator-like (S. cerevisiae)	0.0189777
AL049675	uncharacterized LOC100506963	0.0058164
AL049705	mitochondrial ribosomal protein S14	0.0248119
AL049925	pygopus homolog 1 (Drosophila)	0.0290224
AL049943	family with sequence similarity 98, member A	0.0016888
AL049963	solute carrier family 39 (zinc transporter), member 8	0.0013232
AL049973	disrupted in schizophrenia 1	0.0356126
AL050137	olfactomedin-like 2B	0.035476
AL050288	autophagy related 4B, cysteine peptidase	0.0201203
AL050297	R3H domain and coiled-coil containing 1	0.0017397
AL050370	kelch-like family member 35	0.0008968
AL050371	phosphatidylserine decarboxylase	0.0020552
AL050389	putative homeodomain transcription factor 2	0.0048215
AL080062	methylmalonic aciduria (cobalamin deficiency) cblC type, with homocystinuria	0.018499
AL080065	family with sequence similarity 149, member A	0.0300776
AL080066	serine palmitoyltransferase, small subunit A	0.0042515
AL080085	solute carrier family 35, member E1	0.0197936
AL080110	progestin and adipoQ receptor family member III	0.0404459
AL080111	NIMA-related kinase 7	0.0103207
AL080133	spectrin repeat containing, nuclear envelope 2	0.0059571
AL080144	AT hook containing transcription factor 1	0.0212
AL080176	chromosome 1 open reading frame 204	0.0341834

AL080186	PNN-interacting serine/arginine-rich protein	0.0477209
AL080200	long intergenic non-protein coding RNA 588	0.0432093
AL110157	dual specificity phosphatase 7	0.0069655
AL110170	ribosomal protein L22-like 1	0.0249959
AL110185	up-regulated during skeletal muscle growth 5 homolog (mouse)	0.0258834
AL110194	high mobility group box 1	0.0467166
AL110202	transmembrane protein 123	0.0025348
AL110218	intraflagellar transport 172 homolog (Chlamydomonas)	0.0078467
AL110235	cannabinoid receptor interacting protein 1	0.0284804
AL110252	ganglioside induced differentiation associated protein 1	0.0386324
AL110257	family with sequence similarity 81, member A	0.0151493
AL117423	mitochondrial calcium uptake 1	0.0121703
AL117565	cysteine-serine-rich nuclear protein 1	0.0025279
AL117653	microphthalmia-associated transcription factor	0.0176618
AL122047	ARV1 homolog (S. cerevisiae)	0.0013178
AL133027	coiled-coil domain containing 136	0.0235289
AL133031	ligand dependent nuclear receptor corepressor-like	0.015596
AL133050	additional sex combs like 3 (Drosophila)	0.0356636
AL133057	leucine-rich repeats and WD repeat domain containing 1	0.0062236
AL133084	piggyBac transposable element derived 3	0.0239701
AL133087	ankyrin repeat domain 44	0.0087832
AL133090	ADAM metallopeptidase domain 22	0.0199171
AL133094	PHD finger protein 10	0.0061762
AL133101	centrosomal protein 85kDa-like	0.0276619
AL133108	zinc finger homeobox 3	0.0369351
AL133111	SH3BP5 antisense RNA 1	0.0116655
AL133602	F-box and leucine-rich repeat protein 17	0.0104208
AL137268	angel homolog 1 (Drosophila)	0.0012942
AL137270	long intergenic non-protein coding RNA 939	0.035037
AL137290	aryl hydrocarbon receptor nuclear translocator	0.0019113
AL137340	uncharacterized protein DKFZp761C1711	0.0441556
AL137382	solute carrier family 22, member 31	0.0470078
AL137430	uncharacterized LOC283070	0.0018446

AL137445	uncharacterized LOC401320	0.0071352
AL137479	two pore segment channel 2	0.0132652
AL137511	aarF domain containing kinase 3	0.0302467
AL137593	tripartite motif containing 41	0.0298592
AL137597	tumor protein p53 inducible nuclear protein 2	0.0101316
AL137602	zinc finger protein 44	0.0291376
AL137709	STARD13 antisense RNA	0.0131942
AL137727	transmembrane protein 55B	0.0188056
AL157442	glutamate receptor, ionotropic, N-methyl D-aspartate-associated protein 1 (glutamate binding)	0.0243857
AL157459	chromobox homolog 2	0.0401993
AL157468	CKLF-like MARVEL transmembrane domain containing 4	0.0154488
AL161992	MIT, microtubule interacting and transport, domain containing 1	0.0329203
AL162039	MOB kinase activator 1B	0.0396265
AL353933	solute carrier family 22, member 15	0.0339554
AL359591	transmembrane protein 55A	0.008011
AL359627	collagen, type XII, alpha 1	0.0342145
AL359943	uncharacterized LOC115110	0.0042019
AL360198	NPTN intronic transcript 1 (non-protein coding)	0.0284741
AL365410	myeloid/lymphoid or mixed-lineage leukemia (trithorax homolog, Drosophila); translocated to, 1	0.0050768
AL365413	CASP8 and FADD-like apoptosis regulator	0.0336262
AL365514	X-prolyl aminopeptidase (aminopeptidase P) 3, putative	0.0256962
AL390181	uncharacterized LOC642852	0.0003529
AY007163	UBX domain protein 11	0.0481151
AY008271	SWI/SNF-related, matrix-associated actin-dependent regulator of chromatin, subfamily a, containing D	0.041128
AY008301	MTERF domain containing 3	0.0496961
AY008372	oxysterol binding protein-like 3	0.0264035
AY009090	C-type lectin domain family 7, member A	0.0089878
AY009151	centromere protein K	0.0240733
AY009402	wingless-type MMTV integration site family, member 8A	0.0413764
AY010111	cadherin-related 23	0.0351477
D00265	cytochrome c, somatic	0.0153838
D13540	protein tyrosine phosphatase, non-receptor type 11	0.0067324
D13642	splicing factor 3b, subunit 3, 130kDa	0.0432641

D16888	C-terminal binding protein 2 pseudogene	0.0161921
D16892	heat shock protein 90kDa beta (Grp94), member 1	0.0016383
D17032	S-phase kinase-associated protein 1	0.0047988
D17093	solute carrier family 43, member 2	0.0292254
D17130	H3 histone, family 3A, pseudogene 4///H3 histone, family 3A	0.0053543
D17188	SENP3-EIF4A1 readthrough///small nucleolar RNA, H/ACA box 67///eukaryotic translation initiation fa	0.0018038
D26018	polymerase (DNA-directed), delta 3, accessory subunit	0.0170832
D26488	WD repeat domain 43	0.0442009
D28446	keratin 8	0.0163873
D28476	thyroid hormone receptor interactor 12	0.0026495
D29012	proteasome (prosome, macropain) subunit, beta type, 6	0.0054762
D29958	exosome component 7	0.0177137
D30612	zinc finger protein 282	0.0485503
D31885	ADP-ribosylation factor-like 6 interacting protein 1	0.0293983
D38438	postmeiotic segregation increased 2 pseudogene 4	0.0003725
D42044	ER membrane protein complex subunit 1	0.0475603
D43948	cytoskeleton associated protein 5	0.0068029
D50911	vestigial like 4 (Drosophila)	0.0434507
D83778	HMG box domain containing 3	0.0047824
D86980	tetratricopeptide repeat domain 9	0.0018506
D87450	wings apart-like homolog (Drosophila)	0.0171069
D87452	inositol hexakisphosphate kinase 1	0.0083694
D87454	kelch domain containing 10	0.0242154
K01900	interferon, alpha 8	0.0170419
L05500	adenylate cyclase 1 (brain)	0.0180609
L07383	small nucleolar RNA, H/ACA box 62	0.0176994
L10284	calnexin	9.692E-05
L13197	placenta-specific 4	0.0146874
L23867	TMLHE antisense RNA 1	0.0190102
L32537	keratin 18	0.0069793
L34070	NHP2 non-histone chromosome protein 2-like 1 (S. cerevisiae)	0.047012
L36587	small nucleolar RNA host gene 1 (non-protein coding)	0.0186963
L38517	indian hedgehog	0.0119231

L40392	RNA binding motif protein 25	0.0328753
L40992	runt-related transcription factor 2	0.0161494
L43345	uncharacterized LOC285628	0.0442446
M12807	CD4 molecule	0.0055734
M14200	diazepam binding inhibitor (GABA receptor modulator, acyl-CoA binding protein)	0.0005314
M15330	interleukin 1, beta	0.0386475
M17783	serpin peptidase inhibitor, clade E (nexin, plasminogen activator inhibitor type 1), member 2	0.0280964
M23161	multiple coagulation factor deficiency 2	0.006039
M26147	DNA nucleotidylexotransferase	0.0089526
M29540	carcinoembryonic antigen-related cell adhesion molecule 5	0.0371272
M30818	myxovirus (influenza virus) resistance 2 (mouse)	0.0006446
M34356	cAMP responsive element binding protein 1	0.0026218
M34671	CD59 molecule, complement regulatory protein	0.0147068
M37191	mitogen-activated protein kinase associated protein 1	0.0422362
M58050	CD46 molecule, complement regulatory protein	0.0178668
M58510	aconitase 1, soluble	0.0234349
M59040	CD44 molecule (Indian blood group)	0.0023288
M62403	insulin-like growth factor binding protein 4	0.0233578
M62896	annexin A2 pseudogene 1	0.0098121
M91368	solute carrier family 8 (sodium/calcium exchanger), member 1	0.0281528
M94547	myosin, light chain 7, regulatory	0.0382167
NM_000014	alpha-2-macroglobulin	0.0231493
NM_000016	acyl-CoA dehydrogenase, C-4 to C-12 straight chain	4.266E-05
NM_000019	acetyl-CoA acetyltransferase 1	0.0082299
NM_000022	adenosine deaminase	0.0494512
NM_000026	adenylosuccinate lyase	0.0111095
NM_000028	amylo-alpha-1, 6-glucosidase, 4-alpha-glucanotransferase	0.0022906
NM_000039	apolipoprotein A-I	0.0308114
NM_000040	apolipoprotein C-III	0.0207454
NM_000042	apolipoprotein H (beta-2-glycoprotein I)	0.0339354
NM_000054	arginine vasopressin receptor 2	0.0017908
NM_000066	complement component 8, beta polypeptide	0.0482173
NM_000074	CD40 ligand	0.0440622

NM_000085	chloride channel, voltage-sensitive Kb	0.0206553
NM_000092	collagen, type IV, alpha 4	0.0166951
NM_000104	cytochrome P450, family 1, subfamily B, polypeptide 1	0.0007777
NM_000108	dihydrolipoamide dehydrogenase	0.0154423
NM_000112	solute carrier family 26 (sulfate transporter), member 2	0.0059735
NM_000119	erythrocyte membrane protein band 4.2	0.0079364
NM_000129	coagulation factor XIII, A1 polypeptide	0.0129853
NM_000168	GLI family zinc finger 3	0.0080869
NM_000174	glycoprotein IX (platelet)	0.0333768
NM_000179	mutS homolog 6 (E. coli)	0.0166251
NM_000181	glucuronidase, beta	0.0002705
NM_000199	N-sulfoglucosamine sulfohydrolase	0.0006016
NM_000202	iduronate 2-sulfatase	0.0100171
NM_000254	5-methyltetrahydrofolate-homocysteine methyltransferase	0.0281701
NM_000260	myosin VIIA	0.0157226
NM_000292	phosphorylase kinase, alpha 2 (liver)	0.0173474
NM_000305	paraoxonase 2	0.01357
NM_000311	prion protein	0.000723
NM_000312	protein C (inactivator of coagulation factors Va and VIIIa)	0.0386548
NM_000313	protein S (alpha)	0.0023695
NM_000327	retinal outer segment membrane protein 1	0.0419621
NM_000328	retinitis pigmentosa GTPase regulator	0.0046536
NM_000329	retinal pigment epithelium-specific protein 65kDa	0.0237418
NM_000333	ataxin 7	0.0018945
NM_000361	thrombomodulin	0.0085694
NM_000363	troponin I type 3 (cardiac)	0.00651
NM_000372	tyrosinase	0.0004903
NM_000373	uridine monophosphate synthetase	0.0033927
NM_000382	aldehyde dehydrogenase 3 family, member A2	0.0046754
NM_000424	keratin 5	0.0487163
NM_000433	neutrophil cytosolic factor 2	0.0124458
NM_000434	sialidase 1 (lysosomal sialidase)	0.0489743
NM_000436	3-oxoacid CoA transferase 1	0.0027459

NM_000446	paraoxonase 1	0.0308854
NM_000454	superoxide dismutase 1, soluble	0.0012083
NM_000474	twist basic helix-loop-helix transcription factor 1	0.0186698
NM_000512	galactosamine (N-acetyl)-6-sulfate sulfatase	0.0308984
NM_000520	hexosaminidase A (alpha polypeptide)	0.00966
NM_000521	hexosaminidase B (beta polypeptide)	0.0097464
NM_000532	propionyl CoA carboxylase, beta polypeptide	0.0196609
NM_000544	transporter 2, ATP-binding cassette, sub-family B (MDR/TAP)	0.0117052
NM_000550	tyrosinase-related protein 1	0.0101273
NM_000557	growth differentiation factor 5	0.0366466
NM_000562	complement component 8, alpha polypeptide	0.0294882
NM_000565	interleukin 6 receptor	0.024908
NM_000579	chemokine (C-C motif) receptor 5 (gene/pseudogene)	0.0091379
NM_000591	CD14 molecule	0.0195518
NM_000598	insulin-like growth factor binding protein 3	0.0108087
NM_000655	selectin L	0.0129123
NM_000663	4-aminobutyrate aminotransferase	0.0053068
NM_000700	annexin A1	0.0005651
NM_000702	ATPase, Naسخ transporting, alpha 2 polypeptide	0.0076631
NM_000707	arginine vasopressin receptor 1B	0.0096065
NM_000712	biliverdin reductase A	0.0196802
NM_000721	calcium channel, voltage-dependent, R type, alpha 1E subunit	0.0203096
NM_000726	calcium channel, voltage-dependent, beta 4 subunit	0.006881
NM_000735	glycoprotein hormones, alpha polypeptide	0.0430207
NM_000744	cholinergic receptor, nicotinic, alpha 4 (neuronal)	0.0400836
NM_000759	colony stimulating factor 3 (granulocyte)	0.0070103
NM_000761	cytochrome P450, family 1, subfamily A, polypeptide 2	0.049054
NM_000786	cytochrome P450, family 51, subfamily A, polypeptide 1	0.0015838
NM_000788	deoxycytidine kinase	0.0229908
NM_000821	gamma-glutamyl carboxylase	0.0308967
NM_000849	glutathione S-transferase mu 3 (brain)	0.020336
NM_000854	glutathione S-transferase theta 2	0.0287843
NM_000857	guanylate cyclase 1, soluble, beta 3	0.0352915

NM_000860	hydroxyprostaglandin dehydrogenase 15-(NAD)	0.031541
NM_000873	intercellular adhesion molecule 2	0.0451429
NM_000896	cytochrome P450, family 4, subfamily F, polypeptide 3	0.0129942
NM_000903	NAD(P)H dehydrogenase, quinone 1	0.0221367
NM_000911	opioid receptor, delta 1	0.044203
NM_000917	prolyl 4-hydroxylase, alpha polypeptide I	0.0285877
NM_000920	pyruvate carboxylase	0.0216695
NM_000929	phospholipase A2, group V	0.0404338
NM_000935	procollagen-lysine, 2-oxoglutarate 5-dioxygenase 2	0.0055585
NM_000938	polymerase (RNA) II (DNA directed) polypeptide B, 140kDa	0.0039313
NM_000946	primase, DNA, polypeptide 1 (49kDa)	0.0051305
NM_000963	prostaglandin-endoperoxide synthase 2 (prostaglandin G/H synthase and cyclooxygenase)	0.000357
NM_000975	ribosomal protein L11	0.012386
NM_000979	ribosomal protein L18	0.0262738
NM_000981	ribosomal protein L19	0.0375177
NM_000985	ribosomal protein L17	0.0126716
NM_000988	ribosomal protein L27	0.0001839
NM_001000	ribosomal protein L39	0.0167538
NM_001001	ribosomal protein L36a-like	0.0155442
NM_001010	ribosomal protein S6	0.0110478
NM_001011	ribosomal protein S7	0.007095
NM_001022	ribosomal protein S19	0.0161604
NM_001025	ribosomal protein S23	0.0168293
NM_001048	somatostatin	0.0209473
NM_001050	somatostatin receptor 2	0.0350386
NM_001053	somatostatin receptor 5	0.0140004
NM_001066	tumor necrosis factor receptor superfamily, member 1B	0.0264549
NM_001085	serpin peptidase inhibitor, clade A (alpha-1 antiproteinase, antitrypsin), member 3	0.018527
NM_001099	acid phosphatase, prostate	0.0262542
NM_001102	actinin, alpha 1	0.0329874
NM_001107	acylphosphatase 1, erythrocyte (common) type	0.0002667
NM_001111	adenosine deaminase, RNA-specific	0.002862
NM_001117	adenylate cyclase activating polypeptide 1 (pituitary)	0.0085312

NM_001120	major facilitator superfamily domain containing 10	0.0011866
NM_001125	ADP-ribosylarginine hydrolase	0.0352843
NM_001134	alpha-fetoprotein	0.0128849
NM_001151	solute carrier family 25 (mitochondrial carrier; adenine nucleotide translocator), member 4	0.0091507
NM_001152	solute carrier family 25 (mitochondrial carrier; adenine nucleotide translocator), member 5	0.0048346
NM_001153	annexin A4	0.0457274
NM_001171	ATP-binding cassette, sub-family C (CFTR/MRP), member 6	0.032484
NM_001177	ADP-ribosylation factor-like 1	0.0081855
NM_001178	aryl hydrocarbon receptor nuclear translocator-like	0.0057641
NM_001196	BH3 interacting domain death agonist	0.0154481
NM_001216	carbonic anhydrase IX	0.0352931
NM_001225	caspase 4, apoptosis-related cysteine peptidase	0.0059209
NM_001228	caspase 8, apoptosis-related cysteine peptidase	0.0044598
NM_001244	tumor necrosis factor (ligand) superfamily, member 8	0.0026064
NM_001258	cyclin-dependent kinase 3	0.0349836
NM_001262	cyclin-dependent kinase inhibitor 2C (p18, inhibits CDK4)	0.0061641
NM_001267	chondroadherin	0.0434516
NM_001276	chitinase 3-like 1 (cartilage glycoprotein-39)	0.0000317
NM_001281	tubulin folding cofactor B	0.0471029
NM_001282	adaptor-related protein complex 2, beta 1 subunit	0.0058692
NM_001288	chloride intracellular channel 1	0.0001742
NM_001299	calponin 1, basic, smooth muscle	0.037206
NM_001321	cysteine and glycine-rich protein 2	0.0298906
NM_001326	cleavage stimulation factor, 3' pre-RNA, subunit 3, 77kDa	0.022332
NM_001338	coxsackie virus and adenovirus receptor	0.014269
NM_001343	Dab, mitogen-responsive phosphoprotein, homolog 2 (Drosophila)	0.0287736
NM_001353	aldo-keto reductase family 1, member C1	0.0338712
NM_001357	DEAH (Asp-Glu-Ala-His) box helicase 9	0.048269
NM_001365	discs, large homolog 4 (Drosophila)	0.0485692
NM_001394	dual specificity phosphatase 4	0.0220641
NM_001397	endothelin converting enzyme 1	0.0017175
NM_001402	eukaryotic translation elongation factor 1 alpha 1	0.0224251
NM_001424	epithelial membrane protein 2	0.0330591

NM_001441	fatty acid amide hydrolase	0.0295011
NM_001452	forkhead box F2	0.0052262
NM_001454	forkhead box J1	0.0378676
NM_001455	forkhead box O3	0.0340319
NM_001467	solute carrier family 37 (glucose-6-phosphate transporter), member 4	0.0398764
NM_001478	beta-1,4-N-acetyl-galactosaminyl transferase 1	0.026636
NM_001487	biogenesis of lysosomal organelles complex-1, subunit 1	0.0018087
NM_001493	GDP dissociation inhibitor 1	0.0355137
NM_001513	glutathione S-transferase zeta 1	0.0244925
NM_001533	heterogeneous nuclear ribonucleoprotein L	0.0293455
NM_001545	immature colon carcinoma transcript 1	0.0048615
NM_001548	interferon-induced protein with tetratricopeptide repeats 1	0.0015792
NM_001550	interferon-related developmental regulator 1	0.0057053
NM_001551	immunoglobulin (CD79A) binding protein 1	0.0333872
NM_001553	insulin-like growth factor binding protein 7	0.0024304
NM_001561	tumor necrosis factor receptor superfamily, member 9	0.0061689
NM_001610	acid phosphatase 2, lysosomal	0.0087199
NM_001614	actin, gamma 1	0.0107996
NM_001627	activated leukocyte cell adhesion molecule	0.0035335
NM_001673	asparagine synthetase (glutamine-hydrolyzing)	0.0267012
NM_001685	ATP synthase, H transporting, mitochondrial Fo complex, subunit F6	0.0297194
NM_001688	ATP synthase, H transporting, mitochondrial Fo complex, subunit B1	0.0290307
NM_001699	AXL receptor tyrosine kinase	0.0426547
NM_001703	brain-specific angiogenesis inhibitor 2	0.0495083
NM_001714	bicaudal D homolog 1 (Drosophila)	0.0447987
NM_001719	bone morphogenetic protein 7	0.0332961
NM_001735	complement component 5	0.0156738
NM_001740	calbindin 2	0.0237664
NM_001764	CD1b molecule	0.0058402
NM_001780	CD63 molecule	0.0051809
NM_001786	cyclin-dependent kinase 1	0.0243673
NM_001806	CCAAT/enhancer binding protein (C/EBP), gamma	0.0107263
NM_001814	cathepsin C	0.0002078

NM_001815	carcinoembryonic antigen-related cell adhesion molecule 3	0.0332669
NM_001823	creatine kinase, brain	0.0477622
NM_001824	creatine kinase, muscle	0.0379246
NM_001827	CDC28 protein kinase regulatory subunit 2	0.0023557
NM_001838	chemokine (C-C motif) receptor 7	0.0400485
NM_001854	collagen, type XI, alpha 1	0.0068509
NM_001862	cytochrome c oxidase subunit Vb	0.0152475
NM_001863	cytochrome c oxidase subunit VIb polypeptide 1 (ubiquitous)	0.0348046
NM_001866	cytochrome c oxidase subunit VIIb	0.0195899
NM_001867	cytochrome c oxidase subunit VIIc	0.0497821
NM_001875	carbamoyl-phosphate synthase 1, mitochondrial	0.0288129
NM_001876	carnitine palmitoyltransferase 1A (liver)	0.0164297
NM_001887	crystallin, beta B1	0.0022101
NM_001889	crystallin, zeta (quinone reductase)	0.0018931
NM_001896	casein kinase 2, alpha prime polypeptide	0.0194907
NM_001912	cathepsin L1	0.0048624
NM_001924	growth arrest and DNA-damage-inducible, alpha	0.0013208
NM_001935	dipeptidyl-peptidase 4	0.0003056
NM_001943	desmoglein 2	0.0419528
NM_001946	dual specificity phosphatase 6	0.038371
NM_001949	E2F transcription factor 3	0.0371865
NM_001955	endothelin 1	0.0382292
NM_001958	eukaryotic translation elongation factor 1 alpha 2	0.0032344
NM_001966	enoyl-CoA, hydratase/3-hydroxyacyl CoA dehydrogenase	0.008869
NM_001975	enolase 2 (gamma, neuronal)	0.0450932
NM_001981	epidermal growth factor receptor pathway substrate 15	0.0240145
NM_001995	acyl-CoA synthetase long-chain family member 1	0.0043983
NM_002006	fibroblast growth factor 2 (basic)	0.0010095
NM_002013	FK506 binding protein 3, 25kDa	0.0011779
NM_002014	FK506 binding protein 4, 59kDa	0.0003906
NM_002017	Friend leukemia virus integration 1	0.0308147
NM_002019	fms-related tyrosine kinase 1	0.0112364
NM_002029	formyl peptide receptor 1	0.0042797

NM_002037	FYN oncogene related to SRC, FGR, YES	0.0042682
NM_002038	interferon, alpha-inducible protein 6	0.0094437
NM_002046	glyceraldehyde-3-phosphate dehydrogenase	0.0174247
NM_002047	glycyl-tRNA synthetase	0.0092578
NM_002079	glutamic-oxaloacetic transaminase 1, soluble	0.0379407
NM_002094	G1 to S phase transition 1	0.0399078
NM_002107	H3 histone, family 3A	0.0082594
NM_002108	histidine ammonia-lyase	0.0193271
NM_002109	histidyl-tRNA synthetase	0.0059827
NM_002114	human immunodeficiency virus type I enhancer binding protein 1	0.0344004
NM_002145	homeobox B2	0.0449741
NM_002149	hippocalcin-like 1	0.0161117
NM_002151	hepsin	0.0239551
NM_002155	heat shock 70kDa protein 6 (HSP70B')	0.003524
NM_002156	heat shock 60kDa protein 1 (chaperonin)	0.0169043
NM_002162	intercellular adhesion molecule 3	0.0048425
NM_002169	interferon, alpha 5	0.0103121
NM_002184	interleukin 6 signal transducer (gp130, oncostatin M receptor)	0.0197131
NM_002198	interferon regulatory factor 1	0.0094819
NM_002205	integrin, alpha 5 (fibronectin receptor, alpha polypeptide)	0.0021903
NM_002206	integrin, alpha 7	0.0001802
NM_002208	integrin, alpha E (antigen CD103, human mucosal lymphocyte antigen 1; alpha polypeptide)	0.00433
NM_002212	eukaryotic translation initiation factor 6	0.0450371
NM_002214	integrin, beta 8	0.0282997
NM_002216	inter-alpha-trypsin inhibitor heavy chain 2	0.0069719
NM_002224	inositol 1,4,5-trisphosphate receptor, type 3	0.0080614
NM_002243	potassium inwardly-rectifying channel, subfamily J, member 15	0.0317603
NM_002248	potassium intermediate/small conductance calcium-activated channel, subfamily N, member 1	0.0122406
NM_002254	kinesin family member 3C	0.0147117
NM_002266	karyopherin alpha 2 (RAG cohort 1, importin alpha 1)	0.0092723
NM_002271	importin 5	0.0145347
NM_002276	keratin 19	0.023743
NM_002287	leukocyte-associated immunoglobulin-like receptor 1	0.0004716

NM_002296	lamin B receptor	0.0191355
NM_002305	lectin, galactoside-binding, soluble, 1	0.0347277
NM_002337	low density lipoprotein receptor-related protein associated protein 1	0.0001814
NM_002356	myristoylated alanine-rich protein kinase C substrate	0.0387402
NM_002357	MAX dimerization protein 1	0.0004055
NM_002359	v-maf musculoaponeurotic fibrosarcoma oncogene homolog G (avian)	0.0453054
NM_002367	melanoma antigen family B, 4	0.0467051
NM_002371	mal, T-cell differentiation protein	0.0032869
NM_002374	microtubule-associated protein 2	0.0399695
NM_002385	myelin basic protein	0.0082899
NM_002414	CD99 molecule	0.0286106
NM_002434	N-methylpurine-DNA glycosylase	0.0235697
NM_002442	musashi RNA-binding protein 1	0.0118434
NM_002448	msh homeobox 1	0.040628
NM_002450	metallothionein 1L (gene/pseudogene)	0.0071753
NM_002452	nudix (nucleoside diphosphate linked moiety X)-type motif 1	0.0199005
NM_002460	interferon regulatory factor 4	0.0307994
NM_002462	myxovirus (influenza virus) resistance 1, interferon-inducible protein p78 (mouse)	0.0016613
NM_002468	myeloid differentiation primary response 88	0.0310446
NM_002477	myosin, light chain 5, regulatory	0.0415498
NM_002480	protein phosphatase 1, regulatory subunit 12A	0.0075975
NM_002486	nuclear cap binding protein subunit 1, 80kDa	0.0432423
NM_002487	necdin, melanoma antigen (MAGE) family member	0.0121074
NM_002488	NADH dehydrogenase (ubiquinone) 1 alpha subcomplex, 2, 8kDa	0.0041703
NM_002490	NADH dehydrogenase (ubiquinone) 1 alpha subcomplex, 6, 14kDa	0.0134241
NM_002495	NADH dehydrogenase (ubiquinone) Fe-S protein 4, 18kDa (NADH-coenzyme Q reductase)	0.0082337
NM_002510	glycoprotein (transmembrane) nmb	0.0351305
NM_002526	5'-nucleotidase, ecto (CD73)	0.0110058
NM_002546	tumor necrosis factor receptor superfamily, member 11b	0.0008295
NM_002557	oviductal glycoprotein 1, 120kDa	0.0282076
NM_002567	phosphatidylethanolamine binding protein 1	0.029558
NM_002571	progestagen-associated endometrial protein	0.0176775
NM_002574	peroxiredoxin 1	0.0177763

NM_002575	serpin peptidase inhibitor, clade B (ovalbumin), member 2	0.0254197
NM_002586	pre-B-cell leukemia homeobox 2	0.0096976
NM_002591	phosphoenolpyruvate carboxykinase 1 (soluble)	0.0046883
NM_002592	proliferating cell nuclear antigen	0.0355453
NM_002598	programmed cell death 2	0.0262436
NM_002615	serpin peptidase inhibitor, clade F (alpha-2 antiplasmin, pigment epithelium derived factor), member [illegible]	0.0305058
NM_002626	phosphofructokinase, liver	0.0208708
NM_002628	profilin 2	0.0011953
NM_002635	solute carrier family 25 (mitochondrial carrier; phosphate carrier), member 3	0.0088631
NM_002636	PHD finger protein 1	0.0280485
NM_002643	phosphatidylinositol glycan anchor biosynthesis, class F	0.031251
NM_002655	pleiomorphic adenoma gene 1	0.0195041
NM_002668	proteolipid protein 2 (colonic epithelium-enriched)	0.0210954
NM_002685	exosome component 10	0.0177597
NM_002687	pinin, desmosome associated protein	0.0443305
NM_002696	polymerase (RNA) II (DNA directed) polypeptide G	0.0090742
NM_002714	protein phosphatase 1, regulatory subunit 10	0.0120266
NM_002722	pancreatic polypeptide	0.0213719
NM_002729	hematopoietically expressed homeobox	0.006722
NM_002737	protein kinase C, alpha	0.0030193
NM_002759	eukaryotic translation initiation factor 2-alpha kinase 2	0.019649
NM_002770	protease, serine, 2 (trypsin 2)	0.0251687
NM_002786	proteasome (prosome, macropain) subunit, alpha type, 1	0.0414641
NM_002787	proteasome (prosome, macropain) subunit, alpha type, 2	0.042066
NM_002789	proteasome (prosome, macropain) subunit, alpha type, 4	0.0091962
NM_002790	proteasome (prosome, macropain) subunit, alpha type, 5	0.0036538
NM_002795	proteasome (prosome, macropain) subunit, beta type, 3	0.036422
NM_002797	proteasome (prosome, macropain) subunit, beta type, 5	0.0359264
NM_002799	proteasome (prosome, macropain) subunit, beta type, 7	0.0003797
NM_002803	proteasome (prosome, macropain) 26S subunit, ATPase, 2	0.0038551
NM_002805	proteasome (prosome, macropain) 26S subunit, ATPase, 5	0.0343659
NM_002807	proteasome (prosome, macropain) 26S subunit, non-ATPase, 1	0.001747
NM_002813	proteasome (prosome, macropain) 26S subunit, non-ATPase, 9	0.0288718

NM_002822	twinfilin actin-binding protein 1	0.0164899
NM_002827	protein tyrosine phosphatase, non-receptor type 1	0.0012928
NM_002829	protein tyrosine phosphatase, non-receptor type 3	0.0236259
NM_002831	protein tyrosine phosphatase, non-receptor type 6	0.0030347
NM_002836	protein tyrosine phosphatase, receptor type, A	0.0333356
NM_002837	protein tyrosine phosphatase, receptor type, B	0.0131349
NM_002880	v-raf-1 murine leukemia viral oncogene homolog 1	0.0009059
NM_002892	AT rich interactive domain 4A (RBP1-like)	0.0332234
NM_002900	retinol binding protein 3, interstitial	0.0200897
NM_002913	replication factor C (activator 1) 1, 145kDa	0.0115218
NM_002916	replication factor C (activator 1) 4, 37kDa	0.0215667
NM_002922	regulator of G-protein signaling 1	0.0019595
NM_002939	ribonuclease/angiogenin inhibitor 1	0.0475895
NM_002947	replication protein A3, 14kDa	0.0357823
NM_002948	ribosomal protein L15	0.0349911
NM_002949	mitochondrial ribosomal protein L12	0.025407
NM_002954	ribosomal protein S27a	0.014489
NM_002979	sterol carrier protein 2	0.010593
NM_002990	chemokine (C-C motif) ligand 22	0.0175945
NM_002993	chemokine (C-X-C motif) ligand 6	0.048633
NM_003003	SEC14-like 1 (S. cerevisiae)	0.021814
NM_003033	ST3 beta-galactoside alpha-2,3-sialyltransferase 1	0.0123414
NM_003044	solute carrier family 6 (neurotransmitter transporter, betaine/GABA), member 12	0.0208326
NM_003049	solute carrier family 10 (sodium/bile acid cotransporter family), member 1	0.0066459
NM_003057	solute carrier family 22 (organic cation transporter), member 1	0.001327
NM_003060	solute carrier family 22 (organic cation/carnitine transporter), member 5	0.0149435
NM_003069	SWI/SNF related, matrix associated, actin dependent regulator of chromatin, subfamily a, member 1	0.0384535
NM_003075	SWI/SNF related, matrix associated, actin dependent regulator of chromatin, subfamily c, member 2	0.0108455
NM_003090	small nuclear ribonucleoprotein polypeptide A'	0.0099226
NM_003092	small nuclear ribonucleoprotein polypeptide B	0.020734
NM_003093	small nuclear ribonucleoprotein polypeptide C	0.0147414
NM_003100	sorting nexin 2	0.006421
NM_003115	UDP-N-acteylglucosamine pyrophosphorylase 1	0.0007524

NM_003118	secreted protein, acidic, cysteine-rich (osteonectin)	0.0453108
NM_003131	serum response factor (c-fos serum response element-binding transcription factor)	0.0133903
NM_003134	signal recognition particle 14kDa (homologous Alu RNA binding protein)	0.0107585
NM_003143	single-stranded DNA binding protein 1, mitochondrial	0.0134828
NM_003144	signal sequence receptor, alpha	0.0395614
NM_003146	structure specific recognition protein 1	0.0147824
NM_003149	SH3 and cysteine rich domain	0.0422869
NM_003161	ribosomal protein S6 kinase, 70kDa, polypeptide 1	0.0453555
NM_003165	syntaxin binding protein 1	0.0154004
NM_003193	tubulin folding cofactor E	0.0402081
NM_003234	transferrin receptor (p90, CD71)	0.0054883
NM_003240	left-right determination factor 2	0.0268641
NM_003248	thrombospondin 4	0.0241757
NM_003254	TIMP metallopeptidase inhibitor 1	0.0384921
NM_003276	thymopoietin	0.0151223
NM_003318	TTK protein kinase	0.0371636
NM_003323	tubby like protein 2	0.0320669
NM_003338	ubiquitin-conjugating enzyme E2D 1	0.0043066
NM_003340	ubiquitin-conjugating enzyme E2D 3	0.0224883
NM_003348	ubiquitin-conjugating enzyme E2N	0.02603
NM_003364	uridine phosphorylase 1	0.0035229
NM_003365	ubiquinol-cytochrome c reductase core protein I	0.0322976
NM_003366	ubiquinol-cytochrome c reductase core protein II	0.0057122
NM_003370	vasodilator-stimulated phosphoprotein	0.0148408
NM_003377	vascular endothelial growth factor B	0.0002125
NM_003384	vaccinia related kinase 1	0.0002626
NM_003407	ZFP36 ring finger protein	0.0010989
NM_003425	zinc finger protein 45	0.0330284
NM_003426	zinc finger protein 74	0.0368398
NM_003434	zinc finger protein 133	0.01142
NM_003454	zinc finger protein 200	0.0371016
NM_003456	zinc finger protein 205	0.0194696
NM_003457	zinc finger protein 207	0.005417

NM_003465	chitinase 1 (chitotriosidase)	0.041634
NM_003467	chemokine (C-X-C motif) receptor 4	0.0029017
NM_003470	ubiquitin specific peptidase 7 (herpes virus-associated)	0.004247
NM_003474	ADAM metallopeptidase domain 12	0.044287
NM_003478	cullin 5	0.0108896
NM_003488	A kinase (PRKA) anchor protein 1	0.0362302
NM_003495	histone cluster 1, H4i	0.0272354
NM_003498	stannin	0.0432592
NM_003509	histone cluster 1, H2ai	0.006263
NM_003529	histone cluster 1, H3a	0.0438443
NM_003531	histone cluster 1, H3c	0.0427608
NM_003542	histone cluster 1, H4c	0.0154394
NM_003543	histone cluster 1, H4h	0.0006819
NM_003549	hyaluronoglucosaminidase 3	0.0449431
NM_003564	transgelin 2	0.0325041
NM_003566	early endosome antigen 1	0.0460287
NM_003581	NCK adaptor protein 2	0.0199283
NM_003591	cullin 2	0.0431851
NM_003594	transcription termination factor, RNA polymerase II	0.0186976
NM_003595	tyrosylprotein sulfotransferase 2	0.0142109
NM_003599	suppressor of Ty 3 homolog (S. cerevisiae)	0.0013385
NM_003608	G protein-coupled receptor 65	0.00225
NM_003626	protein tyrosine phosphatase, receptor type, f polypeptide (PTPRF), interacting protein (liprin), alpha 1	0.0239102
NM_003632	contactin associated protein 1	0.0485671
NM_003633	ectodermal-neural cortex 1 (with BTB domain)	0.0158284
NM_003639	inhibitor of kappa light polypeptide gene enhancer in B-cells, kinase gamma	0.0215034
NM_003685	KH-type splicing regulatory protein	0.0195413
NM_003690	protein kinase, interferon-inducible double stranded RNA dependent activator	0.031463
NM_003693	scavenger receptor class F, member 1	0.0442641
NM_003704	family with sequence similarity 193, member A	0.0042182
NM_003705	solute carrier family 25 (aspartate/glutamate carrier), member 12	0.0093859
NM_003720	proteasome (prosome, macropain) assembly chaperone 1	0.0355269
NM_003756	eukaryotic translation initiation factor 3, subunit H	0.0478775

NM_003765	syntaxin 10	0.0095909
NM_003770	keratin 37	0.0358062
NM_003776	mitochondrial ribosomal protein L40	0.0340952
NM_003784	serpin peptidase inhibitor, clade B (ovalbumin), member 7	0.0026836
NM_003796	URI1, prefoldin-like chaperone	0.0228682
NM_003804	receptor (TNFRSF)-interacting serine-threonine kinase 1	0.008694
NM_003822	nuclear receptor subfamily 5, group A, member 2	0.0485421
NM_003827	N-ethylmaleimide-sensitive factor attachment protein, alpha	0.0070229
NM_003830	sialic acid binding Ig-like lectin 5	0.0463954
NM_003837	fructose-1,6-bisphosphatase 2	0.0497364
NM_003846	peroxisomal biogenesis factor 11 beta	0.0170383
NM_003865	HESX homeobox 1	0.0453597
NM_003885	cyclin-dependent kinase 5, regulatory subunit 1 (p35)	0.030482
NM_003905	NEDD8 activating enzyme E1 subunit 1	0.0001344
NM_003908	eukaryotic translation initiation factor 2, subunit 2 beta, 38kDa	0.0082806
NM_003909	copine III	0.0071253
NM_003933	BAI1-associated protein 3	0.0298786
NM_003937	kynureninase	0.0404848
NM_003940	ubiquitin specific peptidase 13 (isopeptidase T-3)	0.0360831
NM_004031	interferon regulatory factor 7	0.0156628
NM_004035	acyl-CoA oxidase 1, palmitoyl	0.0448314
NM_004039	annexin A2	0.0462023
NM_004042	arylsulfatase F	0.0093337
NM_004044	5-aminoimidazole-4-carboxamide ribonucleotide formyltransferase/IMP cyclohydrolase	0.0450955
NM_004045	antioxidant 1 copper chaperone	0.0010633
NM_004046	ATP synthase, H transporting, mitochondrial F1 complex, alpha subunit 1, cardiac muscle	0.016741
NM_004049	BCL2-related protein A1	0.00335
NM_004071	CDC-like kinase 1	0.0173725
NM_004072	chemokine-like receptor 1	0.0331508
NM_004074	cytochrome c oxidase subunit VIIIA (ubiquitous)	0.0217366
NM_004076	crystallin, beta B3	0.01337
NM_004079	cathepsin S	0.0048471
NM_004090	dual specificity phosphatase 3	0.0144295

NM_004115	fibroblast growth factor 14	0.0333035
NM_004117	FK506 binding protein 5	0.0047565
NM_004128	general transcription factor IIF, polypeptide 2, 30kDa	0.0442256
NM_004130	glycogenin 1	0.0441453
NM_004146	NADH dehydrogenase (ubiquinone) 1 beta subcomplex, 7, 18kDa	0.030935
NM_004152	ornithine decarboxylase antizyme 1	0.0189854
NM_004157	protein kinase, cAMP-dependent, regulatory, type II, alpha	0.0162813
NM_004164	retinol binding protein 2, cellular	0.022695
NM_004165	Ras-related associated with diabetes	0.0370929
NM_004173	solute carrier family 7 (orphan transporter), member 4	0.0050728
NM_004182	ubiquitously-expressed, prefoldin-like chaperone	0.0098496
NM_004186	sema domain, immunoglobulin domain (Ig), short basic domain, secreted, (semaphorin) 3F	0.0050537
NM_004190	lipase, gastric	0.0001149
NM_004199	prolyl 4-hydroxylase, alpha polypeptide II	0.0083313
NM_004214	fibroblast growth factor (acidic) intracellular binding protein	0.0457819
NM_004221	interleukin 32	0.0259513
NM_004226	serine/threonine kinase 17b	0.0162833
NM_004236	COP9 signalosome subunit 2	0.0056896
NM_004251	RAB9A, member RAS oncogene family	0.0485874
NM_004270	mediator complex subunit 7	0.00079
NM_004271	lymphocyte antigen 86	0.0098206
NM_004279	peptidase (mitochondrial processing) beta	0.0393945
NM_004280	eukaryotic translation elongation factor 1 epsilon 1	0.0120831
NM_004282	BCL2-associated athanogene 2	0.0139986
NM_004293	guanine deaminase	0.0226961
NM_004311	ADP-ribosylation factor-like 3	0.0052848
NM_004318	aspartate beta-hydroxylase	0.0266634
NM_004331	BCL2/adenovirus E1B 19kDa interacting protein 3-like	1.605E-05
NM_004337	oxidative stress induced growth inhibitor family member 2	3.206E-05
NM_004341	carbamoyl-phosphate synthetase 2, aspartate transcarbamylase, and dihydroorotase	0.0458932
NM_004349	runt-related transcription factor 1; translocated to, 1 (cyclin D-related)	0.0401047
NM_004362	calmegin	0.0310363
NM_004367	chemokine (C-C motif) receptor 6	0.0218042

NM_004415	desmoplakin	0.0091345
NM_004430	early growth response 3	0.0004722
NM_004440	EPH receptor A7	0.0492519
NM_004450	enhancer of rudimentary homolog (Drosophila)	0.0032759
NM_004451	estrogen-related receptor alpha	0.0105247
NM_004470	FK506 binding protein 2, 13kDa	0.010927
NM_004472	forkhead box D1	0.0120175
NM_004480	fucosyltransferase 8 (alpha (1,6) fucosyltransferase)	0.0039177
NM_004483	glycine cleavage system protein H (aminomethyl carrier)	0.0169726
NM_004515	interleukin enhancer binding factor 2	0.0449539
NM_004519	potassium voltage-gated channel, KQT-like subfamily, member 3	0.0088812
NM_004520	kinesin heavy chain member 2A	0.0180072
NM_004526	minichromosome maintenance complex component 2	0.0333426
NM_004541	NADH dehydrogenase (ubiquinone) 1 alpha subcomplex, 1, 7.5kDa	0.0192004
NM_004542	NADH dehydrogenase (ubiquinone) 1 alpha subcomplex, 3, 9kDa	0.0449045
NM_004546	NADH dehydrogenase (ubiquinone) 1 beta subcomplex, 2, 8kDa	0.0003724
NM_004549	NADH dehydrogenase (ubiquinone) 1, subcomplex unknown, 2, 14.5kDa	0.0001079
NM_004552	NADH dehydrogenase (ubiquinone) Fe-S protein 5, 15kDa (NADH-coenzyme Q reductase)	0.0023383
NM_004553	NADH dehydrogenase (ubiquinone) Fe-S protein 6, 13kDa (NADH-coenzyme Q reductase)	0.0152045
NM_004564	PET112 homolog (yeast)	0.0259115
NM_004568	serpin peptidase inhibitor, clade B (ovalbumin), member 6	0.0045898
NM_004572	plakophilin 2	0.0097631
NM_004573	phospholipase C, beta 2	0.0467621
NM_004582	Rab geranylgeranyltransferase, beta subunit	0.0052681
NM_004587	ribosome binding protein 1	0.0092638
NM_004589	SCO1 cytochrome c oxidase assembly protein	0.0395257
NM_004598	sparc/osteonectin, cwcv and kazal-like domains proteoglycan (testican) 1	0.0111925
NM_004607	tubulin folding cofactor A	0.0378806
NM_004612	transforming growth factor, beta receptor 1	0.0411195
NM_004666	vanin 1	0.047199
NM_004672	mitogen-activated protein kinase kinase kinase 6	0.0147351
NM_004689	metastasis associated 1	0.0330842
NM_004693	keratin 75	0.0235369

NM_004717	diacylglycerol kinase, iota	0.0413571
NM_004730	eukaryotic translation termination factor 1	0.0306389
NM_004749	transforming growth factor beta regulator 4	0.0109978
NM_004750	cytokine receptor-like factor 1	0.0171075
NM_004751	glucosaminyl (N-acetyl) transferase 3, mucin type	0.0316493
NM_004755	ribosomal protein S6 kinase, 90kDa, polypeptide 5	0.0084857
NM_004757	aminoacyl tRNA synthetase complex-interacting multifunctional protein 1	0.0053534
NM_004765	B-cell CLL/lymphoma 7C	0.0493001
NM_004766	coatomer protein complex, subunit beta 2 (beta prime)	0.0400037
NM_004774	mediator complex subunit 1	0.0096902
NM_004792	peptidylprolyl isomerase G (cyclophilin G)	0.0268786
NM_004796	neurexin 3	0.047895
NM_004807	heparan sulfate 6-O-sulfotransferase 1	0.0215296
NM_004822	netrin 1	0.0342099
NM_004824	chromodomain protein, Y-like	0.0310964
NM_004839	homer homolog 2 (Drosophila)	0.0020191
NM_004850	Rho-associated, coiled-coil containing protein kinase 2	0.0388574
NM_004859	clathrin, heavy chain (Hc)	0.0192354
NM_004862	lipopolysaccharide-induced TNF factor	0.0007707
NM_004866	secretory carrier membrane protein 1	0.0027106
NM_004867	integral membrane protein 2A	0.0076242
NM_004879	etoposide induced 2.4	0.0258033
NM_004887	chemokine (C-X-C motif) ligand 14	0.0453976
NM_004889	ATP synthase, H transporting, mitochondrial Fo complex, subunit F2	0.021667
NM_004891	mitochondrial ribosomal protein L33	0.0047986
NM_004894	chromosome 14 open reading frame 2	0.0071318
NM_004895	NLR family, pyrin domain containing 3	0.0007006
NM_004915	ATP-binding cassette, sub-family G (WHITE), member 1	0.0007788
NM_004939	DEAD (Asp-Glu-Ala-Asp) box helicase 1	0.0101445
NM_004959	nuclear receptor subfamily 5, group A, member 1	0.0002348
NM_004960	fused in sarcoma	0.0102467
NM_004978	potassium voltage-gated channel, Shaw-related subfamily, member 4	0.0026533
NM_005001	NADH dehydrogenase (ubiquinone) 1 alpha subcomplex, 7, 14.5kDa	0.0021908

NM_005004	NADH dehydrogenase (ubiquinone) 1 beta subcomplex, 8, 19kDa	0.0017759
NM_005007	nuclear factor of kappa light polypeptide gene enhancer in B-cells inhibitor-like 1	0.0269362
NM_005012	receptor tyrosine kinase-like orphan receptor 1	0.014399
NM_005015	oxidase (cytochrome c) assembly 1-like	0.0234427
NM_005017	phosphate cytidylyltransferase 1, choline, alpha	0.011251
NM_005024	serpin peptidase inhibitor, clade B (ovalbumin), member 10	0.0283541
NM_005032	plastin 3	0.0247094
NM_005034	polymerase (RNA) II (DNA directed) polypeptide K, 7.0kDa	0.00636
NM_005051	glutaminyl-tRNA synthetase	0.0346348
NM_005080	X-box binding protein 1	0.0029484
NM_005087	fragile X mental retardation, autosomal homolog 1	0.0035311
NM_005096	zinc finger, MYM-type 3	0.0374087
NM_005098	musculin	0.0032297
NM_005101	ISG15 ubiquitin-like modifier	0.0304988
NM_005117	fibroblast growth factor 19	0.0228438
NM_005118	tumor necrosis factor (ligand) superfamily, member 15	0.0254952
NM_005127	C-type lectin domain family 2, member B	0.0220171
NM_005131	THO complex 1	0.0416462
NM_005134	protein phosphatase 4, regulatory subunit 1	0.0098486
NM_005145	guanine nucleotide binding protein (G protein), gamma 7	0.0066652
NM_005157	c-abl oncogene 1, non-receptor tyrosine kinase	0.030812
NM_005163	v-akt murine thymoma viral oncogene homolog 1	0.0206581
NM_005165	aldolase C, fructose-bisphosphate	0.0192815
NM_005168	Rho family GTPase 3	0.0225409
NM_005174	ATP synthase, H transporting, mitochondrial F1 complex, gamma polypeptide 1	0.0069207
NM_005175	ATP synthase, H transporting, mitochondrial Fo complex, subunit C1 (subunit 9)	0.0376153
NM_005182	carbonic anhydrase VII	0.0315512
NM_005188	Cbl proto-oncogene, E3 ubiquitin protein ligase	0.0209806
NM_005192	cyclin-dependent kinase inhibitor 3	3.463E-05
NM_005205	cytochrome c oxidase subunit VIa polypeptide 2	0.017051
NM_005264	GDNF family receptor alpha 1	0.0423873
NM_005274	guanine nucleotide binding protein (G protein), gamma 5	0.0096006
NM_005300	G protein-coupled receptor 34	0.0024311

NM_005312	Rap guanine nucleotide exchange factor (GEF) 1	0.0056571
NM_005323	histone cluster 1, H1t	0.0240128
NM_005332	hemoglobin, zeta	0.0120866
NM_005337	NCK-associated protein 1-like	0.0189677
NM_005339	ubiquitin-conjugating enzyme E2K	0.0051894
NM_005368	myoglobin	0.0237526
NM_005374	membrane protein, palmitoylated 2 (MAGUK p55 subfamily member 2)	0.0146189
NM_005383	sialidase 2 (cytosolic sialidase)	0.0120003
NM_005385	natural killer-tumor recognition sequence	0.0224968
NM_005389	protein-L-isoaspartate (D-aspartate) O-methyltransferase	0.0018277
NM_005396	pancreatic lipase-related protein 2	0.0352979
NM_005397	podocalyxin-like	0.0172409
NM_005402	v-ral simian leukemia viral oncogene homolog A (ras related)	0.045323
NM_005412	serine hydroxymethyltransferase 2 (mitochondrial)	0.0221628
NM_005419	signal transducer and activator of transcription 2, 113kDa	0.0135905
NM_005428	vav 1 guanine nucleotide exchange factor	0.0438531
NM_005429	vascular endothelial growth factor C	0.0341399
NM_005430	wingless-type MMTV integration site family, member 1	0.0487684
NM_005445	structural maintenance of chromosomes 3	0.0072176
NM_005448	bone morphogenetic protein 15	0.0445933
NM_005475	SH2B adaptor protein 3	0.0279585
NM_005507	cofilin 1 (non-muscle)	0.0105741
NM_005511	melan-A	0.0109822
NM_005518	3-hydroxy-3-methylglutaryl-CoA synthase 2 (mitochondrial)	0.0157869
NM_005520	heterogeneous nuclear ribonucleoprotein H1 (H)	0.0076993
NM_005531	interferon, gamma-inducible protein 16	0.0047914
NM_005544	insulin receptor substrate 1	0.0034637
NM_005548	lysyl-tRNA synthetase	0.0404083
NM_005566	lactate dehydrogenase A	0.0025006
NM_005570	lectin, mannose-binding, 1	0.0020067
NM_005605	protein phosphatase 3, catalytic subunit, gamma isozyme	0.0150641
NM_005606	legumain	0.0135408
NM_005622	acyl-CoA synthetase medium-chain family member 3	0.0089435

NM_005623	chemokine (C-C motif) ligand 8	0.0160894
NM_005625	syndecan binding protein (syntenin)	0.0263496
NM_005627	serum/glucocorticoid regulated kinase 1	0.0150385
NM_005631	smoothened, frizzled family receptor	0.0001953
NM_005639	synaptotagmin I	0.0499662
NM_005647	transducin (beta)-like 1X-linked	0.033666
NM_005654	nuclear receptor subfamily 2, group F, member 1	0.0493759
NM_005668	ST8 alpha-N-acetyl-neuraminide alpha-2,8-sialyltransferase 4	0.0204769
NM_005679	TATA box binding protein (TBP)-associated factor, RNA polymerase I, C, 110kDa	0.0070927
NM_005701	snurportin 1	0.031601
NM_005703	HECT, UBA and WWE domain containing 1, E3 ubiquitin protein ligase	0.0244623
NM_005714	potassium channel, subfamily K, member 7	0.0139871
NM_005732	RAD50 homolog (S. cerevisiae)	0.0374731
NM_005738	ADP-ribosylation factor-like 4A	0.0402412
NM_005760	CCAAT/enhancer binding protein (C/EBP), zeta	0.0162922
NM_005766	FERM, RhoGEF (ARHGEF) and pleckstrin domain protein 1 (chondrocyte-derived)	0.0334238
NM_005772	RNA terminal phosphate cyclase-like 1	0.0437672
NM_005777	RNA binding motif protein 6	0.0400275
NM_005787	ALG3, alpha-1,3- mannosyltransferase	0.0109359
NM_005796	nuclear transport factor 2	0.0172831
NM_005805	proteasome (prosome, macropain) 26S subunit, non-ATPase, 14	0.0260783
NM_005813	protein kinase D3	0.0417076
NM_005824	leucine rich repeat containing 17	0.0394871
NM_005828	DDB1 and CUL4 associated factor 7	0.002918
NM_005845	ATP-binding cassette, sub-family C (CFTR/MRP), member 4	0.0003931
NM_005855	receptor (G protein-coupled) activity modifying protein 1	0.0408973
NM_005875	eukaryotic translation initiation factor 1B	0.0490407
NM_005884	p21 protein (Cdc42/Rac)-activated kinase 4	0.0010309
NM_005891	acetyl-CoA acetyltransferase 2	0.0039065
NM_005892	formin-like 1	0.001575
NM_005915	minichromosome maintenance complex component 6	0.007914
NM_005917	malate dehydrogenase 1, NAD (soluble)	0.0061693
NM_005922	mitogen-activated protein kinase kinase kinase 4	0.0150767

NM_005935	AF4/FMR2 family, member 1	0.0265015
NM_005998	chaperonin containing TCP1, subunit 3 (gamma)	0.0167586
NM_006002	ubiquitin carboxyl-terminal esterase L3 (ubiquitin thiolesterase)	0.0209721
NM_006003	ubiquinol-cytochrome c reductase, Rieske iron-sulfur polypeptide 1	0.031345
NM_006004	ubiquinol-cytochrome c reductase hinge protein	0.0070133
NM_006010	mesencephalic astrocyte-derived neurotrophic factor	0.0020141
NM_006013	ribosomal protein L10	0.0054835
NM_006018	hydroxycarboxylic acid receptor 3	0.0097806
NM_006022	TSC22 domain family, member 1	0.0182611
NM_006025	endonuclease, polyU-specific	0.0414102
NM_006042	heparan sulfate (glucosamine) 3-O-sulfotransferase 3A1	0.0037775
NM_006044	histone deacetylase 6	0.0078844
NM_006063	kelch-like family member 41	0.0146925
NM_006065	signal-regulatory protein beta 1	0.0270479
NM_006066	aldo-keto reductase family 1, member A1 (aldehyde reductase)	0.0097795
NM_006089	sex comb on midleg-like 2 (Drosophila)	0.0467525
NM_006117	enoyl-CoA delta isomerase 2	0.0292443
NM_006118	HCLS1 associated protein X-1	0.0001135
NM_006121	keratin 1	0.0045172
NM_006152	lymphoid-restricted membrane protein	0.0082608
NM_006160	neuronal differentiation 2	0.0491261
NM_006167	NK3 homeobox 1	0.0384802
NM_006176	neurogranin (protein kinase C substrate, RC3)	0.0067603
NM_006184	nucleobindin 1	0.0184962
NM_006186	nuclear receptor subfamily 4, group A, member 2	0.0427626
NM_006203	phosphodiesterase 4D, cAMP-specific	0.009558
NM_006204	phosphodiesterase 6C, cGMP-specific, cone, alpha prime	0.0341445
NM_006227	phospholipid transfer protein	0.0222337
NM_006233	polymerase (RNA) II (DNA directed) polypeptide I, 14.5kDa	0.0392817
NM_006236	POU class 3 homeobox 3	0.0085973
NM_006249	proline-rich protein BstNI subfamily 3	0.020174
NM_006259	protein kinase, cGMP-dependent, type II	0.0175321
NM_006269	retinitis pigmentosa 1 (autosomal dominant)	0.0370147

NM_006270	related RAS viral (r-ras) oncogene homolog	0.0139252
NM_006280	signal sequence receptor, delta	0.0488642
NM_006286	transcription factor Dp-2 (E2F dimerization partner 2)	0.0132087
NM_006287	tissue factor pathway inhibitor (lipoprotein-associated coagulation inhibitor)	0.0094375
NM_006294	ubiquinol-cytochrome c reductase binding protein	0.0054195
NM_006296	vaccinia related kinase 2	0.0172676
NM_006302	mannosyl-oligosaccharide glucosidase	0.030214
NM_006303	aminoacyl tRNA synthetase complex-interacting multifunctional protein 2	0.0484786
NM_006304	split hand/foot malformation (ectrodactyly) type 1	0.0029412
NM_006335	translocase of inner mitochondrial membrane 17 homolog A (yeast)	0.040854
NM_006347	peptidylprolyl isomerase H (cyclophilin H)	0.006878
NM_006348	component of oligomeric golgi complex 5	0.0025367
NM_006350	follistatin	0.00394
NM_006351	translocase of inner mitochondrial membrane 44 homolog (yeast)	0.0057966
NM_006352	zinc finger and BTB domain containing 18	0.0215916
NM_006373	vesicle amine transport protein 1 homolog (T. californica)	0.0139642
NM_006406	peroxiredoxin 4	0.0012381
NM_006407	ADP-ribosylation-like factor 6 interacting protein 5	0.0453361
NM_006417	interferon-induced protein 44	0.0159887
NM_006429	chaperonin containing TCP1, subunit 7 (eta)	0.0198007
NM_006432	Niemann-Pick disease, type C2	0.0029543
NM_006437	poly (ADP-ribose) polymerase family, member 4	0.018536
NM_006444	structural maintenance of chromosomes 2	0.0142577
NM_006467	polymerase (RNA) III (DNA directed) polypeptide G (32kD)	0.0228967
NM_006471	myosin, light chain 12A, regulatory, non-sarcomeric	0.0073507
NM_006472	thioredoxin interacting protein	0.0103715
NM_006476	ATP synthase, H transporting, mitochondrial Fo complex, subunit G	0.0153804
NM_006495	ecotropic viral integration site 2B	0.0000487
NM_006504	protein tyrosine phosphatase, receptor type, E	0.000871
NM_006514	sodium channel, voltage-gated, type X, alpha subunit	0.0112545
NM_006519	dynein, light chain, Tctex-type 1	0.0309058
NM_006526	zinc finger protein 217	0.035111
NM_006528	tissue factor pathway inhibitor 2	0.0269571

NM_006530	YEATS domain containing 4	0.0433771
NM_006540	nuclear receptor coactivator 2	0.0010302
NM_006554	metaxin 2	0.0006877
NM_006561	CUGBP, Elav-like family member 2	0.0065552
NM_006570	Ras-related GTP binding A	0.0001415
NM_006580	claudin 16	0.0467838
NM_006590	ubiquitin specific peptidase 39	0.0304188
NM_006602	transcription factor-like 5 (basic helix-loop-helix)	0.0274547
NM_006608	putative homeodomain transcription factor 1	0.0223102
NM_006620	HBS1-like (S. cerevisiae)	0.0004235
NM_006627	processing of precursor 4, ribonuclease P/MRP subunit (S. cerevisiae)	0.0064902
NM_006666	RuvB-like 2 (E. coli)	0.0034683
NM_006674	HLA complex P5 (non-protein coding)	0.0003176
NM_006682	fibrinogen-like 2	0.0147529
NM_006694	jumping translocation breakpoint	0.0217461
NM_006705	growth arrest and DNA-damage-inducible, gamma	0.0429496
NM_006706	transcription elongation regulator 1	0.0497861
NM_006708	glyoxalase I	0.0396742
NM_006712	Fas-activated serine/threonine kinase	0.0088299
NM_006729	diaphanous-related formin 2	0.0209935
NM_006738	A kinase (PRKA) anchor protein 13	0.0027284
NM_006745	methylsterol monooxygenase 1	0.00448
NM_006748	Src-like-adaptor	0.01582
NM_006750	syntrophin, beta 2 (dystrophin-associated protein A1, 59kDa, basic component 2)	0.0114926
NM_006756	transcription elongation factor A (SII), 1	0.0059692
NM_006762	lysosomal protein transmembrane 5	0.0075203
NM_006763	BTG family, member 2	0.0086339
NM_006783	gap junction protein, beta 6, 30kDa	0.0008072
NM_006786	urotensin 2	0.041484
NM_006793	peroxiredoxin 3	0.0162156
NM_006805	heterogeneous nuclear ribonucleoprotein A0	0.0348796
NM_006810	protein disulfide isomerase family A, member 5	0.0023462
NM_006811	serine incorporator 3	0.0138353

NM_006818	myeloid/lymphoid or mixed-lineage leukemia (trithorax homolog, Drosophila); translocated to, 11	0.0486622
NM_006820	interferon-induced protein 44-like	0.0130133
NM_006824	EBNA1 binding protein 2	0.0037273
NM_006825	cytoskeleton-associated protein 4	0.0086611
NM_006826	tyrosine 3-monooxygenase/tryptophan 5-monooxygenase activation protein, theta polypeptide	0.0046369
NM_006830	ubiquinol-cytochrome c reductase, complex III subunit XI	0.0089386
NM_006854	KDEL (Lys-Asp-Glu-Leu) endoplasmic reticulum protein retention receptor 2	0.0259523
NM_006855	KDEL (Lys-Asp-Glu-Leu) endoplasmic reticulum protein retention receptor 3	0.0014148
NM_006860	intraflagellar transport 27 homolog (Chlamydomonas)	0.0020251
NM_006875	pim-2 oncogene	0.0101749
NM_006886	ATP synthase, H transporting, mitochondrial F1 complex, epsilon subunit	0.0042086
NM_006892	DNA (cytosine-5-)-methyltransferase 3 beta	0.0155616
NM_006896	homeobox A7	0.0409525
NM_006898	homeobox D3	0.0432434
NM_006901	myosin IXA	0.0200786
NM_006928	premelanosome protein	0.0268901
NM_006931	solute carrier family 2 (facilitated glucose transporter), member 3	0.0268257
NM_006933	solute carrier family 5 (sodium/myo-inositol cotransporter), member 3	0.0356849
NM_006963	zinc finger protein 22	0.003587
NM_006980	mitochondrial transcription termination factor	0.0449333
NM_007011	abhydrolase domain containing 2	0.0121453
NM_007056	CLK4-associating serine/arginine rich protein	0.0360549
NM_007057	ZW10 interacting kinetochore protein	0.0259995
NM_007064	kalirin, RhoGEF kinase	0.0101026
NM_007065	cell division cycle 37	0.0062257
NM_007066	protein kinase (cAMP-dependent, catalytic) inhibitor gamma	0.0195832
NM_007075	WD repeat domain 45	0.001488
NM_007080	LSM6 homolog, U6 small nuclear RNA associated (S. cerevisiae)	0.043244
NM_007097	clathrin, light chain B	0.0066534
NM_007100	ATP synthase, H transporting, mitochondrial Fo complex, subunit E	0.0057376
NM_007107	signal sequence receptor, gamma (translocon-associated protein gamma)	0.0174654
NM_007115	tumor necrosis factor, alpha-induced protein 6	0.0243918
NM_007122	upstream transcription factor 1	0.0432679

NM_007152	zinc finger protein 195	0.0031879
NM_007158	cold shock domain containing E1, RNA-binding	0.0054172
NM_007159	sarcolemma associated protein	0.0449308
NM_007208	mitochondrial ribosomal protein L3	0.0014286
NM_007211	Ras association (RalGDS/AF-6) domain family (N-terminal) member 8	0.0197418
NM_007217	programmed cell death 10	0.0277601
NM_007218	ring finger protein 139	0.0439129
NM_007235	exportin, tRNA	0.0015075
NM_007289	membrane metallo-endopeptidase	3.01E-06
NM_007317	kinesin family member 22	0.0288384
NM_007318	presenilin 1	0.010802
NM_007326	cytochrome b5 reductase 3	0.0118949
NM_007350	pleckstrin homology-like domain, family A, member 1	0.0011114
NM_007360	killer cell lectin-like receptor subfamily K, member 1	0.0006561
NM_009587	lectin, galactoside-binding, soluble, 9	0.0209722
NM_012069	ATPase, Naسخ transporting, beta 4 polypeptide	0.0044941
NM_012071	COMM domain containing 3	0.0304495
NM_012081	elongation factor, RNA polymerase II, 2	0.0283491
NM_012090	microtubule-actin crosslinking factor 1	0.0053477
NM_012096	adaptor protein, phosphotyrosine interaction, PH domain and leucine zipper containing 1	0.0086315
NM_012101	tripartite motif containing 29	0.0448358
NM_012111	AHA1, activator of heat shock 90kDa protein ATPase homolog 1 (yeast)	0.0183882
NM_012113	carbonic anhydrase XIV	0.0307819
NM_012115	caspase 8 associated protein 2	0.0396125
NM_012117	chromobox homolog 5	0.0002087
NM_012131	claudin 17	0.0494073
NM_012155	echinoderm microtubule associated protein like 2	0.0301911
NM_012164	F-box and WD repeat domain containing 2	0.0285244
NM_012177	F-box protein 5	0.0343799
NM_012179	F-box protein 7	0.000878
NM_012188	forkhead box I1	0.002466
NM_012222	mutY homolog (E. coli)	0.0203342
NM_012225	nucleotide binding protein 2	0.039983

NM_012229	5'-nucleotidase, cytosolic II	0.0031303
NM_012247	selenophosphate synthetase 1	0.0126045
NM_012255	5'-3' exoribonuclease 2	0.0362925
NM_012259	hairy/enhancer-of-split related with YRPW motif 2	0.0276972
NM_012260	2-hydroxyacyl-CoA lyase 1	0.0129629
NM_012325	microtubule-associated protein, RP/EB family, member 1	0.0082566
NM_012337	coiled-coil domain containing 19	0.0160076
NM_012341	GTP binding protein 4	0.0016124
NM_012342	BMP and activin membrane-bound inhibitor homolog (Xenopus laevis)	0.0141006
NM_012351	olfactory receptor, family 10, subfamily J, member 1	0.0135754
NM_012404	acidic (leucine-rich) nuclear phosphoprotein 32 family, member D	0.0117698
NM_012413	glutaminyl-peptide cyclotransferase	3.877E-05
NM_012420	interferon-induced protein with tetratricopeptide repeats 5	0.0038604
NM_012455	pleckstrin and Sec7 domain containing 4	0.0209775
NM_012456	translocase of inner mitochondrial membrane 10 homolog (yeast)	0.033608
NM_012460	translocase of inner mitochondrial membrane 9 homolog (yeast)	0.0114986
NM_012474	uridine-cytidine kinase 2	0.0346928
NM_012479	tyrosine 3-monooxygenase/tryptophan 5-monooxygenase activation protein, gamma polypeptide	0.0011296
NM_012483	granulysin	0.0249852
NM_013229	apoptotic peptidase activating factor 1	0.007057
NM_013235	drosha, ribonuclease type III	0.002123
NM_013236	ataxin 10	0.0482647
NM_013243	secretogranin III	0.0439717
NM_013270	protease, serine, 50	0.0108242
NM_013291	cleavage and polyadenylation specific factor 1, 160kDa	0.0195496
NM_013300	family with sequence similarity 216, member A	0.0261319
NM_013328	pyrroline-5-carboxylate reductase family, member 2	0.0026434
NM_013345	G protein-coupled receptor 132	0.0084604
NM_013347	replication protein A4, 30kDa	0.0304552
NM_013385	cytohesin 4	0.0013336
NM_013387	ubiquinol-cytochrome c reductase, complex III subunit X	0.0033118
NM_013388	prolactin regulatory element binding	0.0288853
NM_013393	FtsJ RNA methyltransferase homolog 2 (E. coli)	0.0016063

NM_013396	ubiquitin specific peptidase 25	0.0426142
NM_013437	low density lipoprotein receptor-related protein 12	0.0436695
NM_013439	paired immunoglobin-like type 2 receptor alpha	0.0037815
NM_013442	stomatin (EPB72)-like 2	0.0297695
NM_013446	makorin ring finger protein 1	0.0292542
NM_013450	bromodomain adjacent to zinc finger domain, 2B	0.0006819
NM_013982	neuregulin 2	0.0272977
NM_013989	deiodinase, iodothyronine, type II	0.0113132
NM_014002	inhibitor of kappa light polypeptide gene enhancer in B-cells, kinase epsilon	0.0255442
NM_014015	Dexi homolog (mouse)	0.0183066
NM_014018	mitochondrial ribosomal protein S28	0.0011844
NM_014037	solute carrier family 6, member 16	0.0060966
NM_014056	HIG1 hypoxia inducible domain family, member 1A	0.0300519
NM_014068	psoriasis susceptibility 1 candidate 1	0.0330521
NM_014069	psoriasis susceptibility 1 candidate 2	0.0249799
NM_014112	trichorhinophalangeal syndrome I	0.0355688
NM_014138	family with sequence similarity 156, member A	0.0308402
NM_014142	nudix (nucleoside diphosphate linked moiety X)-type motif 5	0.0488436
NM_014145	transmembrane protein 230	0.0170225
NM_014165	NADH dehydrogenase (ubiquinone) complex I, assembly factor 4	0.0403459
NM_014168	methyltransferase like 5	0.0456977
NM_014169	charged multivesicular body protein 4A	0.0260874
NM_014175	mitochondrial ribosomal protein L15	0.0024469
NM_014182	ORM1-like 2 (S. cerevisiae)	0.0282888
NM_014205	zinc finger, HIT-type containing 2	0.0288027
NM_014210	ecotropic viral integration site 2A	0.028458
NM_014222	NADH dehydrogenase (ubiquinone) 1 alpha subcomplex, 8, 19kDa	0.0003425
NM_014230	signal recognition particle 68kDa	0.0312291
NM_014232	vesicle-associated membrane protein 2 (synaptobrevin 2)	0.0109588
NM_014239	eukaryotic translation initiation factor 2B, subunit 2 beta, 39kDa	0.0165814
NM_014247	Rap guanine nucleotide exchange factor (GEF) 2	0.0340192
NM_014253	teneurin transmembrane protein 1	0.032115
NM_014264	polo-like kinase 4	0.0477121

NM_014267	chromosome 11 open reading frame 58	0.0223452
NM_014275	mannosyl (alpha-1,3-)-glycoprotein beta-1,4-N-acetylglucosaminyltransferase, isozyme B	0.0196451
NM_014288	integrin beta 3 binding protein (beta3-endonexin)	0.0154092
NM_014300	SEC11 homolog A (S. cerevisiae)	0.0055117
NM_014302	Sec61 gamma subunit	0.0054297
NM_014314	DEAD (Asp-Glu-Ala-Asp) box polypeptide 58	0.0235641
NM_014317	prenyl (decaprenyl) diphosphate synthase, subunit 1	0.0450967
NM_014330	protein phosphatase 1, regulatory subunit 15A	0.0023754
NM_014333	cell adhesion molecule 1	0.0010221
NM_014335	EP300 interacting inhibitor of differentiation 1	0.0001784
NM_014350	tumor necrosis factor, alpha-induced protein 8	0.0413792
NM_014363	spastic ataxia of Charlevoix-Saguenay (sacsin)	0.0029979
NM_014364	glyceraldehyde-3-phosphate dehydrogenase, spermatogenic	0.0065749
NM_014383	zinc finger and BTB domain containing 32	0.0214254
NM_014402	ubiquinol-cytochrome c reductase, complex III subunit VII, 9.5kDa	0.0342352
NM_014406	chaperonin containing TCP1, subunit 8 (theta)-like 2	0.0156253
NM_014439	interleukin 37	0.0373167
NM_014442	sialic acid binding Ig-like lectin 8	0.0242291
NM_014464	tubulointerstitial nephritis antigen	0.0017653
NM_014483	RNA binding motif, single stranded interacting protein 3	0.0020823
NM_014500	HIV-1 Tat specific factor 1	0.025322
NM_014505	potassium large conductance calcium-activated channel, subfamily M, beta member 4	0.0379636
NM_014509	serine hydrolase-like 2	0.0200437
NM_014519	zinc finger protein 232	0.0110174
NM_014548	tropomodulin 2 (neuronal)	0.0139741
NM_014576	APOBEC1 complementation factor	0.0421339
NM_014606	HECT and RLD domain containing E3 ubiquitin protein ligase 3	0.0391901
NM_014617	crystallin, gamma A	0.0027916
NM_014618	deleted in bladder cancer 1	0.0015077
NM_014622	von Willebrand factor A domain containing 5A	0.0076119
NM_014623	male-enhanced antigen 1	0.004145
NM_014639	tetratricopeptide repeat domain 37	0.0023689
NM_014666	clathrin interactor 1	0.0085929

NM_014673	ER membrane protein complex subunit 2	0.0195552
NM_014685	homocysteine-inducible, endoplasmic reticulum stress-inducible, ubiquitin-like domain member 1	0.0481266
NM_014705	dedicator of cytokinesis 4	6.855E-05
NM_014726	TBK1 binding protein 1	0.0090189
NM_014727	lysine (K)-specific methyltransferase 2B	0.0181596
NM_014736	KIAA0101	0.0351219
NM_014747	regulating synaptic membrane exocytosis 3	0.0176619
NM_014763	mitochondrial ribosomal protein L19	0.0020614
NM_014764	DAZ associated protein 2	0.0257986
NM_014767	sparc/osteonectin, cwcv and kazal-like domains proteoglycan (testican) 2	0.037951
NM_014773	KIAA0141	0.0245139
NM_014774	EF-hand calcium binding domain 14	0.012641
NM_014799	hephaestin	0.0172939
NM_014800	engulfment and cell motility 1	0.001267
NM_014801	pecanex-like 2 (Drosophila)	0.0270713
NM_014814	proteasome (prosome, macropain) 26S subunit, non-ATPase, 6	0.004461
NM_014830	zinc finger and BTB domain containing 39	0.0438939
NM_014857	RAB GTPase activating protein 1-like	0.0135724
NM_014863	carbohydrate (N-acetylgalactosamine 4-sulfate 6-O) sulfotransferase 15	0.0147746
NM_014867	kelch repeat and BTB (POZ) domain containing 11	0.0236512
NM_014874	mitofusin 2	0.0013893
NM_014875	kinesin family member 14	0.043828
NM_014882	Rho GTPase activating protein 25	0.022901
NM_014887	NEDD4 binding protein 2-like 2	0.0224212
NM_014899	Rho-related BTB domain containing 3	0.0229454
NM_014907	FERM and PDZ domain containing 1	0.0026373
NM_014918	chondroitin sulfate synthase 1	0.0248807
NM_014922	NLR family, pyrin domain containing 1	0.0006538
NM_014925	R3H domain containing 2	0.0016602
NM_014930	zinc finger protein 510	0.0215265
NM_014933	SEC31 homolog A (S. cerevisiae)	0.0000231
NM_014945	actin binding LIM protein family, member 3	0.000869
NM_014967	FANCD2/FANCI-associated nuclease 1	0.0425049

NM_014969	WD repeat domain 47	0.0056637
NM_014999	RAB21, member RAS oncogene family	0.0457178
NM_015032	PDS5, regulator of cohesion maintenance, homolog B (S. cerevisiae)	0.0034245
NM_015216	diphosphoinositol pentakisphosphate kinase 2	0.0223906
NM_015376	RAS guanyl releasing protein 3 (calcium and DAG-regulated)	0.0467924
NM_015393	prostate androgen-regulated mucin-like protein 1	0.0385224
NM_015400	SMAD family member 3	0.0205003
NM_015415	mitochondrial pyruvate carrier 2	0.0295591
NM_015455	CCR4-NOT transcription complex, subunit 6	0.0050777
NM_015458	myotubularin related protein 9	0.0041317
NM_015492	chromosome 15 open reading frame 39	0.032939
NM_015509	NECAP endocytosis associated 1	0.0348239
NM_015523	RNA exonuclease 2	0.0079632
NM_015556	signal-induced proliferation-associated 1 like 1	0.0086733
NM_015599	phosphoglucomutase 3	0.0461209
NM_015640	SERPINE1 mRNA binding protein 1	0.0008719
NM_015679	TruB pseudouridine (psi) synthase homolog 2 (E. coli)	0.0300785
NM_015698	G patch domain and KOW motifs	0.0314901
NM_015700	NFU1 iron-sulfur cluster scaffold homolog (S. cerevisiae)	0.0242427
NM_015714	G0/G1switch 2	0.0321507
NM_015836	tryptophanyl tRNA synthetase 2, mitochondrial	0.0456256
NM_015848	keratin 76	0.0327203
NM_015895	geminin, DNA replication inhibitor	0.0222671
NM_015911	zinc finger protein 691	0.0045796
NM_015920	ribosomal protein S27-like	0.0003859
NM_015921	cutA divalent cation tolerance homolog (E. coli)	0.0091517
NM_015922	NAD(P) dependent steroid dehydrogenase-like	0.0171311
NM_015939	tRNA methyltransferase 6 homolog (S. cerevisiae)	0.0033534
NM_015954	deoxyribose-phosphate aldolase (putative)	0.0368744
NM_015960	cutC copper transporter homolog (E. coli)	0.0476234
NM_015971	mitochondrial ribosomal protein S7	0.0227704
NM_015972	polymerase (RNA) I polypeptide D, 16kDa	0.0203584
NM_015973	galanin/GMAP prepropeptide	0.0392197

NM_015978	TNNI3 interacting kinase	0.0410907
NM_015984	ubiquitin carboxyl-terminal hydrolase L5	0.0473117
NM_015986	cytokine receptor-like factor 3	0.0376069
NM_015991	complement component 1, q subcomponent, A chain	0.0030507
NM_016001	UTP18 small subunit (SSU) processome component homolog (yeast)	0.0074605
NM_016008	dynein, cytoplasmic 2, light intermediate chain 1	0.0067382
NM_016010	zinc finger, C2HC-type containing 1A	0.0011336
NM_016019	LUC7-like 2 (S. cerevisiae)	0.0304104
NM_016034	mitochondrial ribosomal protein S2	0.0199902
NM_016052	ribosomal RNA processing 15 homolog (S. cerevisiae)	0.0214754
NM_016053	coiled-coil domain containing 53	0.0062619
NM_016058	TP53RK binding protein	0.0076216
NM_016061	yippee-like 5 (Drosophila)	0.0054378
NM_016063	HD domain containing 2	0.0058302
NM_016065	mitochondrial ribosomal protein S16	0.0062573
NM_016067	mitochondrial ribosomal protein S18C	0.0214053
NM_016068	fission 1 (mitochondrial outer membrane) homolog (S. cerevisiae)	0.0361616
NM_016072	golgi transport 1B	0.0262438
NM_016085	all-trans retinoic acid-induced differentiation factor	0.0198866
NM_016095	GINS complex subunit 2 (Psf2 homolog)	0.0042612
NM_016096	zinc finger protein 706	0.0055922
NM_016097	immediate early response 3 interacting protein 1	0.0199609
NM_016100	N(alpha)-acetyltransferase 20, NatB catalytic subunit	0.0009459
NM_016107	zinc finger RNA binding protein	0.0314942
NM_016126	heat shock protein family B (small), member 11	0.0417969
NM_016130	DEAD (Asp-Glu-Ala-Asp) box polypeptide 46	0.0238312
NM_016138	coenzyme Q7 homolog, ubiquinone (yeast)	0.0324178
NM_016179	transient receptor potential cation channel, subfamily C, member 4	0.0157266
NM_016200	N(alpha)-acetyltransferase 38, NatC auxiliary subunit	0.0051864
NM_016203	protein kinase, AMP-activated, gamma 2 non-catalytic subunit	0.0383206
NM_016205	platelet derived growth factor C	0.0109906
NM_016207	cleavage and polyadenylation specific factor 3, 73kDa	0.0001563
NM_016209	trafficking protein particle complex 2-like	0.0407755

NM_016255	family with sequence similarity 8, member A1	0.0224743
NM_016270	Kruppel-like factor 2 (lung)	0.0036943
NM_016272	transducer of ERBB2, 2	0.0435803
NM_016274	pleckstrin homology domain containing, family O member 1	0.0089434
NM_016277	RAB23, member RAS oncogene family	0.0499513
NM_016279	cadherin 9, type 2 (T1-cadherin)	0.0243486
NM_016289	calcium binding protein 39	0.0017474
NM_016304	ribosomal L24 domain containing 1	0.0009095
NM_016310	polymerase (RNA) III (DNA directed) polypeptide K, 12.3 kDa	0.0454703
NM_016332	methionine sulfoxide reductase B1	0.0310804
NM_016391	NOP16 nucleolar protein	0.0071378
NM_016403	CWC15 spliceosome-associated protein homolog (S. cerevisiae)	0.002204
NM_016404	tRNA methyltransferase 11-2 homolog (S. cerevisiae)	0.0145222
NM_016411	chromosome 11 open reading frame 73	0.0086509
NM_016417	glutaredoxin 5	0.0038703
NM_016428	ABI family, member 3	0.004213
NM_016448	denticleless E3 ubiquitin protein ligase homolog (Drosophila)	0.0259561
NM_016456	transmembrane protein 9	0.0475976
NM_016457	protein kinase D2	0.0236486
NM_016468	COX16 cytochrome c oxidase assembly homolog (S. cerevisiae)	0.0019188
NM_016476	anaphase promoting complex subunit 11	0.0317905
NM_016494	ring finger protein 181	0.0038348
NM_016500	polysaccharide biosynthesis domain containing 1	0.0030532
NM_016504	mitochondrial ribosomal protein L27	0.0070603
NM_016505	zinc finger, CCHC domain containing 17	0.0006374
NM_016510	selenocysteine lyase	0.000812
NM_016523	killer cell lectin-like receptor subfamily F, member 1	0.0436656
NM_016553	nucleoporin 62kDa	0.0210508
NM_016558	SCAN domain containing 1	0.0366281
NM_016563	RAS-like, family 12	0.036171
NM_016568	relaxin/insulin-like family peptide receptor 3	0.0466933
NM_016580	protocadherin 12	0.0412955
NM_016583	BPI fold containing family A, member 1	0.0325483

NM_016589	translocase of inner mitochondrial membrane domain containing 1	0.0439587
NM_016614	tyrosyl-DNA phosphodiesterase 2	0.0126619
NM_016620	zinc finger protein 644	0.0330478
NM_016625	arginine/serine-rich coiled-coil 1	0.0270974
NM_016638	ADP-ribosylation-like factor 6 interacting protein 4	0.0137886
NM_016819	8-oxoguanine DNA glycosylase	0.010309
NM_016930	syntaxin 18	0.0255299
NM_016932	SIX homeobox 2	0.0169154
NM_016946	F11 receptor	0.0346675
NM_017410	homeobox C13	0.005178
NM_017421	coenzyme Q3 methyltransferase	0.0405362
NM_017446	mitochondrial ribosomal protein L39	0.0199
NM_017458	major vault protein	0.0233298
NM_017523	XIAP associated factor 1	0.0222003
NM_017528	Williams Beuren syndrome chromosome region 22	0.0263566
NM_017548	CDV3 homolog (mouse)	0.018317
NM_017575	smg-6 homolog, nonsense mediated mRNA decay factor (C. elegans)	0.0241164
NM_017577	GRAM domain containing 1C	0.0021
NM_017606	zinc finger protein 395	0.0277446
NM_017631	DEAD (Asp-Glu-Ala-Asp) box polypeptide 60	0.0004413
NM_017634	potassium channel tetramerisation domain containing 9	0.0487601
NM_017647	FtsJ homolog 3 (E. coli)	0.0030319
NM_017661	zinc finger protein 280D	0.024131
NM_017679	breast carcinoma amplified sequence 3	0.0024144
NM_017695	synaptotagmin-like 2	0.0388927
NM_017698	transmembrane protein 164	0.0250822
NM_017707	ArfGAP with SH3 domain, ankyrin repeat and PH domain 3	0.0182318
NM_017712	pyroglutamyl-peptidase I	0.0001289
NM_017719	SNF related kinase	0.0065341
NM_017727	transmembrane protein 214	0.0080682
NM_017737	formin binding protein 1-like	0.0357601
NM_017742	zinc finger, CCHC domain containing 2	0.0353851
NM_017745	BCL6 corepressor	0.0492864

NM_017750	retinol saturase (all-trans-retinol 13,14-reductase)	0.0473509
NM_017777	Meckel syndrome, type 1	0.0419142
NM_017805	Ras interacting protein 1	0.0362674
NM_017825	ADP-ribosylhydrolase like 2	0.0259951
NM_017832	family with sequence similarity 206, member A	0.0071142
NM_017836	solute carrier family 41, member 3	0.0111118
NM_017840	mitochondrial ribosomal protein L16	0.046492
NM_017850	chromosome 1 open reading frame 109	0.0307697
NM_017851	poly (ADP-ribose) polymerase family, member 16	0.020923
NM_017855	odontogenic, ameloblast asssociated	0.0202114
NM_017864	integrator complex subunit 8	0.0211871
NM_017865	zinc finger protein 692	0.0197729
NM_017869	BTG3 associated nuclear protein	0.0402674
NM_017885	host cell factor C1 regulator 1 (XPO1 dependent)	0.0097665
NM_017892	PRP40 pre-mRNA processing factor 40 homolog A (S. cerevisiae)	0.0071921
NM_017906	PAK1 interacting protein 1	0.0078545
NM_017915	PARP1 binding protein	0.0170203
NM_017916	PIH1 domain containing 1	0.0268865
NM_017941	chromosome 17 open reading frame 80	0.0103994
NM_017942	BTB (POZ) domain containing 1	0.0497662
NM_017949	CUE domain containing 1	0.0316396
NM_017950	coiled-coil domain containing 40	0.0090986
NM_017956	tRNA methyltransferase 12 homolog (S. cerevisiae)	0.0373827
NM_017974	autophagy related 16-like 1 (S. cerevisiae)	0.0215832
NM_017993	ecto-NOX disulfide-thiol exchanger 1	0.0420325
NM_018011	arginine and glutamate rich 1	0.0406307
NM_018034	WD repeat domain 70	0.0054651
NM_018047	RNA binding motif protein 22	0.0273103
NM_018060	isoleucyl-tRNA synthetase 2, mitochondrial	0.0001796
NM_018062	Fanconi anemia, complementation group L	0.0214385
NM_018066	GPN-loop GTPase 2	0.0263347
NM_018084	coiled-coil domain containing 88A	0.0409535
NM_018086	fidgetin	0.0358829

NM_018100	EF-hand domain (C-terminal) containing 1	0.0195328
NM_018113	limb region 1 homolog (mouse)-like	0.0421483
NM_018123	asp (abnormal spindle) homolog, microcephaly associated (Drosophila)	0.0026829
NM_018127	elaC ribonuclease Z 2	0.0245509
NM_018135	mitochondrial ribosomal protein S18A	0.002739
NM_018141	mitochondrial ribosomal protein S10	0.0120364
NM_018155	solute carrier family 25 (pyrimidine nucleotide carrier), member 36	0.0115908
NM_018169	KIAA1551	0.0034689
NM_018172	family with sequence similarity 86, member C1	0.0123459
NM_018180	DEAH (Asp-Glu-Ala-His) box polypeptide 32	0.0048105
NM_018186	chromosome 1 open reading frame 112	0.0213759
NM_018190	Bardet-Biedl syndrome 7	0.0113672
NM_018211	ribonucleoprotein, PTB-binding 2	0.0005182
NM_018227	ubiquitin-like modifier activating enzyme 6	0.0337422
NM_018230	nucleoporin 133kDa	0.0013475
NM_018234	STEAP family member 3, metalloreductase	0.0062088
NM_018249	CDK5 regulatory subunit associated protein 2	0.0001083
NM_018256	WD repeat domain 12	0.0124915
NM_018275	chromosome 7 open reading frame 43	0.0325894
NM_018282	paraspeckle component 1	0.0450608
NM_018283	nudix (nucleoside diphosphate linked moiety X)-type motif 15	0.0277094
NM_018295	transmembrane protein 140	0.0145691
NM_018318	coiled-coil domain containing 91	0.0333949
NM_018321	BRX1, biogenesis of ribosomes, homolog (S. cerevisiae)	0.0307628
NM_018322	SAYSVFN motif domain containing 1	0.0194535
NM_018326	GTPase, IMAP family member 4	0.0219063
NM_018340	calcineurin-like phosphoesterase domain containing 1	0.0018996
NM_018359	UFM1-specific peptidase 2	0.0491075
NM_018366	biogenesis of lysosomal organelles complex-1, subunit 4, cappuccino	0.0158945
NM_018367	alkaline ceramidase 3	0.0213895
NM_018379	family with sequence similarity 63, member A	0.0110643
NM_018383	WD repeat domain 33	0.0091229
NM_018398	calcium channel, voltage-dependent, alpha 2/delta subunit 3	0.0394279

NM_018413	carbohydrate (chondroitin 4) sulfotransferase 11	0.0003429
NM_018416	forkhead box J2	0.0456737
NM_018434	ring finger protein 130	0.0046302
NM_018436	allantoicase	0.0300908
NM_018446	glycosyltransferase 8 domain containing 1	0.03739
NM_018449	ubiquitin associated protein 2	0.0043456
NM_018460	Rho GTPase activating protein 15	0.0055974
NM_018518	minichromosome maintenance complex component 10	0.0164317
NM_018534	neuropilin 2	0.0358075
NM_018556	signal-regulatory protein gamma	0.007139
NM_018621	mannosidase, alpha, class 2A, member 2	0.0246072
NM_018630	derlin 1	0.0313677
NM_018643	triggering receptor expressed on myeloid cells 1	7.416E-05
NM_018649	H2A histone family, member Y2	0.0010522
NM_018676	thrombospondin, type I, domain containing 1	0.0121674
NM_018685	anillin, actin binding protein	0.0030001
NM_018686	cytidine monophosphate N-acetylneuraminic acid synthetase	0.0053819
NM_018688	bridging integrator 3	0.0060365
NM_018944	MIS18 kinetochore protein homolog A (S. pombe)	0.0294142
NM_018950	major histocompatibility complex, class I, F	0.0084606
NM_018977	neuroligin 3	0.030807
NM_018980	taste receptor, type 2, member 5	0.0332549
NM_019005	missing oocyte, meiosis regulator, homolog (Drosophila)	0.0156068
NM_019008	Smith-Magenis syndrome chromosome region, candidate 7-like	0.0375462
NM_019037	exosome component 4	0.0008882
NM_019059	translocase of outer mitochondrial membrane 7 homolog (yeast)	0.0193659
NM_019063	echinoderm microtubule associated protein like 4	0.0021566
NM_019074	delta-like 4 (Drosophila)	0.0110915
NM_019089	hairy and enhancer of split 2 (Drosophila)	0.0277455
NM_019095	cardiolipin synthase 1	0.003205
NM_019101	apolipoprotein M	0.0392385
NM_019618	interleukin 36, gamma	0.0219382
NM_019619	par-3 partitioning defective 3 homolog (C. elegans)	0.0246711

NM_019848	solute carrier family 10 (sodium/bile acid cotransporter family), member 3	0.0061455
NM_020119	zinc finger CCCH-type, antiviral 1	0.0227871
NM_020133	1-acylglycerol-3-phosphate O-acyltransferase 4	0.047403
NM_020142	NADH dehydrogenase (ubiquinone) 1 alpha subcomplex, 4-like 2	0.003625
NM_020143	partner of NOB1 homolog (S. cerevisiae)	0.0027837
NM_020158	exosome component 5	0.021024
NM_020198	coiled-coil domain containing 47	0.0273025
NM_020199	chromosome 5 open reading frame 15	0.0140369
NM_020228	PR domain containing 10	0.0188954
NM_020232	proteasome (prosome, macropain) assembly chaperone 2	0.0221886
NM_020240	CDC42 small effector 2	0.0285418
NM_020242	kinesin family member 15	0.0339252
NM_020243	translocase of outer mitochondrial membrane 22 homolog (yeast)	0.0410973
NM_020348	cyclin M1	0.0096925
NM_020354	ectonucleoside triphosphate diphosphohydrolase 7	0.0125721
NM_020356	Cas scaffolding protein family member 4	0.005745
NM_020366	retinitis pigmentosa GTPase regulator interacting protein 1	0.0464986
NM_020367	poly (ADP-ribose) polymerase family, member 11	0.00827
NM_020370	G protein-coupled receptor 84	0.0006914
NM_020403	protocadherin 9	0.0030087
NM_020409	mitochondrial ribosomal protein L47	0.0304079
NM_020412	charged multivesicular body protein 1B	0.033112
NM_020415	resistin	0.0013115
NM_020418	poly(rC) binding protein 4	0.032886
NM_020448	NIPA-like domain containing 3	0.0150057
NM_020474	UDP-N-acetyl-alpha-D-galactosamine:polypeptide N-acetylgalactosaminyltransferase 1 (GalNAc-T1)	0.0027388
NM_020524	pre-B-cell leukemia homeobox interacting protein 1	0.0398512
NM_020530	oncostatin M	0.0190343
S72604	myeloid/lymphoid or mixed-lineage leukemia (trithorax homolog, Drosophila); translocated to, 6	0.0204139
S80797	insulin-like growth factor 2 receptor	0.0049099
S81522	eukaryotic translation elongation factor 1 beta 2	0.0125151
S82470	membrane bound O-acyltransferase domain containing 7	0.003096
S82769	gamma-aminobutyric acid (GABA) A receptor, gamma 3	0.0463586

U08853	nuclear factor (erythroid-derived 2)-like 1	0.0151932
U18937	histidyl-tRNA synthetase 2, mitochondrial	0.0244755
U20648	zinc finger protein 154	0.0201657
U23028	eukaryotic translation initiation factor 2B, subunit 5 epsilon, 82kDa	0.0455924
U27655	regulator of G-protein signaling 3	0.0357774
U28480	uncoupling protein 1 (mitochondrial, proton carrier)	0.0271592
U37689	polymerase (RNA) II (DNA directed) polypeptide H	0.0407545
U50383	SMYD family member 5	0.0169462
U50534	furry homolog (Drosophila)	0.0002936
U63828	chromosome 20 open reading frame 181	0.0419015
U66048	long intergenic non-protein coding RNA 894	0.0388164
U69645	zinc finger protein 32	0.0031977
U77493	notch 2	0.0326092
U77700	GCN1 general control of amino-acid synthesis 1-like 1 (yeast)	0.0237161
U79282	UBX domain protein 2B	0.0174886
U80756	lysine (K)-specific methyltransferase 2D	0.0220262
U82987	BCL2 binding component 3	0.0094074
U83115	absent in melanoma 1	0.0273773
U90653	zinc finger, DHHC-type containing 1	0.0490632
U90878	PDZ and LIM domain 1	0.0414796
U92544	melanoma antigen family D, 2	0.03912
U94386	RNA binding motif protein, Y-linked, family 3, member A pseudogene	0.0137317
U96173	one cut homeobox 1	0.0414119
X03324	apolipoprotein B	0.0263264
X05826	serpin peptidase inhibitor, clade A (alpha-1 antiproteinase, antitrypsin), member 1	0.0007164
X15183	heat shock protein 90kDa alpha (cytosolic), class A member 1	0.0115989
X15262	ret proto-oncogene	0.0051579
X52350	zinc finger protein 25	0.0121256
X52354	zinc finger protein 551	0.0069697
X59417	proteasome (prosome, macropain) subunit, alpha type, 6	0.0010592
X62534	high mobility group box 2	0.040269
X63417	DENN/MADD domain containing 4A	0.0470918
X64986	olfactory receptor, family 10, subfamily A, member 3	0.0376258

X73502	keratin 20	0.0030497
X74070	basic transcription factor 3	0.0326769
X75684	transmembrane 4 L six family member 1	0.0020363
X78712	glycerol kinase 2	0.0058535
X83412	B1 for mucin	0.0118641
X85106	ribosomal protein S6 kinase, 90kDa, polypeptide 2	0.0285628
X87825	olfactory receptor, family 7, subfamily E, member 47 pseudogene	0.0124083
X91648	purine-rich element binding protein A	0.0159755
X96646	small nucleolar RNA, C/D box 46	0.0017125
X96660	small nucleolar RNA, C/D box 60	0.041454
X97301	serine/arginine repetitive matrix 2	0.0083196
X97303	zinc finger, matrin-type 2	0.0233848
X98411	myosin IF	0.0324681
X98494	M-phase phosphoprotein 10 (U3 small nucleolar ribonucleoprotein)	0.0303074
Y08266	AT rich interactive domain 1B (SWI1-like)	0.0066335
Y10483	protein kinase, X-linked, pseudogene 1	0.0049757
Y10529	olfactory receptor, family 7, subfamily E, member 24	0.0392763
Y11158	small nucleolar RNA, H/ACA box 64	0.0351007
Y11161	small nucleolar RNA, H/ACA box 67	0.0037843
Y11162	small nucleolar RNA, H/ACA box 68	0.0040352
Y11710	collagen, type XIV, alpha 1	0.0382463
Y12839	meiosis 1 associated protein	0.0287432
Y17867	tenascin XB	0.0136669
Y19237	synaptotagmin VII	0.029652
Z11849	growth hormone receptor	0.0030093
Z25424	MAP kinase interacting serine/threonine kinase 2	0.001284
Z83801	dynein, axonemal, heavy chain 7	0.0293145
