# Supplementary material for: A Systems Biological Approach Reveals Multiple Crosstalk Mechanism between Gram-Positive and Negative Bacterial Infections: An Insight into Core Mechanism and Unique Molecular Signatures
Source: PLoS One. 2014 Feb 28;9(2):e89993. doi: 10.1371/journal.pone.0089993 (PMC3938579; doi:10.1371/journal.pone.0089993)
Supplement: Table S2 — Common pathways between TLR 2 and TLR 4. Identified common pathways (TLR2U4) mediated by both the TLRs suggests the occurrence of a common downstream process in bacterial infections. (PDF) [file pone.0089993.s002.pdf]

Additional file. 2

Common pathways mediated by toll-like receptor 2 and 4

Pathway Name	P-value
a6b1 and a6b4 Integrin signaling	0.000209
ALK1 signaling events	0.00831
ALK2 signaling events	0.0409
Alpha4 beta1 integrin signaling events	7.64E-07
Alpha9 beta1 integrin signaling events	0.00447
Alpha-synuclein signaling	1.19E-06
Alternative NF-kappaB pathway	0.0162
amb2 Integrin signaling	3.45E-13
Angiopoietin receptor Tie2-mediated signaling	9.49E-13
AP-1 transcription factor network	3.61E-17
Arf1 pathway	0.0113
Arf6 downstream pathway	0.000013
Arf6 signaling events	0.00192
Arf6 trafficking events	0.0487
ATF-2 transcription factor network	2.75E-08
ATM pathway	1.89E-08
ATR signaling pathway	4.72E-06
Atypical NF-kappaB pathway	9.53E-06
Aurora A signaling	1.19E-06
Aurora B signaling	0.042
BARD1 signaling events	4.94E-10
BCR signaling pathway	2.1E-22
Beta3 integrin cell surface interactions	0.0457
BMP receptor signaling	0.0404
Calcineurin-regulated NFAT-dependent transcription in lymphocy	9.49E-13
Calcium signaling in the CD4+ TCR pathway	1.79E-05
Canonical NF-kappaB pathway	2.27E-10
Caspase Cascade in Apoptosis	9.49E-09
CD40/CD40L signaling	9.09E-08

CDC42 signaling events	1.86E-08
Cellular roles of Anthrax toxin	0.00139
Ceramide signaling pathway	1.17E-14
Circadian rhythm pathway	0.0211
Class I PI3K signaling events	9.49E-13
Class I PI3K signaling events mediated by Akt	4.9E-07
C-MYB transcription factor network	1.1E-10
C-MYC pathway	0.0129
Coregulation of Androgen receptor activity	4.94E-10
CXCR3-mediated signaling events	7.75E-06
CXCR4-mediated signaling events	4.3E-12
Degradation of beta catenin	0.0374
Direct p53 effectors	1.46E-11
Downstream signaling in naïve CD8+ T cells	0.00061
E2F transcription factor network	6.77E-08
E-cadherin signaling in keratinocytes	1.02E-05
E-cadherin signaling in the nascent adherens junction	1.97E-05
EGF receptor (ErbB1) signaling pathway	2.55E-07
EGFR-dependent Endothelin signaling events	0.000324
Endogenous TLR signaling	3.14E-08
Endothelins	0.0001
EPHA forward signaling	7.64E-07
EPHA2 forward signaling	7.27E-07
EPHB forward signaling	1.06E-10
Ephrin B reverse signaling	2.92E-07
EphrinB-EPHB pathway	0.00165
EPO signaling pathway	7.61E-12
ErbB receptor signaling network	0.00136
ErbB1 downstream signaling	1.7E-12
ErbB2/ErbB3 signaling events	2.95E-05
ErbB4 signaling events	3.14E-07
Fanconi anemia pathway	2.78E-07
FAS (CD95) signaling pathway	1.07E-07
Fc-epsilon receptor I signaling in mast cells	4.46E-12

FGF signaling pathway	2.4E-09
FOXA1 transcription factor network	0.000944
FOXA2 and FOXA3 transcription factor networks	2.79E-05
FOXM1 transcription factor network	5.58E-10
FoxO family signaling	8.52E-05
Glucocorticoid receptor regulatory network	6.48E-15
Glypican 1 network	0.000207
GMCSF-mediated signaling events	1.28E-08
Hedgehog signaling events mediated by Gli proteins	0.00272
HIF-1-alpha transcription factor network	9.57E-10
HIF-2-alpha transcription factor network	0.000189
HIV-1 Nef: Negative effector of Fas and TNF-alpha	1.34E-16
Hypoxic and oxygen homeostasis regulation of HIF-1-alpha	7.27E-07
IFN-gamma pathway	2.56E-12
IGF1 pathway	3.51E-06
IL12 signaling mediated by STAT4	2.55E-07
IL12-mediated signaling events	3.89E-11
IL1-mediated signaling events	8.38E-08
IL2 signaling events mediated by PI3K	1.39E-13
IL2 signaling events mediated by STAT5	3.13E-12
IL23-mediated signaling events	3.94E-09
IL27-mediated signaling events	6.48E-07
IL2-mediated signaling events	5.04E-17
IL3-mediated signaling events	0.000533
IL4-mediated signaling events	6.09E-11
IL5-mediated signaling events	4.74E-05
IL6-mediated signaling events	1.16E-16
IL8- and CXCR1-mediated signaling events	0.000511
IL8- and CXCR2-mediated signaling events	0.000125
Insulin Pathway	2.83E-08
Insulin-mediated glucose transport	0.014
Integrin-linked kinase signaling	1.86E-05
Integrins in angiogenesis	0.00487
Internalization of ErbB1	4.69E-05

JNK signaling in the CD4+ TCR pathway	0.000374
Lissencephaly gene (LIS1) in neuronal migration and developmen	0.00924
LKB1 signaling events	0.00109
LPA receptor mediated events	1.52E-09
mTOR signaling pathway	4.5E-09
N-cadherin signaling events	0.0124
Nectin adhesion pathway	0.000206
Nephrin/Neph1 signaling in the kidney podocyte	3.51E-06
Netrin-mediated signaling events	1.19E-06
Neurotrophic factor-mediated Trk receptor signaling	4.61E-07
Noncanonical Wnt signaling pathway	0.000129
Nongenotropic Androgen signaling	1.79E-05
Notch signaling pathway	1.63E-08
Notch-mediated HES/HEY network	5.31E-11
Osteopontin-mediated events	1.19E-08
p38 MAPK signaling pathway	0.0071
p53 pathway	2.18E-09
p73 transcription factor network	1.55E-14
p75(NTR)-mediated signaling	2.68E-07
PAR1-mediated thrombin signaling events	1.21E-05
PAR4-mediated thrombin signaling events	0.0211
PDGFR-alpha signaling pathway	1.37E-06
PDGFR-beta signaling pathway	1.93E-16
Plasma membrane estrogen receptor signaling	1.24E-05
PLK1 signaling events	0.00165
PLK3 signaling events	0.0162
Posttranslational regulation of adherens junction stability and diss	3.32E-08
Presenilin action in Notch and Wnt signaling	2.58E-07
Proteoglycan syndecan-mediated signaling events	0.0267
RAC1 signaling pathway	0.00248
Ras signaling in the CD4+ TCR pathway	0.0343
Reelin signaling pathway	0.00177
Regulation of Androgen receptor activity	2.37E-08
Regulation of cytoplasmic and nuclear SMAD2/3 signaling	9.87E-05

Regulation of nuclear beta catenin signaling and target gene trans	1.24E-05
Regulation of nuclear SMAD2/3 signaling	3.61E-23
Regulation of p38-alpha and p38-beta	0.000319
Regulation of RAC1 activity	0.00339
Regulation of retinoblastoma protein	5.88E-16
Regulation of Telomerase	2.04E-14
Retinoic acid receptors-mediated signaling	1.11E-11
RhoA signaling pathway	0.00165
Role of Calcineurin-dependent NFAT signaling in lymphocytes	0.0176
RXR and RAR heterodimerization with other nuclear receptor	1.95E-10
S1P1 pathway	3.22E-05
S1P2 pathway	6.48E-07
S1P3 pathway	9.21E-07
S1P4 pathway	0.000374
S1P5 pathway	0.0481
SHP2 signaling	1.05E-12
Signaling events mediated by focal adhesion kinase	1.09E-08
Signaling events mediated by HDAC Class I	1.58E-11
Signaling events mediated by HDAC Class II	3.14E-05
Signaling events mediated by HDAC Class III	4.61E-12
Signaling events mediated by Hepatocyte Growth Factor Recepto	1.78E-17
Signaling events mediated by PRL	0.00213
Signaling events mediated by PTP1B	1.97E-13
Signaling events mediated by Stem cell factor receptor (c-Kit)	1.86E-16
Signaling events mediated by TCPTP	1.71E-10
Signaling events mediated by VEGFR1 and VEGFR2	1.08E-09
Signaling events regulated by Ret tyrosine kinase	4.61E-12
Signaling mediated by p38-alpha and p38-beta	3.14E-07
Sphingosine 1-phosphate (S1P) pathway	0.00344
Stabilization and expansion of the E-cadherin adherens junction	0.000944
Sumoylation by RanBP2 regulates transcriptional repression	0.000113
Syndecan-2-mediated signaling events	1.13E-05
Syndecan-4-mediated signaling events	6.41E-06
TCR signaling in naïve CD4+ T cells	1.34E-15

TCR signaling in naïve CD8+ T cells	1.05E-09
TGF-beta receptor signaling	1.19E-06
Thromboxane A2 receptor signaling	6.62E-06
TNF receptor signaling pathway	1.07E-09
TRAIL signaling pathway	7.81E-05
Trk receptor signaling mediated by PI3K and PLC-gamma	7.81E-05
Trk receptor signaling mediated by the MAPK pathway	0.00993
Urokinase-type plasminogen activator (uPA) and uPAR-mediated	0.00878
Validated nuclear estrogen receptor alpha network	3.5E-10
Validated nuclear estrogen receptor beta network	0.00378
Validated targets of C-MYC transcriptional activation	5.31E-08
Validated targets of C-MYC transcriptional repression	2.54E-07
Validated transcriptional targets of AP1 family members Fra1 an	2.7E-08
Validated transcriptional targets of deltaNp63 isoforms	0.000133
Validated transcriptional targets of TAp63 isoforms	0.000407
VEGFR1 specific signals	2.92E-07
VEGFR3 signaling in lymphatic endothelium	0.0129
Wnt signaling network	0.036
